# Beclin 1 prevents ISG15-mediated cytokine storms to secure fetal hematopoiesis and survival

**DOI:** 10.1172/JCI177375

**Published:** 2025-02-03

**Authors:** Wen Wei, Xueqin Gao, Jiawei Qian, Lei Li, Chen Zhao, Li Xu, Yanfei Zhu, Zhenzhen Liu, Nengrong Liu, Xueqing Wang, Zhicong Jin, Bowen Liu, Lan Xu, Jin Dong, Suping Zhang, Jiarong Wang, Yumu Zhang, Yao Yu, Zhanjun Yan, Yanjun Yang, Jie Lu, Yixuan Fang, Na Yuan, Jianrong Wang

**Affiliations:** 1 Research Center for Blood Engineering and Manufacturing, Cyrus Tang Medical Institute, Soochow University, Suzhou, China.; 2 National Clinical Research Center for Hematologic Diseases, Key Laboratory of Thrombosis and Hemostasis Ministry of Health, Collaborative Innovation Center of Hematology, Jiangsu Institute of Hematology, Institute of Blood and Marrow Transplantation, The First Affiliated Hospital of Soochow University, Suzhou, China.; 3 State Key Laboratory of Radiation Medicine and Protection, Soochow University, Suzhou, China.; 4 The Ninth Affiliated Suzhou Hospital of Soochow University, Suzhou, China.

**Keywords:** Hematology, Immunology, Autophagy, Cellular immune response, Hematopoietic stem cells

## Abstract

Proper control of inflammatory responses is essential for embryonic development, but the underlying mechanism is poorly understood. Here, we show that under physiological conditions, inactivation of ISG15, an inflammation amplifier, is associated with the interaction of Beclin 1 (Becn1), via its evolutionarily conserved domain, with STAT3 in the major fetal hematopoietic organ of mice. Conditional loss of *Becn1* caused sequential dysfunction and exhaustion of fetal liver hematopoietic stem cells, leading to lethal inflammatory cell–biased hematopoiesis in the fetus. Molecularly, the absence of *Becn1* resulted in the release of STAT3 from Becn1 tethering and subsequent phosphorylation and translocation to the nucleus, which in turn directly activated the transcription of *ISG15* in fetal liver hematopoietic cells, coupled with increased ISGylation and production of inflammatory cytokines, whereas inactivating STAT3 reduced *ISG15* transcription and inflammation but improved hematopoiesis potential, and further silencing *ISG15* mitigated the above collapse in the *Becn1*-null hematopoietic lineage. The Becn1/STAT3/ISG15 axis remains functional in autophagy-disrupted fetal hematopoietic organs. These results suggest that Becn1, in an autophagy-independent manner, secures hematopoiesis and survival of the fetus by directly inhibiting STAT3/ISG15 activation to prevent cytokine storms. Our findings highlight a previously undocumented role of Becn1 in governing ISG15 to safeguard the fetus.

## Abstract 2

Becn1 secures hematopoiesis and survival of the fetus by directly inhibiting STAT3-ISG15 activation to preclude cytokine storms.

## Graphical abstract

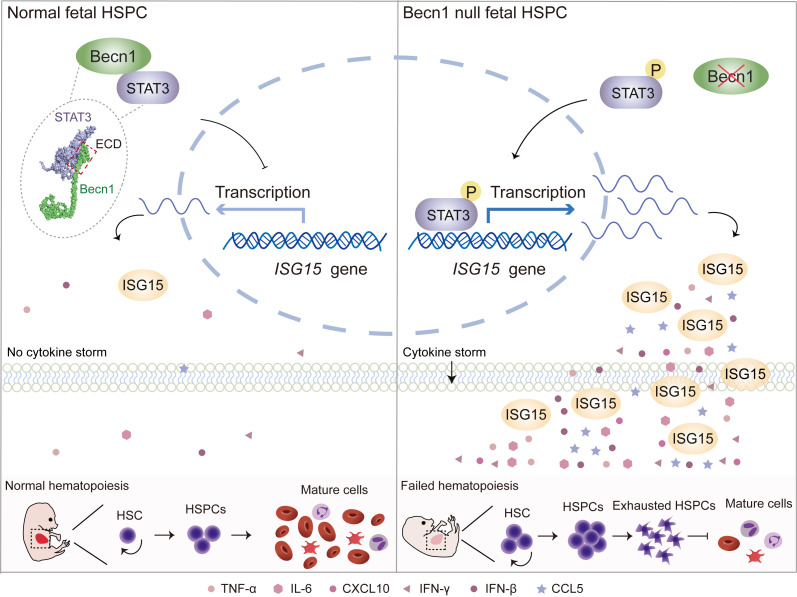

## Introduction

Mammalian fetal hematopoiesis initiates in the yolk sac, known as primitive hematopoiesis, which is rapidly replaced by definitive hematopoiesis in aorta-gonad-mesonephros commencing at embryonic day 10.5 (E10.5) in mice. Soon after, hematopoietic stem cells (HSCs) generated in aorta-gonad-mesonephros migrate to the fetal liver for massive expansion and gradual maturation, with the most active stage of fetal liver hematopoiesis on E14.5. The liver remains the major organ of fetal hematopoiesis until HSCs start settling to the bone marrow on E16.5 to E17.5. The bone marrow becomes the main fetal hematopoietic organ from E18.5 to birth (1–5). In human embryos between 7 and 17 weeks after conception, the liver serves as the predominant organ of fetal hematopoiesis, covering the second trimester of human embryonic development (4, 6–8).

Interferon-stimulated gene 15 (ISG15), a ubiquitin-like protein and an amplifier of inflammation, can be induced by interferon (IFN) (9), viral and bacterial infections (10, 11), or certain genotoxic stressors (12), indicating that the expression of ISG15 represents a host response to pathogenic insults. Mechanistically, type I IFN activates IFN regulatory factor 9, which subsequently interacts with 2 members of the phosphorylated signal transducer and activator of transcription family (STAT1 and STAT2) and forms the IFN-stimulated gene factor 3 complex, which binds, via an IFN-sensitive response element, to the promoter of ISG15 and its conjugation enzymes to initiate their expression. ISG15 can be covalently conjugated onto hundreds of target proteins via an enzymatic cascade to enhance numerous biological responses, including cytokine expression and the inflammatory response against invading pathogens (13, 14). ISG15 in its unconjugated free form has also been reported to function as a cytokine. For example, extracellularly unconjugated ISG15 can act as a cytokine to exacerbate SARS-CoV-2–triggered cytokine storms and inflammation (15, 16). However, how ISG15 expression and activation are tightly controlled to prevent intrinsic and extrinsic cytokine storms awaits exploration.

Beclin 1 (Becn1), the first identified autophagy protein in mammals, plays a key role in mammalian autophagy (17). In launching autophagy, Becn1 binds Vps34, UVRAG, AMBRA-1, and Barkor to form the PI3KC3 complex, a core assembly of the mammalian autophagy machinery (18–21). Becn1 has been reported to play an important role in many physiological or pathological processes, such as tumorigenesis, anti-aging, and AMPK activation, almost all via the autophagy pathway (22–26), with the exceptions of its ability to uncouple autophagy in endocytic trafficking (27–29), hormone secretion (30), and receptor recycling (31). In addition, Becn1 functions as a negative regulator in the execution of necroptosis by suppressing MLKL oligomerization (32). With respect to blood function in adults, mice with myeloid-specific loss of Becn1 exhibited neutrophilia, suggesting that Becn1 can act as a neutrophil-specific immune checkpoint (33). Becn1 in mice was also found to maintain the quiescence of tissue-resident macrophages to resist *Listeria monocytogenes* infection (34). However, the role of Becn1 in fetal hematopoiesis has not been examined.

Here, we generated a conditional mouse with hematopoietic-lineage deletion of *Becn1* and report that Becn1 is essential for fetal hematopoiesis and survival by tightly controlling inflammatory cytokine production in the fetal hematopoietic organ.

## Results

### Hematopoietic loss of Becn1 causes severe inflammatory cell–biased hematopoiesis and complete perinatal lethality.

**Figure 1:**
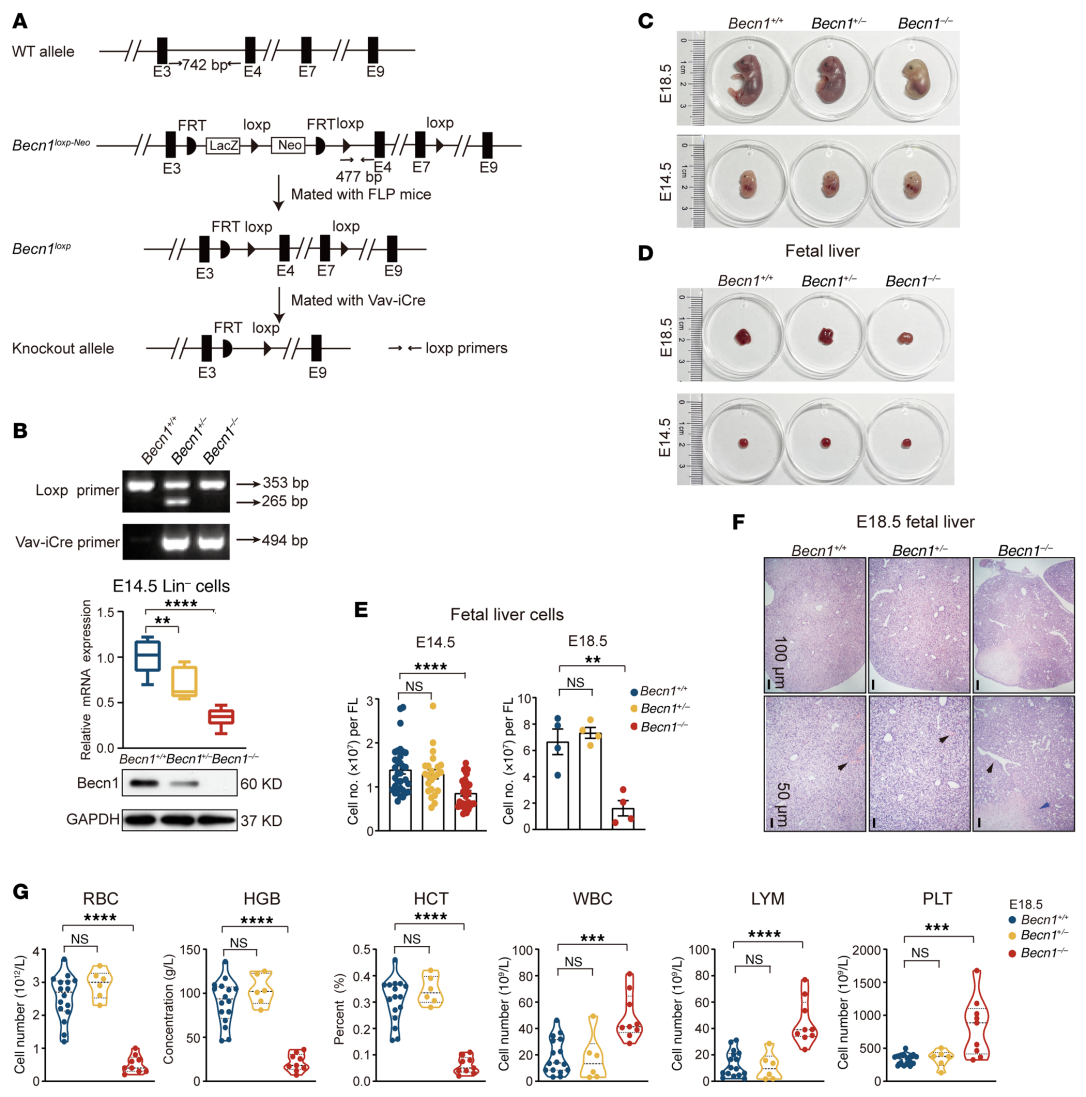
Deletion of *Becn1* in the hematopoietic system causes severe immune cell–biased hematopoiesis. (**A**) Generation of *Becn1*-floxed and *Becn1^fl/fl^* Vav-iCre mice. The schematic diagram illustrates the strategy to delete *Becn1*. *LoxP* sites are located on exon 4 and exon 7. (**B**) Genotyping of *Becn1*-deleted mice. Top: PCR analysis of tail genomic DNA. Bottom: *Becn1* mRNA expression in E14.5 fetal liver Lin^–^ cells (*n* = 8–9). mRNA levels were normalized to *Gapdh* expression. Western blots for Becn1 and GAPDH in E14.5 fetal liver Lin^–^ cells. (**C** and **D**) Representative images of *Becn1^+/+^*, *Becn1^+/–^*, and *Becn1^–/–^* fetuses and fetal livers at E18.5 and E14.5. (**E**) Total liver cell numbers in E14.5 and E18.5 fetal livers. FL, fetal livers. E14.5, *n* = 23–33; E18.5, *n* = 4. (**F**) Representative H&E staining of fetal liver sections from E18.5 embryos. Black arrows indicate a lack of erythroid cells in the blood vessels of *Becn1^–/–^* fetal livers. Blue arrows indicate focal necrosis. Scale bars: 100 μm, top; 50 μm, bottom. (**G**) Peripheral blood cell parameters from E18.5 embryos (*n* = 6–16), including red blood cells (RBC), hemoglobin (HGB), hematocrit (HCT), white blood cells (WBC), lymphocytes (LYM), and platelets (PLT). **P < 0.01; ***P < 0.001; ****P < 0.0001, 1-way ANOVA (Dunnett’s multiple-comparison test). Data represent the mean ± SEM.

To define the role of Becn1 in fetal hematopoiesis, we generated *Becn1^fl/fl^* mice by gene targeting and mated them with Vav-iCre transgenic mice (Figure 1A and Supplemental Figure 1A; supplemental material available online with this article; https://doi.org/10.1172/JCI177375DS1). Following genotyping of the offspring mice with tail DNA, phenotyping of their fetuses at the transcriptional and translational levels indicated successful deletion of *Becn1* in the lineage-negative (Lin^–^) hematopoietic cells of the liver, the major fetal hematopoietic organ (Figure 1B), but did not alter Becn1 levels in non-hematopoietic tissues (Supplemental Figure 1B). Exclusive deletion of biallelic *Becn1* in hematopoietic lineages caused 100% perinatal death (Table 1).

E14.5 and E18.5 roughly represent the most active stage and the end of fetal liver hematopoiesis, respectively, in mice. Unlike *Becn1^–/–^* embryos, whose weight was unchanged at E14.5 but reduced at E18.5 (Supplemental Figure 1C), the embryos appeared paler and smaller at E14.5 and E18.5 (Figure 1C). At E14.5 and E18.5, despite the unchanged fetal liver weight of *Becn1^–/–^* mice (Supplemental Figure 1D), the fetal liver size was reduced (Figure 1D), and the total liver cell number was diminished (Figure 1E). Histological assays revealed a lack of erythroid cells in the blood vessels and focal necrosis in E18.5 *Becn1^–/–^* fetal livers (Figure 1F) and decreased blood cells in the E18.5 marrow cavity in *Becn1^–/–^* embryos (Supplemental Figure 1E). In addition, the fetal spleen and thymus, the other two extramedullary hematopoietic organs, decreased in weight in *Becn1^–/–^* embryos (Supplemental Figure 1F). In contrast, *Becn1^+/–^* embryos did not present an abnormal phenotype. These results indicate that all of the fetal hematopoietic organs were impaired in *Becn1^–/–^* but not *Becn1^+/–^* mice.

Analysis of peripheral blood from E18.5 embryos revealed that red blood cells, hemoglobin, and hematocrit in *Becn1^–/–^* embryos were decreased, but white blood cells, lymphocytes, and platelets were increased (Figure 1G). These results indicate that conditional deletion of biallelic *Becn1* severely impairs fetal hematopoiesis, which is characterized by anemia and a dramatic increase in white blood cells, particularly lymphocytes and platelets. Decontrolled production of white blood cells and platelets is often connected to high inflammation (35–37). Therefore, increased inflammation may be responsible for hematopoiesis failure and perinatal death in *Becn1^–/–^* embryos.

### The absence of Becn1 results in sequential dysfunction and exhaustion of fetal liver HSCs.

**Figure 2:**
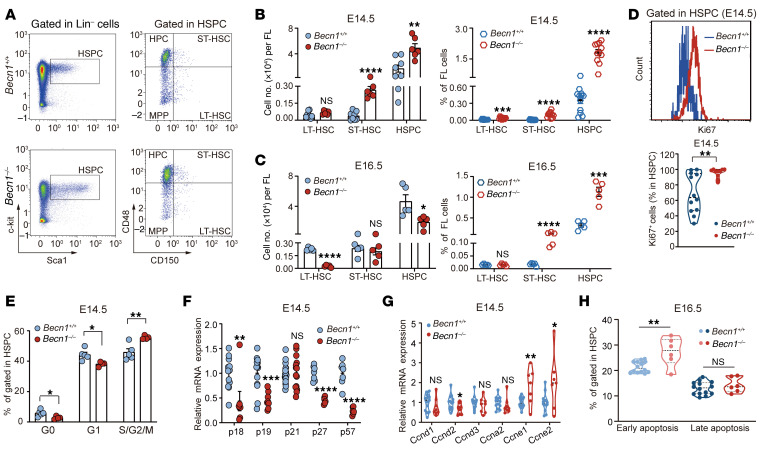
Deletion of *Becn1* leads to exhaustion of fetal liver HSCs. (**A**) Representative flow cytometric analysis plot of HSCs and HSPCs in the fetal livers of *Becn1^+/+^* and *Becn1^–/–^* mice. (**B** and **C**) Absolute numbers (left) and percentages (right) of HSCs and HSPCs in the fetal livers of *Becn1^+/+^* and *Becn1^–/–^* E14.5 mice (*n* = 6–12) and E16.5 mice (*n* = 5) were detected via flow cytometry. (**D**) Representative flow cytometric analysis of Ki67 expression (top) and the percentage of Ki67^+^ cells in HSPCs (bottom) detected in *Becn1^+/+^* and *Becn1^–/–^* E14.5 fetal liver cells (FLCs) (*n* = 10–12). (**E**) Cell cycle analysis of HSPCs from *Becn1^+/+^* and *Becn1^–/–^* E14.5 FLCs (*n* = 4 or 5). (**F**) Real-time qPCR analysis of the transcription of cycling-dependent kinase inhibitor genes in the HSPCs of E14.5 FLCs. The mRNA levels were normalized to *Gapdh* expression (*n* = 6–20). (**G**) Real-time qPCR analysis of the expression of cyclins (*n* = 7–13). (**H**) Apoptosis analysis of HSPCs from *Becn1^+/+^* and *Becn1^–/–^* E16.5 fetal livers (*n* = 6–14). *P < 0.05; **P < 0.01; ***P < 0.001; ****P < 0.0001. Unpaired 2-tailed Student’s *t* test. Data represent the mean ± SEM.

To explore the cause of fetal hematopoiesis failure by *Becn1* deletion, we identified hematopoietic stem cells (HSCs; Lin^–^Sca1^+^c-Kit^+^CD48^–^CD150^+^) and hematopoietic progenitors in the fetal liver, the major hematopoietic organ of the fetus in mice. For this purpose, we harvested fetal liver cells (FLCs) from *Becn1^+/+^* and *Becn1^–/–^* embryos via flow cytometry. *Becn1^–/–^* fetal livers at E12.5 presented no detectable changes in long-term HSCs (LT-HSCs; Lin^–^Sca1^+^c-Kit^+^CD48^–^CD150^+^), short-term HSCs (ST-HSCs; Lin^–^Sca1^+^c-Kit^+^CD48^+^CD150^+^), hematopoietic stem and progenitor cells (HSPCs; Lin^–^Sca1^+^c-Kit^+^), and major progenitors, including multipotent progenitors (MPs; Lin^–^Sca1^–^c-Kit^+^) and common lymphoid progenitors (CLPs; Lin^–^IL-7Rα^+^Sca1^lo^c-Kit^lo^) (Supplemental Figure 2, A and B), but at E14.5, the number and percentage of HSCs and HSPCs were increased (Figure 2, A and B), associated with a slightly decreased number of MPs and common myeloid progenitors (CMPs; Lin^–^Sca1^–^c-Kit^+^CD34^+^CD16/32^–^) but slightly increased CLPs, suggesting lymphoid-biased fetal hematopoiesis (Supplemental Figure 2, C and D). At E16.5, the late stage of fetal liver hematopoiesis, the number of LT-HSCs and HSPCs dramatically decreased, but the percentage of ST-HSCs and HSPCs in total FLCs increased because of a significant reduction in total liver cells (Figure 2C), and most of the progenitor cells, including MPs, CMPs, granulocyte-macrophage progenitors (GMPs; Lin^–^Sca1^–^c-Kit^+^CD16/32^+^CD34^+^), and megakaryocyte-erythroid progenitors (MEPs; Lin^–^Sca1^–^c-Kit^+^CD16/32^–^CD34^–^), further decreased, with the exception of CLPs, suggesting lasting lymphoid-biased fetal hematopoiesis (Supplemental Figure 2E). Proliferation and cell cycle analysis revealed a marked shift from G_0_/G_1_ to S/G_2_/M, suggesting disrupted quiescence and increased cycling of *Becn1^–/–^* HSPCs in E14.5 fetal livers (Figure 2, D and E, and Supplemental Figure 2F), which is associated with downregulated expression of cyclin-dependent kinase inhibitors, including p18, p19, p27, and p57, but upregulated expression of positive cell cycle regulators, such as *Ccne1* and *Ccne2* (Figure 2, F and G). In contrast to the lack of increased apoptosis at E14.5 (Supplemental Figure 2, G and H), apoptosis and oxidative reactive oxygen species levels were elevated in *Becn1^–/–^* HSPCs at E16.5 (Figure 2H and Supplemental Figure 2J), although Ki67 staining remained high (Supplemental Figure 2I). Overall, these results suggest that fetal HSCs and progenitor cells started dysplasia with abnormal self-renewal or proliferation at E14.5 and then quickly became exhausted at E16.5 in the hematopoietic *Becn1^–/–^* embryos because of faster proliferation and faster death. Consequently, erythropoiesis and myelopoiesis were impaired in the livers of *Becn1^–/–^* fetuses at E14.5 or E16.5 (Supplemental Figure 3).

**Figure 3:**
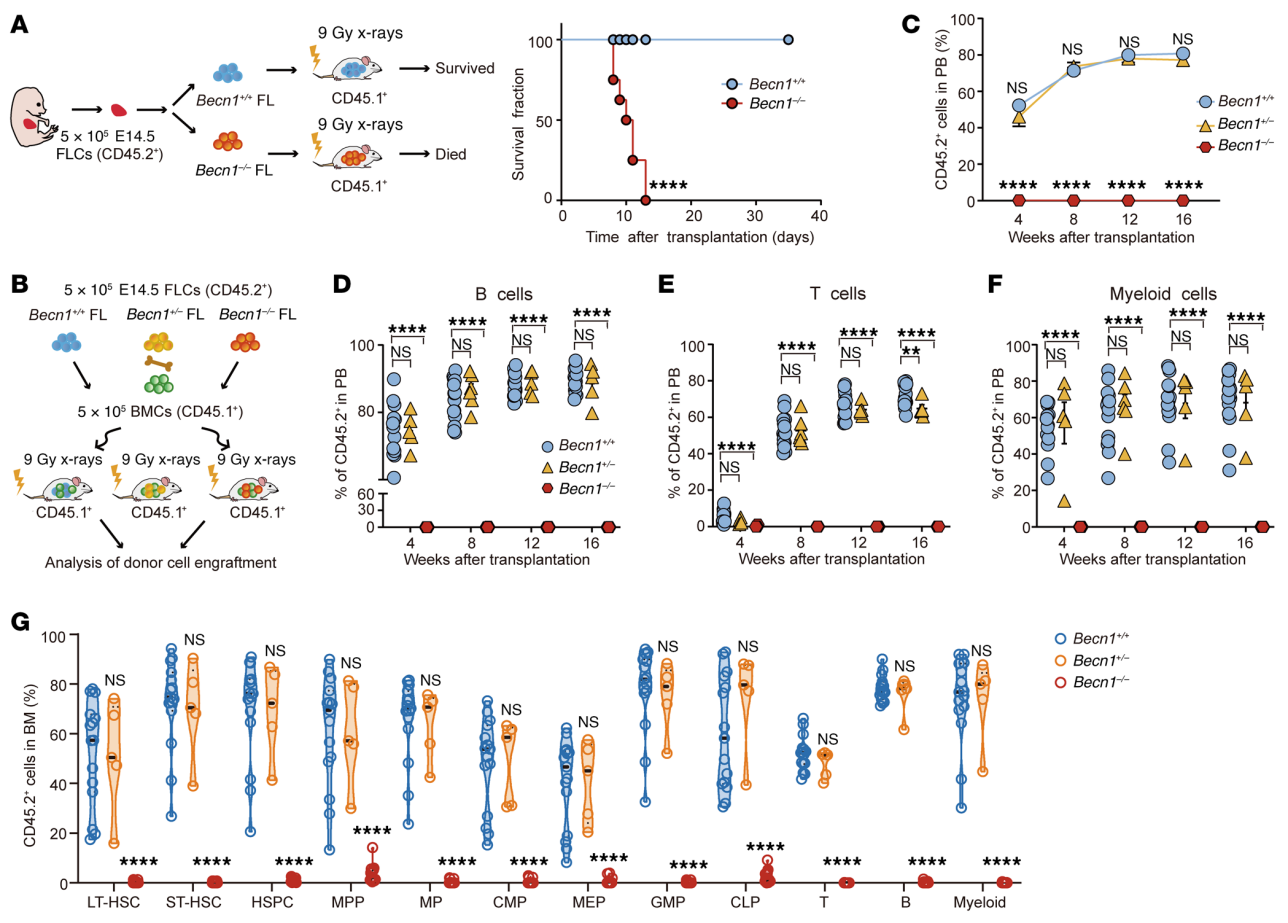
Deletion of *Becn1* leads to reconstitution failure of fetal liver HSCs. (**A**) Left: Schematic plan for FLC transplantation. Right: *Becn1^–/–^* CD45.2^+^ liver hematopoietic cells failed to rescue lethally irradiated wild-type CD45.1^+^ recipient mice. Log-rank test. *n* = 7–8. (**B**) Schematic plan of competitive FLC transplantation. (**C**–**F**) Peripheral blood counts from recipient mice analyzed by flow cytometry at 4, 8, 12, and 16 weeks after competitive transplantation. B cells (B220^+^), T cells (CD3^+^), and myeloid cells (Gr1^+^CD11b^+^). *n* = 5–17. (**G**) Donor-derived (CD45.2^+^) cells in specific cell populations in the recipients’ bone marrow were detected by flow cytometry at 16 weeks after competitive FLC transplantation from E14.5 mice (*n* = 5–17). **P < 0.01; ****P < 0.0001. Survival curves were compared using the log-rank test. Statistical analyses were performed via 1-way ANOVA (Dunnett’s multiple-comparison test). Data represent the mean ± SEM.

To evaluate the damage to fetal hematopoiesis caused by *Becn1* deletion, we performed colony formation assays. When cultured in methylcellulose medium containing IL-6, IL-3, SCF, and erythropoietin, the number of colonies formed by *Becn1^–/–^* FLCs was significantly lower than that formed by E14.5 *Becn1^+/+^* or *Becn1^+/–^* FLCs (Supplemental Figure 4, A and B). In contrast to the fully rescued irradiated recipients resulting from the transplantation of *Becn1^+/+^* FLCs, all the recipients died within 13 days after the transplantation of E14.5 *Becn1^–/–^* FLCs (Figure 3A). These results indicate that the loss of Becn1 severely impaired hematopoietic differentiation at E14.5.

To assess the impact of the absence of Becn1 on long-term hematopoietic reconstitution capacity, we performed competitive FLC transplantation by injecting CD45.2^+^ *Becn1^+/+^*, *Becn1^+/–^*, or *Becn1^–/–^* FLCs from E14.5 embryos mixed with the same number of CD45.1^+^ bone marrow cells into lethally irradiated CD45.1^+^ recipient mice and then analyzed donor-derived cells in peripheral blood and bone marrow (Figure 3B and Supplemental Figure 4C). Competitive transplantation assays revealed that CD45.2^+^ cells from E14.5 *Becn1^–/–^* FLCs were undetectable in the peripheral blood of the irradiated recipients (Figure 3C and Supplemental Figure 4D), and similarly, similarly, B cells (B220^+^ cells), T cells (CD3^+^ cells) and myeloid cells (Gr1^+^CD11b^+^ cells) derived from *Becn1^–/–^* FLCs were also undetectable at 4, 8, 12, and 16 weeks after transplantation (Figure 3, D–F). Similarly, at 16 weeks, no contribution of E14.5 *Becn1^–/–^* FLCs to HSCs and their downstream progenitors and differentiated lineages in the recipients’ bone marrow was detected (Figure 3G and Supplemental Figure 4D). These results indicate that loss of Becn1 impairs the function of HSCs at E14.5 and leads to HSC exhaustion at E16.5, indicating an indispensable role of Becn1 in the reconstitution of hematopoietic lineages of fetal liver HSCs.

### Multiomics profiling reveals upregulated proinflammatory responses featuring high ISG15 expression in fetal hematopoietic organs due to Becn1 deletion.

**Figure 4:**
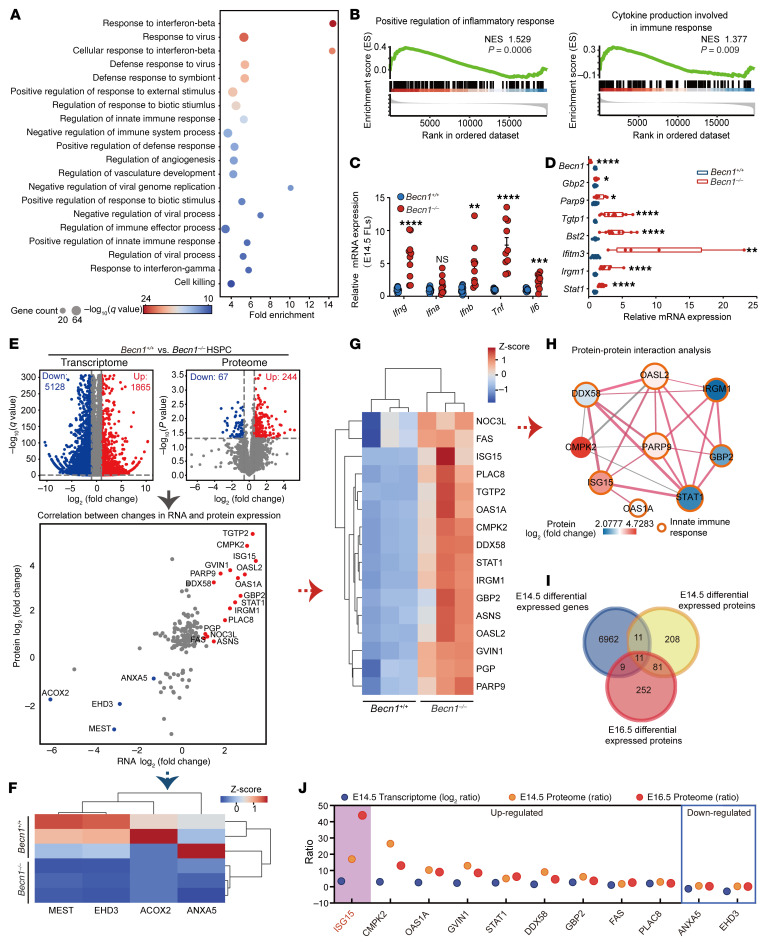
Multiomics profiling reveals decontrolled innate immune response in *Becn1*-deleted fetal liver hematopoietic cells. (**A**) Bubble plot showing GO enriched pathways of E14.5 HSPC RNA sequencing (FPKM > 5, in at least 1 sample). *n* = 3. (**B**) Gene set enrichment analysis of inflammatory signaling pathways in *Becn1^+/+^* and *Becn1^–/–^* HSPCs. (**C**) Real-time qPCR analysis of the expression of inflammatory factors in E14.5 fetal livers (*n* = 10–12). The mRNA levels were normalized to *Gapdh* expression. (**D**) Relative expression levels of representative genes in the IFN-related pathway determined by real-time qPCR analysis in E14.5 HSPCs. The mRNA levels were normalized to *Gapdh* expression (*n* = 5–12). (**E**) Volcano plots of the changes in mRNA levels (top left, *n* = 3) and protein levels (top right, *n* = 4) in *Becn1^+/+^* and *Becn1^–/–^* HSPCs from E14.5 fetal livers and correlations between changes in protein and mRNA levels (bottom). (**F**) Heatmap analysis of downregulated genes among the correlated genes from **E**. (**G**) Heatmap analysis of upregulated genes among the correlated genes from **E**. (**H**) Protein-protein interaction analysis of upregulated correlated proteins via the STRING database via Cytoscape (https://cytoscape.org). (**I**) Intersection analysis of E14.5 and E16.5 differentially expressed proteins from proteomics compared with differentially expressed genes from E14.5 transcriptomics. (**J**) The differential ratio change of 11 intersecting proteins from **I**. *P < 0.05; **P < 0.01; ***P < 0.001; ****P < 0.0001. Unpaired 2-tailed Student’s *t* test. Data represent the mean ± SEM.

To understand the mechanism by which Becn1 regulates fetal hematopoiesis, we performed transcriptomics and proteomics on sorted LSK HSPCs (Lin^–^Sca1^+^c-Kit^+^) from E14.5 *Becn1^+/+^* and *Becn1^–/–^* fetal livers. Gene Ontology (GO) enrichment analysis of the transcriptome revealed that functions related to the innate immune response were significantly enriched (Figure 4A). Gene set enrichment analysis (GSEA) revealed the upregulation of numerous innate immune and inflammation-related processes, such as positive regulation of the inflammatory response, cytokine production involved in the immune response, regulation of the innate immune response, and the JAK/STAT signaling pathway (Figure 4B and Supplemental Figure 5, A and B). Moreover, GSEA revealed that processes associated with the cell response to IFN-α, -β, and -γ were significantly upregulated (Supplemental Figure 5C). Gene expression analysis revealed obvious activation of IFN signaling (Supplemental Figure 5D). The increased expression of inflammatory factors and IFN-related genes (selected from Supplemental Figure 5D) in E14.5 *Becn1^–/–^* FLCs was confirmed by real-time quantitative PCR (qPCR) (Figure 4, C and D). These results suggest that the absence of Becn1 caused an overactivated innate immune response and inflammation, which may be responsible for fetal hematopoiesis failure and perinatal death.

Proteomic profiling further revealed a highly upregulated innate immune response and inflammatory signaling, which was consistent with the transcriptomic disclosure (Supplemental Figure 6A). To further dissect the molecular mechanism responsible for this destruction, we performed a combinatory transcriptomics and proteomics analysis. Volcano plots revealed 5,128 downregulated and 1,865 upregulated differentially expressed genes in the E14.5 liver HSPC transcriptome, with 67 downregulated and 244 upregulated differentially expressed proteins in the E14.5 liver HSPC proteome (Figure 4E, top), whereas in the E16.5 proteome, there were 79 downregulated and 274 upregulated differentially expressed proteins (Supplemental Figure 6B). Analysis of the differentially expressed genes and proteins of the E14.5 transcriptome and proteome revealed 16 upregulated proteins or genes (in red) and 4 downregulated proteins or genes (in blue) (Figure 4E, bottom). Heatmap analysis revealed 4 co-downregulated genes or proteins and 16 co-upregulated genes or proteins (Figure 4, F and G). STRING analysis revealed that most of the co-upregulated proteins strongly interacted with one another, and GO enrichment analysis suggested that these proteins participated in the innate immune response together (Figure 4H). Similarly, GO enrichment analysis of correlated genes or proteins from E14.5 transcriptomics and proteomics also revealed enriched processes related to innate immunity and inflammation (Supplemental Figure 6C).

To explore the target molecules of Becn1 in regulation of fetal hematopoiesis, we analyzed the intersection of differentially expressed genes from the E14.5 transcriptome and differentially expressed proteins from the E14.5 and E16.5 proteomes, which revealed 11 genes or proteins sharing common changes (Figure 4I). Among the 11 intersecting proteins, 9 were upregulated, and 2 were downregulated. Notably, we observed that ISG15, a 15 kDa ubiquitin protein, exhibited a 17-fold change in the E14.5 proteome and a 43-fold change in the E16.5 proteome due to the loss of *Becn1* (Figure 4J). Multicolor cytometric analysis of E14.5 FLCs revealed that LSK HSPCs constituted the major cell population that expressed inflammatory cytokines, particularly ISG15, and that CD11b^+^ myeloid cells also expressed cytokines, excluding ISG15, when *Becn1* was deleted (Supplemental Figure 7).

### Becn1 deficiency causes ISG15 activation, and disruption of ISG15 rescues hematopoiesis and reduces inflammation in fetal hematopoietic organs.

**Figure 5:**
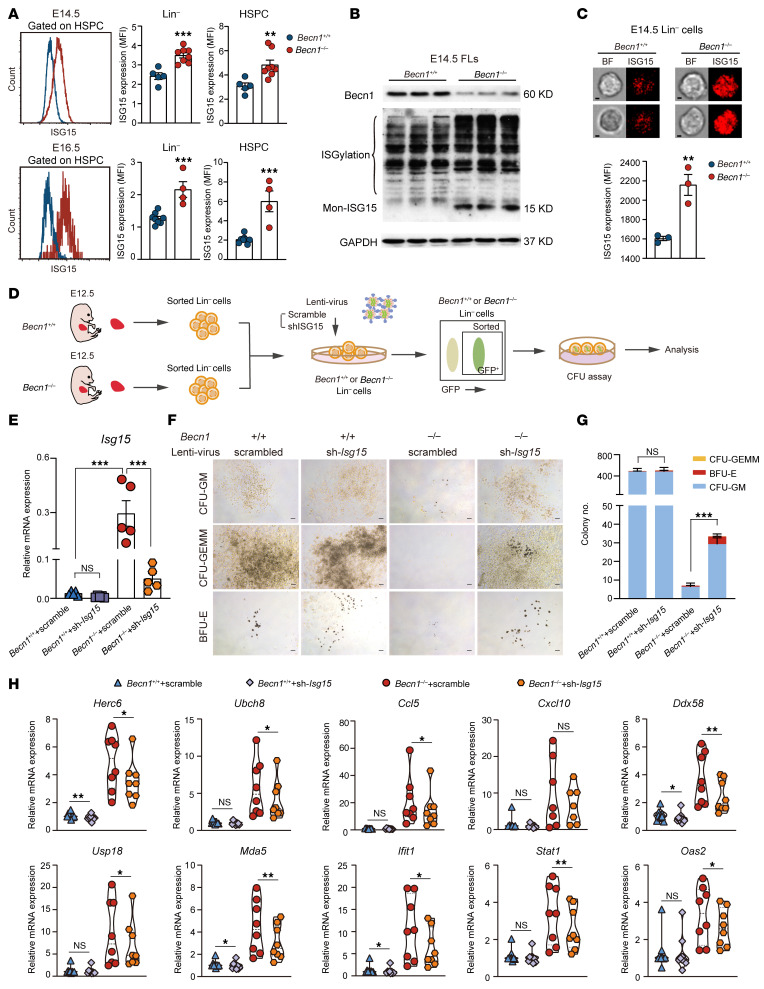
Deletion of *Becn1* increases ISG15, the amplifier of inflammation, and disruption of ISG15 partially rescues hematopoietic failure in *Becn1*-deleted mice. (**A**) Flow cytometric analysis of ISG15 levels in E14.5/E16.5 Lin^–^ cells and HSPCs (*n* = 4–8). (**B**) ISG15 protein levels detected by Western blotting in E14.5 fetal livers. (**C**) Imaging flow cytometry detection of ISG15 levels in E14.5 fetal liver Lin^–^ cells (*n* = 3). Top: Representative imaging flow cytometry pictures of ISG15 protein levels. Bottom: Statistical results of the imaging flow cytometry detection. BF, bright field. Scale bar: 2.5 μm. (**D**) Schematic plan of lentivirus-infected Lin^–^ cells and colony formation assays in E12.5 FLCs. (**E**) Real-time qPCR analysis of *ISG15* expression in GFP^+^ cells after sorting of lentivirus-infected Lin^–^ cells from E12.5 FLCs (*n* = 5). (**F** and **G**) Determination of the effect of *ISG15* knockdown on colony formation in *Becn1^–/–^* and *Becn1^+/+^* E12.5 Lin^–^ FLCs (*n* = 5). Representative pictures of colonies formed from GFP^+^ cells after sorting of lentivirus-infected E12.5 Lin^–^ cells in FLCs (**F**) and analysis of counted colonies (**G**). Scale bars: 100 μm. (**H**) Real-time qPCR analysis of ISG15-related genes and inflammation-related genes in GFP^+^ cells after sorting of lentivirus-infected Lin^–^ cells from E12.5 FLCs (*n* = 7–9). *P < 0.05; **P < 0.01; ***P < 0.001. Unpaired 2-tailed Student’s *t* test (**A**, **C**, and **G**); 1-way ANOVA (Tukey’s multiple-comparison test) (**E**); paired 2-tailed Student’s *t* test (**H**). Data represent the mean ± SEM.

As a ubiquitin-like protein, ISG15 can ISGylate a long array of proteins to upregulate innate immune responses and inflammation. Given that *Becn1* deletion significantly increased inflammatory immune cells and caused hematopoiesis failure (Figures 1–3), together with the multiomics information revealing the activation of ISG15 (Figure 4), we hypothesized that ISG15 is a potential target of Becn1 in regulation of fetal hematopoiesis. To address this, we validated the increased expression of ISG15 in E14.5 HSPCs by flow cytometry (Figure 5A). Western blotting further revealed increases in the levels of ISG15, including its monomeric form and ISGylation, in E14.5 and E16.5 *Becn1^–/–^* FLCs (Figure 5B and Supplemental Figure 8A). Consistent with the above observations, image flow cytometry analysis revealed a visual and quantified increase in ISG15 in E14.5 *Becn1^–/–^* Lin^–^ FLCs (Figure 5C).

To determine whether upregulated ISG15 is responsible for the activation of the innate immune response and inflammation, which contributes to hematopoietic failure, we sought to rescue the function of hematopoietic progenitor cells via *ISG15* knockdown in *Becn1^–/–^* fetal livers. We performed lentiviral transduction of *Becn1^–/–^* Lin^–^ FLCs with *ISG15* short hairpin RNA (shRNA) or control shRNA to test their colony-forming ability (Figure 5D). *ISG15* was effectively knocked down, as shown by real-time qPCR (Figure 5E and Supplemental Figure 8B). *ISG15* knockdown partially but significantly rescued colony formation in *Becn1^–/–^* Lin^–^ FLCs (Figure 5, F and G).

To determine whether *ISG15* knockdown could alleviate the innate immune response and inflammation, we first analyzed the putative ISG15 pull-down proteins, which are ISG15-targeting proteins suggested by documented proteomics data (38), together with differentially expressed genes in the E14.5 *Becn1^–/–^* fetal liver HSPC transcriptome of our analysis, which revealed the intersecting differentially expressed genes by cluster analysis (Supplemental Figure 9A and Supplemental Table 3). GO enrichment analysis revealed that most biological functions related to the innate immune response and inflammation were significantly enriched and upregulated (Supplemental Figure 9B, orange). We noted the genes that participate in enriched biological functions on the heatmap by red dots (Supplemental Figure 9A). We found that most of these upregulated genes can positively regulate the innate immune response and inflammation. Next, we detected some of these upregulated genes from Supplemental Figure 9A (Supplemental Figure 9A, blue dots) after *ISG15* knockdown in *Becn1^–/–^* fetal liver Lin^–^ cells by real-time qPCR, and the results revealed decreased expression of these proinflammatory genes, thereby suggesting a partially alleviated innate immune response and inflammation (Figure 5H).

### Loss of Becn1 prompts STAT3 to transcriptionally activate ISG15, contributing to inflammation and fetal hematopoiesis failure.

**Figure 6:**
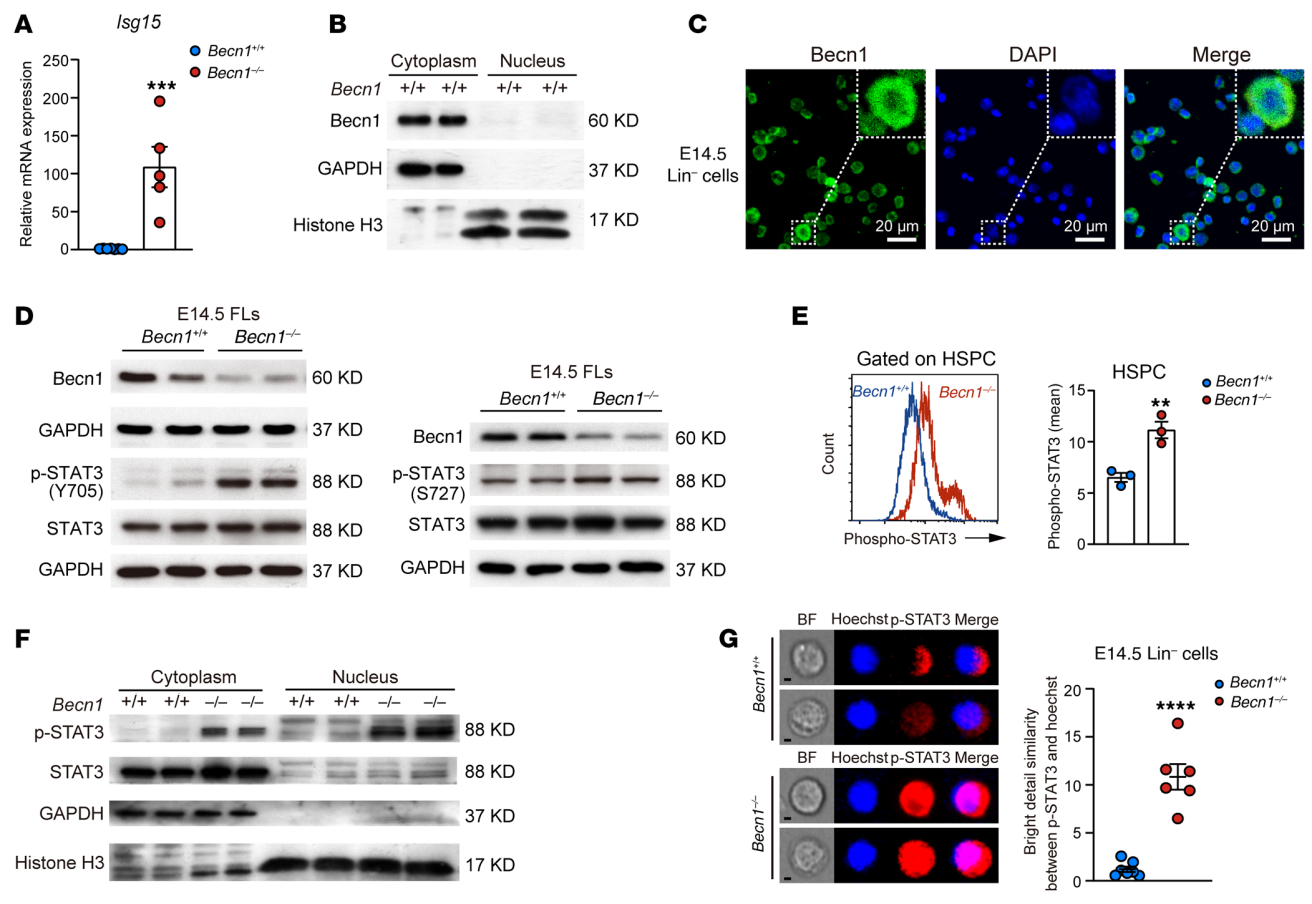
*Becn1* deletion leads to upregulation of *Isg15* transcription and phosphorylation of STAT3. (**A**) Real-time qPCR analysis of *Isg15* expression in E14.5 fetal livers (*n* = 5–7). (**B**) Becn1 localization analysis by Western blotting of nucleus-cytosol extracts from E14.5 fetal livers. (**C**) Representative images of Becn1 localization in Lin^–^ cells at E14.5, as determined by immunofluorescence analysis. Scale bars: 20 μm. (**D**) Western blotting for STAT3 activation in E14.5 FLCs. (**E**) Flow cytometric analysis of p-STAT3 levels in HSPCs from E14.5 fetal livers (*n* = 3). Representative pictures of flow cytometric analysis (left) and analysis of flow cytometric results (right) are shown. (**F**) Analysis of the subcellular locations of p-STAT3 and STAT3 in E14.5 FLCs. (**G**) Flow cytometric analysis of the subcellular location of p-STAT3 in E14.5 Lin^–^ FLCs (*n* = 6–7). BF, bright field. Scale bars: 2.5 μm. **P < 0.01; ***P < 0.001; ****P < 0.0001. Unpaired 2-tailed Student’s *t* test Data represent the mean ± SEM.

Flow cytometric analysis revealed an increase in ISG15 in *Becn1*-deleted fetal liver hematopoietic cells. To understand how Becn1 regulates the upregulation of ISG15, we performed real-time qPCR and observed an approximately 100-fold increase in the transcription of *Isg15* in E14.5 FLCs (Figure 6A), indicating that loss of Becn1 causes dramatic transcriptional activation of *Isg15*. To determine whether Becn1 may directly promote the transcription of *Isg15*, we examined the subcellular localization of Becn1 in FLCs via nuclear-cytoplasmic separation and immunofluorescence. The results revealed that Becn1 is located in the cytoplasm (Figure 6, B and C), excluding the possibility that Becn1 directly regulates *Isg15* transcription in the nucleus of FLCs.

To screen tentative factors that may act as transcription factors for ISG15 expression, we searched the GeneCards database, which discloses CREB, δCREB, MyoD, P53, and STAT3 as putative transcription factors of *ISG15* in humans. The activity of STAT3 is controlled mainly by its phosphorylation at tyrosine 705 (Y705) and/or serine 727 (S727), which leads to its subsequent translocation to the nucleus, where phosphorylated STAT3 (p-STAT3) regulates the transcriptional activation of its downstream genes (39–41). STAT3 has a critical role in inflammation and immunity (42, 43). In our present study, the innate immune response and inflammatory signaling were increased, and cytokines were increased in *Becn1^–/–^* fetal hematopoietic cells (Figures 4 and 5), which suggests that a cytokine storm develops after the loss of Becn1.

We therefore postulated that STAT3 is able to transcriptionally activate ISG15 in FLCs. To test this hypothesis, we first performed Western blotting with antibodies against p-STAT3 to detect STAT3 activity. In E14.5 *Becn1^–/–^* fetal livers, STAT3 was activated very significantly at Y705 and slightly at S727 (Figure 6D). Analysis of the flow cytometric data further revealed the activation of STAT3 at Y705 in E14.5 *Becn1^–/–^* HSPCs (Figure 6E). As expected, p-STAT3 (Y705) entered the nucleus in *Becn1*-deleted E14.5 FLCs (Figure 6F). With E14.5 Lin^–^ liver cells, which represent fetal hematopoietic cells, p-STAT3 (Y705) also increased in localization in the nucleus, as shown by image flow cytometry analysis (Figure 6G). Together, these results suggest that STAT3 is activated and translocated to the nucleus when *Becn1* is deleted in fetal liver hematopoietic cells.

**Figure 7:**
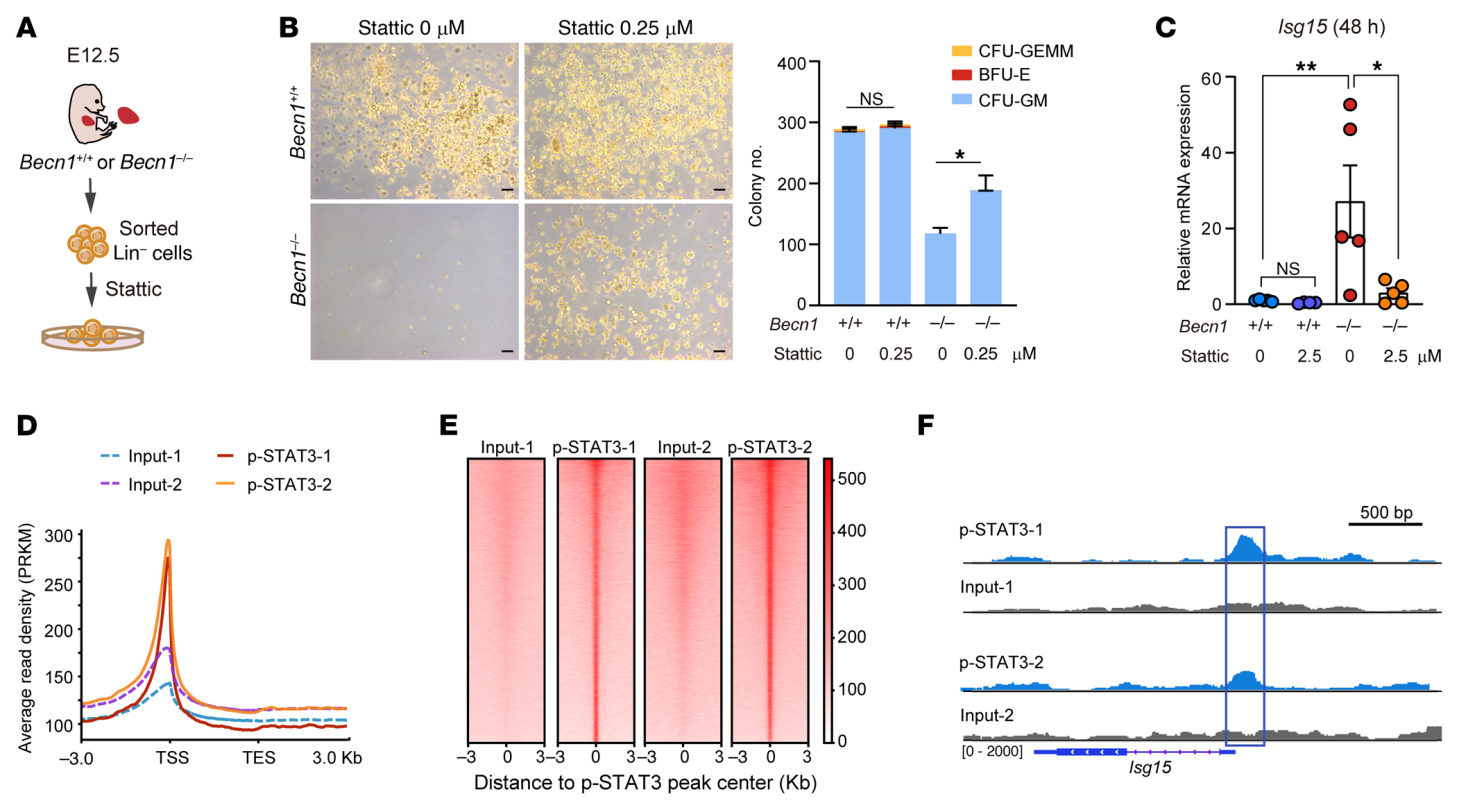
Inhibition of STAT3 activity rescues colony-forming ability and reduces *Isg15* transcription in *Becn1*-deleted fetal liver hematopoietic cells, and *Becn1* deletion results in p-STAT3 binding to the *Isg15* gene. (**A**) Schematic plan for CFU assay of STAT3 inhibitor–treated Lin^–^ cells from E12.5 FLCs. (**B**) Pharmacological inhibition of STAT3 reversed the colony formation capacity of *Becn1^–/–^* liver hematopoietic cells. Left: Representative images. Right: Statistical results of colonies from E12.5 fetal liver Lin^–^ cells treated with a STAT3 inhibitor (*n* = 4). Scale bars: 100 μm. (**C**) Real-time qPCR analysis of *ISG15* expression in E12.5 Lin^–^ cells after STAT3 inhibitor treatment. *n* = 4–5. (**D**) Profile plots of p-STAT3 enrichment at the transcription start site (TSS) region based on ChIP-Seq of E14.5 *Becn1^–/–^* fetal livers (*n* = 2). (**E**) Peak center read density heatmap of ChIP-Seq signals for the binding of p-STAT3 in E14.5 FLCs. (**F**) p-STAT3 interacted with the *Isg15* gene according to the Integrative Genomics Viewer (IGV). *P < 0.05; **P < 0.01. Unpaired 2-tailed Student’s *t* test (**B**); 1-way ANOVA (Tukey’s multiple-comparison test) (**C**). Data represent the mean ± SEM.

To establish a functional link between STAT3 and ISG15 in the context of *Becn1* deletion in fetal hematopoietic organs, we blunted the activity of STAT3 in *Becn1^–/–^* E12.5 liver Lin^–^ cells with Stattic, a compound that can effectively inhibit STAT3 activation and nuclear translocation (Figure 7A). Colony formation assays revealed that inhibition of STAT3 activity could partially rescue the function of fetal hematopoietic progenitor cells (Lin^–^) impaired by *Becn1* deletion (Figure 7B), and *Isg15* transcription was significantly decreased in E12.5 liver Lin^–^ cells after 48 hours of inhibitor treatment (Figure 7C), suggesting that activated STAT3 may promote the transcriptional activation of ISG15.

Next, to determine whether STAT3 can possibly act as a transcription factor of *Isg15*, we performed ChIP sequencing (ChIP-Seq) on E14.5 *Becn1^–/–^* FLCs. The ChIP-Seq results revealed that p-STAT3 binding was enriched at gene transcription start sites (Figure 7D), further suggesting that p-STAT3, as a transcription regulator, may bind to its downstream target genes, such as *Isg15*. In accordance with the above observations, a heatmap of the peak center read density of the ChIP-Seq signals suggested enriched binding of p-STAT3 to the DNA sequences of its downstream targets (Figure 7E). We analyzed the peaks from 2 independent ChIP-Seq events via a Venn diagram, which displayed 10,141 overlapping peaks (Supplemental Figure 10A). A pie plot further revealed that the overlapping peaks from 2 independent ChIP-Seq datasets were distributed mainly in the promoter region (Supplemental Figure 10B). Importantly, we observed that p-STAT3 could interact with the *Isg15* gene via the Integrative Genomics Viewer (Figure 7F). Overall, our data suggest that loss of *Becn1* can lead to activation of STAT3 and subsequent translocation of p-STAT3 to the nucleus, where p-STAT3 promotes the transcription of *Isg15*, thus triggering downstream inflammation cascades and leading to fetal hematopoietic failure.

### Becn1 binds to STAT3 to suppress STAT3/ISG15 activation.

**Figure 8:**
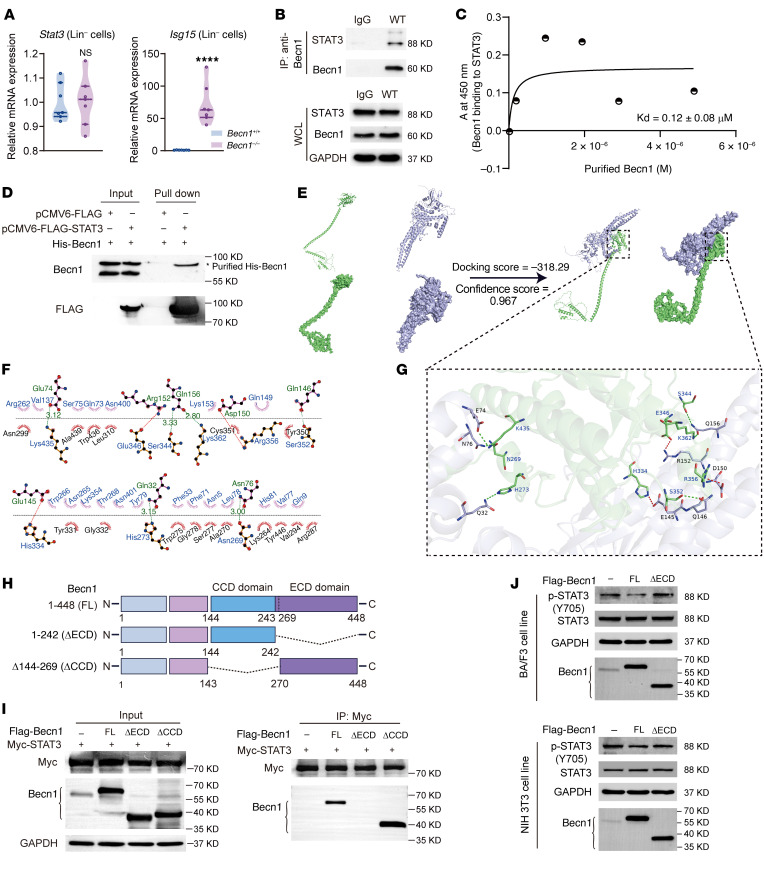
Becn1 binds to STAT3 via the Becn1 ECD domain to suppress STAT3/ISG15 signaling. (**A**) Real-time qPCR analysis of *STAT3* and *ISG15* expression in Lin^–^ FLCs at E14.5 (*n* = 7). (**B**) E14.5 FLCs were subjected to immunoprecipitation with an anti-Becn1 antibody. The immune complexes and whole-cell lysates (WCLs) were analyzed by immunoblotting with anti-STAT3 and -Becn1 antibodies. (**C**) Analysis of Becn1 binding to STAT3 via ELISA. (**D**) Becn1-STAT3 interaction assay by FLAG affinity pull-down. Purified His-tagged mouse Becn1 protein was expressed in *E*. *coli* cells, and the pCMV6-FLAG-STAT3 or pCMV6-FLAG vector was transfected into 293T cells to express FLAG-STAT3 and FLAG. (**E**) The structure and protein-protein docking model of mouse Becn1 (green) and mouse STAT3 (purple). (**F**) 2D interaction patterns between mouse Becn1 and mouse STAT3. The tooth-like amino acids represent hydrophobic interactions, the green dashed lines represent hydrogen bonding, and the red dashed lines represent salt bridge interactions. (**G**) 3D interaction patterns between mouse Becn1 and mouse STAT3. (**H**) Schematic diagram of the construction of Becn1 mutants. (**I**) Identification of the binding domain of Becn1 to STAT3 via co-IP. Myc-STAT3 and FLAG-Becn1 (FL) or Becn1 mutant constructs were expressed in 293T cells. Total cell lysates were immunoprecipitated via c-Myc magnetic beads, followed by immunoblotting with the indicated antibodies. (**J**) Western blotting analysis of p-STAT3, STAT3, and Becn1 levels in BA/F3 or NIH 3T3 cells. FLAG-Becn1 (FL) or Becn1 mutant (ΔECD) constructs were expressed in BA/F3 or NIH 3T3 cells. ****P < 0.0001. Unpaired 2-tailed Student’s *t* test. Data represent the mean ± SEM.

In mice, when Becn1 is intact in wild-type embryos, STAT3 is virtually not phosphorylated in the cytoplasmic compartment. Once STAT3 is phosphorylated as a result of *Becn1* deletion, its translocation occurs, which in turn promotes *Isg15* transcription, followed by extensive ISGylation of its downstream targets to trigger inflammatory cytokine production (Figures 4–7). The question now is how cytoplasmic STAT3 is controlled in fetal hematopoietic organs under physiological conditions. We speculated that Becn1 may regulate STAT3 in the cytoplasm since the loss of Becn1 leads to the activation of STAT3. Given that the absence of Becn1 caused the upregulation of ISG15 at the transcriptional, translational, and posttranslational levels in liver cells (Figures 4–6) and Lin^–^ liver cells (Figure 8A, right) but did not increase STAT3 at the mRNA or protein level (Figure 6D and Figure 8A, left), we further speculated that Becn1 may regulate STAT3 in the posttranslational state, such as via phosphorylation or physical interaction. To this end, we lysed E14.5 FLCs and conducted immunoprecipitation with Becn1 as bait and STAT3 as prey. Western blotting with an anti-Becn1 antibody revealed that Becn1 and STAT3 were in the same protein complex (Figure 8B). To confirm whether Becn1 directly interacts with STAT3, we quantified the binding affinity via ELISA with a documented protocol (44), and the results revealed that the binding affinity for the Becn1-STAT3 interaction was estimated at a dissociation constant of approximately 0.12 ± 0.08 μM (Figure 8C), suggesting robust binding affinity (45). Furthermore, pull-down assays with ectopically overexpressed and purified Becn1 and STAT3 proteins indicated that Becn1 directly bound to STAT3 (Figure 8D).

To predict the binding region and interaction mode between Becn1 and STAT3, we used HDOCK (46, 47) for protein-protein docking. We obtained complete 3-dimensional structural models of the mouse proteins Becn1 and STAT3 by using AlphaFold2 (48) (Figure 8E). After protein-protein docking, we chose the structure with the highest docking score as the benchmark result for subsequent interaction analysis. According to the docking model of the 2 proteins, the evolutionarily conserved domain (ECD) of Becn1 is bound to the N-terminal domain (NTD) and coiled-coil domain (CCD) of STAT3, resulting in a relatively stable interaction between the 2 proteins (Figure 8E). Analysis of the interaction patterns between Becn1 and STAT3 in the binding region revealed a total of 6 hydrogen bond interactions and 3 salt bridge interactions between the 2 proteins. The main amino acids involved in forming hydrogen bonds in Becn1 were Asn269, His273, His334, Ser344, Glu346, Ser352, Arg356, Lys362, and Lys435, whereas the main amino acids in STAT3 participating in hydrogen bonding were Gln32, Glu74, Asn76, Glu145, Gln146, Asp150, Arg152, and Gln156. Furthermore, Becn1 was projected to form numerous hydrophobic interactions with STAT3, which presumably enhances the binding between Becn1 and STAT3 (Figure 8, F and G). Therefore, the results of protein-protein docking support a direct and robust interaction between Becn1 and STAT3, suggesting that Becn1 tethers STAT3 to prevent its activation and nuclear translocation.

To validate the results of the molecular docking prediction, we constructed stable 293T cells overexpressing full-length Becn1 and Becn1 mutant proteins by removing either of the 2 structural domains of Becn1, a central CCD domain (amino acids 144–269) or an ECD domain (amino acids 243–448) (Figure 8H) (27). The plasmid expressing Myc-tagged STAT3 was then transiently expressed in these cells. We next examined the interaction between Becn1 (full length, ΔECD or ΔCCD) and STAT3 via co-IP. The results showed that Becn1 lacking the ECD domain was unable to interact with STAT3, whereas full-length Becn1 and Becn1 lacking the CCD domain could interact with STAT3, indicating that the ECD of Becn1 is the binding domain to STAT3 (Figure 8I).

To test whether the ECD domain of Becn1 identified by an in vitro biochemical assay using exogenous expressed Becn1 and STAT3 functions in binding to STAT3 in cells, we conducted an in vitro cellular assay with BA/F3 cells (a mouse pro–B cell line) and NIH 3T3 cells (an embryonic mouse fibroblast cell line) by overexpressing FLAG-Becn1 (either full-length or ECD-deleted Becn1). The results showed that the overexpression of full-length Becn1 reduced p-STAT3, and the loss of the ECD domain in Becn1 increased p-STAT3 levels, which was more pronounced in BA/F3 cells than in NIH 3T3 cells (Figure 8J), suggesting that Becn1 preferentially binds to STAT3 in hematopoietic-lineage cells. These findings, together with the above results (Figures 4–7), indicate that Becn1, via its ECD domain, binds STAT3 to prevent ISG15 activation and cytokine storms in fetal hematopoietic organs.

Additionally, HDOCK prediction suggested that the ECD domain of human Becn1 binds to the NTD domain, CCD domain, and DNA-binding domain (DBD) of human STAT3 and revealed a total of 6 hydrogen bond interactions and 4 salt bridge interactions between the 2 proteins. The main amino acids involved in forming hydrogen bonds in human Becn1 were Ser279, Gly334, Asn335, His336, Ser346, Glu348, Arg358, Lys364, and Lys437, whereas the main amino acids in human STAT3 participating in hydrogen bonding were Asn5, Glu74, Glu145, Asp150, Arg152, Gln156, Asn265, and Asn401 (Supplemental Figure 11), and the prediction excluded a direct interaction between Becn1 and STAT1/2 (Supplemental Figure 12). These results suggest that the inhibition of STAT3/ISG15 activation may be a conserved mechanism in which Becn1 restrains STAT3 to secure fetal hematopoiesis and survival in mammalian species.

### Becn1-mediated inhibition of the STAT3/ISG15 axis does not rely on autophagy.

**Figure 9:**
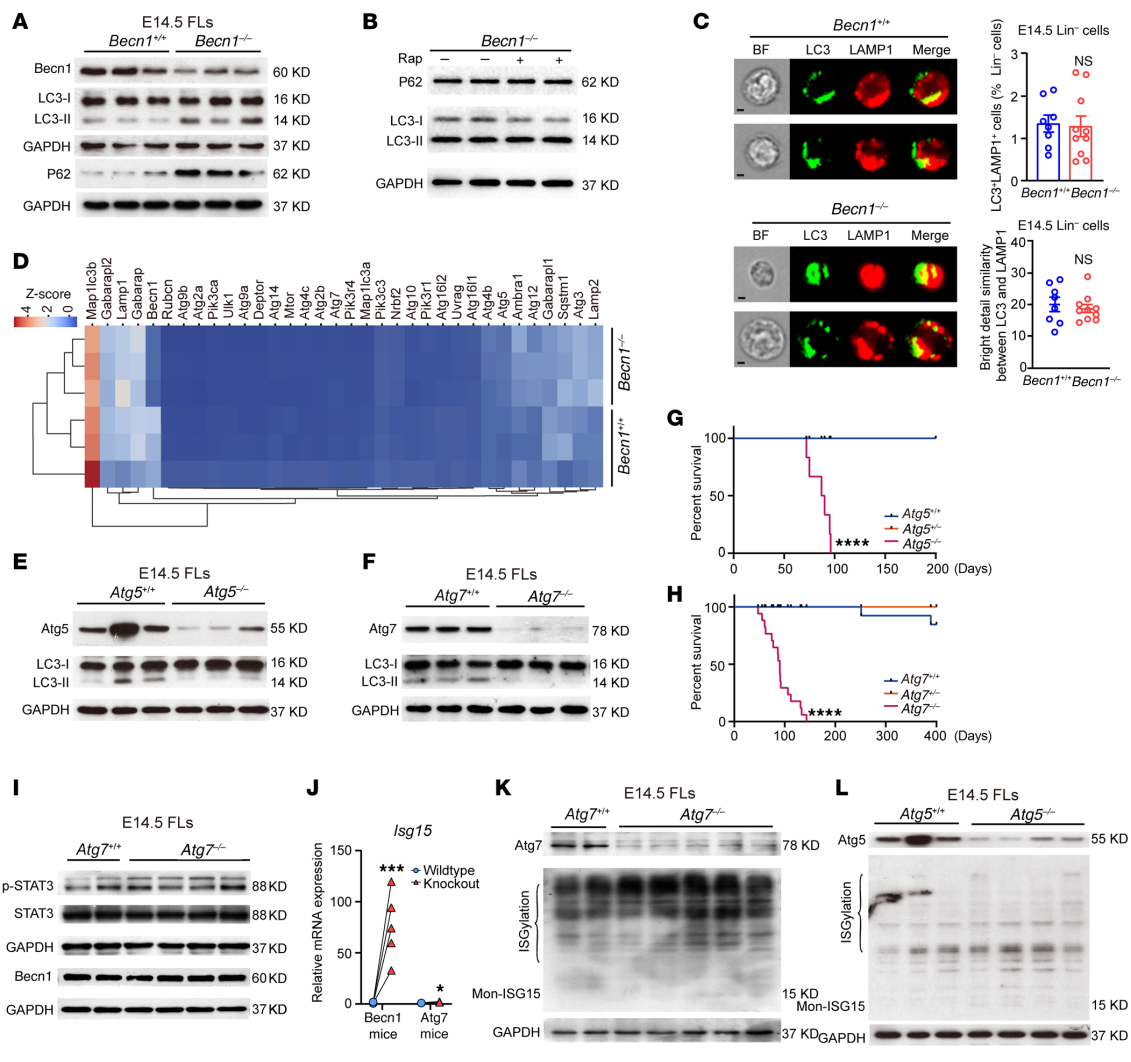
Deletion of *Becn1* impairs autophagy, but STAT3/ISG15 signaling is not activated in *Atg5/7*-deleted fetal liver hematopoietic cells. (**A** and **B**) Western blotting analysis of LC3 and P62 levels in E14.5 *Becn1^+/+^* and *Becn1^–/–^* FLCs with or without 100 nmol/L rapamycin. (**C**) Autolysosome formation was measured by image flow cytometry for double staining of LC3 and LAMP1 with fetal liver Lin^–^ cells. Autolysosome formation was represented by the colocalization of LC3 and the lysosomal marker LAMP1. Left: Representative flow images. Right: Results of flow image statistical analysis. Scale bars: 2.5 μm. *n* = 8–10. (**D**) Heatmap of autophagy-related genes from the E14.5 HSPC transcriptome. (**E** and **F**) Western blotting analysis of LC3 in E14.5 *Atg5/7^+/+^* and *Atg5/7^–/–^* FLCs. (**G** and **H**) Survival curves of the *Atg5/7^+/+^*, *Atg5/7^+/–^*, and *Atg5/7^–/–^* mice. Log-rank test. *n* = 3–17. (**I**) p-STAT3, STAT3, and Becn1 levels were detected via Western blotting in E14.5 *Atg7^+/+^* and *Atg7^–/–^* FLCs. (**J**) Real-time qPCR analysis of *Isg15* expression in E14.5 fetal livers from *Becn1^fl/fl^* Vav-iCre mice (*n* = 5) and *Atg7^fl/fl^* Vav-iCre mice (*n* = 3). (**K** and **L**) Western blotting analysis of ISG15 levels in E14.5 fetal livers from *Atg7^fl/fl^* Vav-iCre mice or from *Atg5^fl/fl^* Vav-iCre mice. *P < 0.05; ***P < 0.001; ****P < 0.0001. Unpaired 2-tailed Student’s *t* test. Data represent the mean ± SEM.

To examine whether autophagy in fetal hematopoietic organs is influenced by conditional *Becn1* deletion, we measured the expression of autophagic markers, and the results revealed in vivo accumulation of the LC3-II and P62 proteins in *Becn1^–/–^* FLCs (Figure 9A). Treatment with rapamycin, an autophagy inducer, did not enhance LC3 transformation in *Becn1^–/–^* FLCs (Figure 9B), suggesting that functional autophagy was likely impaired by *Becn1* deletion. We also compared the protein levels of autolysosomal markers between *Becn1^+/+^* and *Becn1^–/–^* mice, which revealed no accumulation in *Becn1^–/–^* FLCs (Figure 9C), apparently owing to the absence of Becn1 deletion-caused inability to form autolysosomes, since Becn1 is indispensable for the membrane trafficking essential for the formation of autolysosomes (27). In addition, brief examination via transcriptomic profiling did not reveal apparent changes in the expression of autophagy-related genes after *Becn1* deletion (Figure 9D), excluding compensatory expression of other autophagy-related genes, which may lead to compensatory or alternative autophagy. Taken together, these data suggest that autophagy is impaired in *Becn1*-deleted fetal hematopoietic organs.

To investigate the impact of autophagy on the Becn1/STAT3/ISG15 axis, we used 2 autophagy-null mouse models (*Atg7^–/–^* and *Atg5^–/–^*, exclusively in the hematopoietic system) (Figure 9, E and F) (49, 50) to test whether the Becn1/STAT3/ISG15 axis relies on autophagy. Unlike embryonic lethality caused by deletion of *Becn1* (Figure 1), deletion of *Atg5* or *Atg7* in hematopoietic cells did not cause fetal mortality, and these mice survived several months after birth (Figure 9, G and H). Furthermore, Becn1, STAT3, and p-STAT3 protein levels were not altered in the *Atg7*-deleted mice (Figure 9I). ISG15 transcription was virtually unchanged in the *Atg7*-deleted mice compared with the *Becn1*-deleted mice (Figure 9J). Similarly, ISGylation did not occur in the *Atg5-* or *Atg7*-deleted mice (Figure 9, K and L), which is in sharp contrast to the outcomes in the *Becn1*-deleted mice (Figure 5B and Figure 6A). This genetic evidence suggests that the Becn1/STAT3/ISG15 pathway operates independently of autophagy.

Finally, to investigate the pathophysiological significance of Becn1-mediated ISG15 signaling in fetal hematopoiesis and development, we evaluated whether Becn1 is involved in governing maternal immune activation (MIA), a major immune risk to embryos during pregnancy (51–53). We generated an MIA mouse model via the injection of pIpC into pregnant mice, which revealed that at E14.5, Becn1 expression in FLCs was reduced (Supplemental Figure 13, A and B), and concurrently, STAT3 was phosphorylated/activated (Supplemental Figure 13C), along with elevated transcriptional levels of ISG15 and the cytokine IFN-α (Supplemental Figure 13, D and E). As a result, HSPCs of fetal liver abnormally proliferated (Supplemental Figure 13F), indicating that hematopoietic function was impaired by MIA. Notably, the reduction in Becn1 levels and the activation of STAT3/ISG15 were coupled in MIA mice, mimicking the activation of this axis by *Becn1* deletion in fetal hematopoietic organs. These findings suggest that the fetal Becn1/STAT3/ISG15 axis may function to prevent MIA during pregnancy.

## Discussion

In this study, we present an examination of Becn1 in fetal hematopoiesis and find that Becn1, via its ECD domain, directly interacts with STAT3 to block the activation and nuclear translocation of STAT3, thus preventing its upregulation of the transcription and activation of ISG15, the amplifier of inflammation, thereby controlling the production of inflammatory cytokines during fetal hematopoiesis and development.

The host response to interferon (IFN) drives rapid immune defense against invading pathogens, primarily via the production of inflammatory cytokines. Upon the detection of pathogens such as viruses, innate sensor proteins initiate downstream antiviral signaling pathways, leading to type I and type III IFN gene expression (10, 11, 13). In the ISG15-conjugated pathway, ISG15 exerts antiviral effects by functioning as a posttranslational modifier of host and viral proteins, known as ISGylation, which involves a stepwise action of an E1 enzyme that activates ISG15, an E2-conjugating enzyme, and an E3 ligase that catalyzes the final step of ISG15 conjugation to the substrate protein (14, 54–56). Covalent conjugation of ISG15 to lysine residues in target proteins promotes cytokine production and many other cellular cascades, including translation, trafficking, and DNA damage responses (14, 57). In parallel, unconjugated ISG15 can be secreted into the extracellular milieu (58), directly acting as a cytokine to induce an immune response (13, 59–61). Recent studies have indicated that SARS-CoV-2 infection of human macrophages derived from induced pluripotent stem cells can reinforce ISG15 secretion via the deISGylating activity of the viral papain-like protease, which in turn contributes to aberrant macrophage activation and excessive production of proinflammatory cytokines; therefore, extracellular non-conjugated ISG15 can act as a cytokine to exacerbate inflammation in severe COVID-19 (15, 16, 62).

Although ISG15 and ISGylation have been shown to function in normal tissue differentiation, especially in placental and fetal development (63, 64), ISG15 is minimally expressed under physiological conditions, suggesting tight control of ISG15 during embryonic development. The expression of ISG15 and its conjugation enzymes is positively regulated by the interaction between IFNs and the IFN-sensitive responsive elements of the *ISG15* promoter (13, 14). Constitutive overexpression of ISG15 represents a pathological condition that manifests as increased ISG15 in cancers (65). Therefore, decreasing the expression and activation of ISG15 and its conjugation pathway may invoke severe innate immune responses and pathological outcomes. However, how this potential risk is rigorously controlled has not been resolved thus far.

In our study, we found significantly increased ISG15 and high production of proinflammatory cytokines in *Becn1^–/–^* HSPCs in the fetal liver, a major hematopoietic organ during embryonic development. We achieved partial but significant rescue of HSPC function and mitigation of hyperinflammation by *ISG15* knockdown in *Becn1^–/–^* mice, suggesting that Becn1 regulates fetal hematopoiesis and the inflammatory response by suppressing ISG15. Our results thus suggest that Becn1 is a key upstream regulator that controls innate immune and inflammatory responses in fetal hematopoiesis.

Inflammatory cytokines and other inflammatory compounds can damage adult hematopoietic stem cells, causing abnormal proliferation and dysfunction of HSCs (66–68). On the other hand, adult HSCs and their downstream progenitors have also been reported to produce a series of proinflammatory cytokines by directly activating Toll-like receptors (69). Several groups have reported that essential inflammatory signals (IFN-α and IFN-γ) and RIG-I receptors regulate the development of fetal HSCs and progenitors (70–74). These reports, together with our findings, suggest that cytokines at low levels are possibly needed by hematopoietic cells, but overproduction of inflammatory cytokines is deleterious. Our results revealed that *Becn1^–/–^* fetal HSPCs experienced faster proliferation, cell cycle progression, and defective function, similar to the changes in adult HSCs stimulated by strong inflammatory signals and pathogens. These studies indicate that HSPCs in the fetal liver and bone marrow are sensitive to inflammatory signals, indicating the need for embryos to develop a mechanism that can effectively address unexpected breaks in deleterious cytokine storms in hematopoietic organs. In addition to our finding in the genetic program of control of inflammatory insults by Becn1, such risk control can also be achieved at the epigenetic level. For example, the m^6^A writer METTL3 in the murine fetal liver protects hematopoietic development by controlling the innate immune response via the suppression of endogenous dsRNA formation (75). Together, these works suggest that embryos have both genetic and epigenetic mechanisms to tightly secure fetal hematopoiesis from inflammatory attack.

Unlike the myeloid-biased differentiation of HSCs driven by chronic inflammation in aging populations, our study revealed that the loss of Becn1 leads to lymphoid-biased and inflammatory cell–biased differentiation and deleterious cytokine storms in fetal hematopoietic organs. These findings suggest that the effect of the decontrol of cytokine production on fetal hematopoiesis is different from that of chronic inflammation on adult hematopoiesis. Furthermore, contrary to the involvement of STAT1/2 in the activation of ISG15 triggered by IFNs (13, 14), our results indicate that Becn1 regulation of ISG15 involves STAT3 and that activated STAT3 can promote the transcription of *ISG15* in fetal liver hematopoietic cells (Figures 6 and 7). Specifically, Becn1 suppresses ISG15 activation by tethering STAT3 via the Becn1 ECD domain to form the Becn1-STAT3 complex, thus hindering the signaling cascade, including the activation and translocation of STAT3 to the nucleus and the subsequent promotion of *ISG15* transcription, which ultimately leads to hyperinflammation. Therefore, a key step in controlling ISG15 activation is to arrest STAT3 via Becn1, and Becn1 prevents ISG15-mediated cytokine storms by inhibiting STAT3/ISG15 activation and signaling to protect normal hematopoiesis during embryonic development.

Although several autophagy-related proteins are implicated in immune modulation in an autophagy-independent manner (76–79), in terms of Becn1, an in vivo study on the relationship between Becn1 and immunity revealed that heterozygous deletion of *Becn1* causes neuroinflammation due to autophagy impairment (80) and that myeloid loss of Becn1 results in spontaneous immune activation via IFN-γ–dependent signaling and resistance to *Listeria monocytogenes* infection (34). A more recent study reported that Becn1 in myeloid cells protects mice against fatal TNF- and LPS-induced cytokine storm syndrome (81). However, the actions of Becn1 in these cases are dependent on the integrity of autophagy; moreover, these actions are stimulated by exogenous factors such as LPS, TNF-α, or *Listeria*, and the survival of the mice with myeloid loss of Becn1 was not affected in the absence of exogenous stimulation. Therefore, these reports suggest that Becn1 is involved in mediating inflammation via extrinsic triggers, which are all dependent on the autophagy machinery.

In our study, however, we observed that the loss of *Becn1*, but not exogenous stimulation, could result in overactivated innate immune and inflammatory signaling in the fetal hematopoietic liver, particularly in its HSPCs, manifested by significantly increased levels of proinflammatory factors (such as TNF-α, IL-6, and IFN-γ) in *Becn1^–/–^* fetal hematopoietic cells. We also found that the failure of fetal hematopoiesis did not depend on IFN-γ signaling and that the increase in IFN-γ due to the loss of Becn1 is not a major cause of hyperinflammation in *Becn1^–/–^* mice, since the deletion of IFN-γ failed to rescue the homeostasis of the fetal HSC pool and hematopoietic potential in *Becn1^–/–^* mice (Supplemental Figure 14). This finding excludes the possibility that IFN-γ is a major downstream effector of Becn1 in the control of STAT3/ISG15 signaling and prevention of fetal inflammation. Thus, our study revealed that Becn1 prevents hyperinflammation to protect embryos from intrinsic cytokine storms during fetal hematopoiesis by suppressing STAT3/ISG15 activation. In addition, the MIA model assay suggested that Becn1 control of the STAT3/ISG15 axis is also important. Therefore, Becn1-mediated suppression of STAT3/ISG15 signaling is implicated in counteracting both intrinsic and extrinsic inflammatory insults. The molecular mechanism controlling fetal inflammation identified in the present study is different from that of the well-established IFN/ISG15 axis in response to virus infection (13, 14, 34). Our findings are also different from a recent study showing that Becn1 modulates HSCs by targeting caspase-3/GSDME–mediated pyroptosis (82). This discrepancy may stem from different efficiencies in inactivating Becn1 due to the use of different gene knockout strategies.

The limitations of our study in part lie in the constitutive but not inducible deletion of *Becn1* in the mouse model used. However, the currently available solution to use *Mx1*-Cre for inducible deletion of *Becn1* in the hematopoietic system requires poly(I:C) induction. Exogenous induction with poly(I:C) masks the bona fide influence of Becn1 on the innate immune response, because this compound itself induces an antiviral immune response (83), thus prohibiting our consideration of *Mx1*-Cre–mediated deletion of *Becn1* in mice. Instead, we chose *Vav*-iCre mice to constitutively delete the *Becn1* gene, because *Vav* starts expression at E11.5 (84), which is approximately early enough to cover fetal liver hematopoiesis and thus allows us to analyze the effects of *Becn1* deletion on fetal liver hematopoiesis. Nevertheless, the *Vav*-iCre model has limitations in investigating the role of Becn1 in HSCs between E10.5 and E11.5, because during this time window, HSCs emerge in the aorta-gonad-mesonephros region and then migrate to the fetal liver to begin fetal liver hematopoiesis.

In summary, our study provides an answer to a long-standing question concerning the control of inflammation during fetal hematopoiesis, and Becn1 prevents hyperinflammation by suppressing STAT3/ISG15 activation. Therefore, Becn1 may be developed as a therapeutic target for preventing or mitigating inflammatory diseases during embryonic development.

## Methods

### Sex as a biological variable.

Our study examined male and female animals, and similar findings are reported for both sexes.

### Mice.

*Becn1^fl/fl^* mice were generated via gene targeting in this study, as described in Figure 1A. Vav-iCre transgenic mice were purchased from The Jackson Laboratory. *Becn1*-conditional-KO mice were generated by crossing of *Becn1*-floxed mice with Vav-iCre mice. *Atg5^fl/fl^* and *Atg7^fl/fl^* mice were provided by Noboru Mizushima (The University of Tokyo, Tokyo, Japan) (49) and Masaaki Komatsu (Juntendo University, Tokyo, Japan) (50). All the mice were bred on a C57BL/6 genetic background.

### Cell lines.

293T and NIH 3T3 cells were purchased from ATCC. The murine BA/F3 cell line was a gift from Terry Fox Laboratory, British Columbia Cancer Research Institute, Vancouver, Canada.

### Chemicals and biological reagents.

Cells were treated with 0.25 μM Stattic for 7–9 days in the CFU assay or with 2.5 μM Stattic for 48 hours to inhibit STAT3 phosphorylation. To establish the MIA model, poly(I:C) (20 mg/kg) was intraperitoneally injected into pregnant mice.

### Transplantation assay.

A total of 5 × 10^5^ FLCs were injected into lethally irradiated (9 Gy) CD45.1^+^ recipients through the tail vein. The recipient mice were monitored for up to 30 days after transplantation, and the number of dead mice was recorded every day.

A total of 5 × 10^5^ FLCs mixed with 5 × 10^5^ competitive bone marrow cells were injected into lethally irradiated (9 Gy) CD45.1^+^ recipients through the tail vein.

### RNA sequencing and proteomic analysis.

HSPCs were sorted from E14.5 FLCs. Total RNA was extracted from sorted HSPCs using TRIzol according to the manufacturer’s instructions and processed in a standard mRNA library construction pipeline. The final product was sequenced in paired-end 100 bp mode using the BGISEQ500 platform (BGI). After data quality control, differential expression gene identification was performed based on |log_2_(fold change)| ≥ 1 and a *q* value less than 0.05 as the criteria for statistically significant differences. The peptide samples from fetal livers were subjected to liquid chromatography–tandem mass spectrometry via a Fusion Lumos Tribrid mass spectrometer (Thermo Fisher Scientific) equipped with an UltiMate 3000 UHPLC System (Thermo Fisher Scientific). Mass spectrometry data were acquired in data-dependent acquisition mode. The raw data were processed by MaxQuant (version 1.5.3.0) (https://www.maxquant.org) for feature detection. |log_2_(fold change)| ≥ log_2_(1.5) and *P* value less than 0.05 indicated significant differentially expressed proteins. Overrepresentation analysis was carried out using clusterProfiler (85). We constructed volcano plots and heatmaps via the Python library bioinfokit (86). GSEA was conducted on pre-ranked gene lists with log_2_(fold change) as the ranking metric, and GO terms or KEGG pathways were used as gene sets. The sequences for all the primers used in the real-time qPCR assays are shown in Supplemental Table 2.

### Intracellular flow cytometric analysis.

FLCs were stained with various antibodies, fixed with paraformaldehyde, permeabilized with saponin, subsequently stained with ISG15, p-Tyr705-STAT3, or Hoechst 33342 antibodies, and then analyzed via Beckman Coulter Gallios or Imaging Flow Cytometer (Amnis, Merck Millipore). The information for all antibodies used in the staining is provided in Supplemental Table 1.

### Isg15 knockdown and rescue experiments.

Control lentiviral LV3 vectors and LV3 vectors containing shRNA for mouse *Isg15* were purchased from GenePharma. The 4 shRNA sequences of mouse *Isg15* were shRNA-1 (5′-GCAGATTGCCCAGAAGATTGG-3′), shRNA-2 (5′-GCACAGTGATGCTAGTGGTAC-3′), shRNA-3 (5′-GCAGACTGTAGACACGCTTAA-3′), and shRNA-4 (5′-GCACAGTGATCAAGCATTTGC-3′). Viral particles were concentrated by ultracentrifugation. To achieve stable FLCs with *Isg15* knockdown, Lin^–^ cells were maintained in IMDM supplemented with IL-3 (20 ng/mL), IL-6 (20 ng/mL), thrombopoietin (TPO) (50 ng/mL), Flt3L (50 ng/mL), or SCF (100 ng/mL). *Isg15* shRNA-2 (5′-GCACAGTGATGCTAGTGGTAC-3′) was used for screening (Supplemental Figure 8B). Lentiviral medium was added to infect Lin^–^ cells in a 96-well plate coated with fibronectin. After transduction, the GFP^+^ cells were sorted and plated with MethoCult (STEMCELL Technologies). Colonies were counted and morphologically classified 6 days after plating.

### ChIP-Seq library preparation and data analysis.

Cells were cross-linked with formaldehyde and quenched with glycine. The chromatin fragments were precleared and then immunoprecipitated with protein A+G magnetic beads coupled with anti–Tyr705-STAT3 antibodies. After reverse cross-linking, ChIP and input DNA fragments were end-repaired and A-tailed using the NEBNext End Repair/dA-Tailing Module (E7442, New England Biolabs) followed by adaptor ligation with the NEBNext Ultra Ligation Module (E7445, New England Biolabs). The DNA libraries were amplified and sequenced using an Illumina NovaSeq 6000. Raw reads were filtered to obtain high-quality clean reads using Cutadapt (v1.9.1) (87) and Trimmomatic (v0.35) (88). The clean reads were mapped to the mouse genome (assembly GRCm38) using Bowtie2 (v2.2.6) software (89). Peak detection was performed using Model-based Analysis of ChIP-Seq (MACS; v2.1.1) (90). Annotation of peak sites to gene features was performed using the ChIPseeker R package (91).

### Protein-protein interaction assays.

For endogenous co-IP, cell supernatants were added to protein A/G agarose after incubation with anti-Becn1. The bound proteins were boiled in loading buffer for further analysis. For exogenous co-IP, anti–c-Myc magnetic beads were added to equal amounts of total protein and incubated at 4°C overnight. Beads were washed and then boiled for 5 minutes before Western blotting. For in vitro binding assay, FLAG-tagged STAT3 proteins expressed in 293T cells were adsorbed onto anti-FLAG G1 affinity resin. Then the mixture was incubated overnight with purified His-Becn1 protein. After washing in 1× TBS buffer, the proteins were eluted by mixing with loading buffer, boiled for 5 minutes, and subjected to analysis.

### Protein docking.

Protein sequences were obtained from the UniProt database (O88597 for mouse Becn1, P42227 for mouse STAT3; Q14457 for human Becn1, P40763 for human STAT3). AlphaFold2 (48) was used to perform de novo modeling and generate a complete 3-dimensional structural model of the Becn1 and STAT3 proteins. We subsequently used HDOCK (46, 47) for protein docking between Becn1 and STAT3. The docking score was calculated based on the ITScore-PP or ITScore-PR iterative scoring function (92). We defined a confidence score dependent on the docking score to indicate the reliability of the binding: confidence_score = 1.0/[1.0+e^0.02x(Docking_Score+150)^]. The most likely interaction form of Becn1 and STAT3 was selected on the basis of the score. Structural analysis and figure panels were prepared via PyMOL (https://pymol.org).

### Statistics.

The statistical significance of differences between 2 groups was examined using unpaired 2-tailed Student’s *t* test or paired 2-tailed Student’s *t* test. A 1-way ANOVA was conducted to assess the differences among multiple groups. Multiple-comparison analysis was used to adjust *P* values. Survival curves were compared using the log-rank test. Statistical significance is indicated by *P < 0.05, **P < 0.01, ***P < 0.001, ****P < 0.0001. Graphs containing error bars show the mean ± SEM. All analyses were performed with GraphPad software (GraphPad Prism 8.3.0).

### Study approval.

The animal experiments were reviewed and approved by the Institutional Committee on Animal Welfare Protection and Ethics of Soochow University.

### Data availability.

Bulk RNA sequencing data were deposited in the NCBI’s Sequence Read Archive database under BioProject PRJNA1039573. Proteome profiles are available via ProteomeXchange with identifier PXD057003. ChIP-Seq data were deposited in the NCBI’s Gene Expression Omnibus (GEO) database under accession number GSE280034. Non-omics data supporting the findings of this study are available within the article and supplemental material. The values for all the data points in the graphs can be found in the supplemental Supporting Data Values file, and all the unedited gels/blots are included in the supplemental material.

## Author contributions

Jianrong Wang conceived the study. WW, XG, NY, and YF designed and performed the experiments. WW, XG, NY, YF, and Jianrong Wang analyzed the experimental data, and JQ and WW analyzed the omics data. LL, CZ, Li Xu, Y Zhu, ZL, NL, XW, ZJ, BL, Lan XU, JD, SZ, Jianrong Wang, Y Zhang, and YY performed parts of the experiments. ZY, YY, and JL provided reagents. WW and XG drafted the manuscript. YF, NY, and Jianrong Wang revised the manuscript. WW and XG contributed equally to this work. Nevertheless, WW worked on this project slightly longer than XG did.

## Supplementary Material

Supplemental data

Unedited blot and gel images

Supporting data values

## Figures and Tables

**Table 1:**
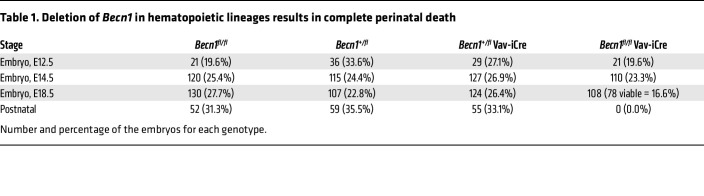
Deletion of *Becn1* in hematopoietic lineages results in complete perinatal death

## Version 1 11/26/2024

In-Press Preview

## Version 2 02/03/2025

Electronic publication

## References

[1] Orkin SH, Zon LI (2008). Hematopoiesis: an evolving paradigm for stem cell biology. Cell.

[2] Medvinsky A (2011). Embryonic origin of the adult hematopoietic system: advances and questions. Development.

[3] Chen MJ (2011). Erythroid/myeloid progenitors and hematopoietic stem cells originate from distinct populations of endothelial cells. Cell Stem Cell.

[4] Baron MH (2012). The embryonic origins of erythropoiesis in mammals. Blood.

[5] Zanjani ED (1993). Liver-derived fetal hematopoietic stem cells selectively and preferentially home to the fetal bone marrow. Blood.

[6] O’Byrne S (2019). Discovery of a CD10-negative B-progenitor in human fetal life identifies unique ontogeny-related developmental programs. Blood.

[7] Popescu DM (2019). Decoding human fetal liver haematopoiesis. Nature.

[8] Azzoni E, Fantin A (2022). Fetal liver hematopoiesis revisited: a precast hierarchy. Nat Cardiovasc Res.

[9] Haas AL (1987). Interferon induces a 15-kilodalton protein exhibiting marked homology to ubiquitin. J Biol Chem.

[10] Yuan W, Krug RM (2001). Influenza B virus NS1 protein inhibits conjugation of the interferon (IFN)-induced ubiquitin-like ISG15 protein. EMBO J.

[11] Radoshevich L (2015). ISG15 counteracts Listeria monocytogenes infection. Elife.

[12] Liu M (2004). Camptothecin induces the ubiquitin-like protein, ISG15, and enhances ISG15 conjugation in response to interferon. J Interferon Cytokine Res.

[13] Perng YC, Lenschow DJ (2018). ISG15 in antiviral immunity and beyond. Nat Rev Microbiol.

[14] Sarkar L (2023). ISG15: its roles in SARS-CoV-2 and other viral infections. Trends Microbiol.

[15] Munnur D (2021). Altered ISGylation drives aberrant macrophage-dependent immune responses during SARS-CoV-2 infection. Nat Immunol.

[16] Cao X (2021). ISG15 secretion exacerbates inflammation in SARS-CoV-2 infection. Nat Immunol.

[17] Liang XH (1998). Protection against fatal Sindbis virus encephalitis by beclin, a novel Bcl-2-interacting protein. J Virol.

[18] Fimia GM (2007). Ambra1 regulates autophagy and development of the nervous system. Nature.

[19] Liang C (2006). Autophagic and tumour suppressor activity of a novel Beclin1-binding protein UVRAG. Nat Cell Biol.

[20] Furuya N (2005). The evolutionarily conserved domain of Beclin 1 is required for Vps34 binding, autophagy and tumor suppressor function. Autophagy.

[21] Sun Q (2008). Identification of Barkor as a mammalian autophagy-specific factor for Beclin 1 and class III phosphatidylinositol 3-kinase. Proc Natl Acad Sci U S A.

[22] Liang XH (1999). Induction of autophagy and inhibition of tumorigenesis by beclin 1. Nature.

[23] Qu X (2003). Promotion of tumorigenesis by heterozygous disruption of the beclin 1 autophagy gene. J Clin Invest.

[24] Yue Z (2003). Beclin 1, an autophagy gene essential for early embryonic development, is a haploinsufficient tumor suppressor. Proc Natl Acad Sci U S A.

[25] Fernandez AF (2018). Disruption of the beclin 1-BCL2 autophagy regulatory complex promotes longevity in mice. Nature.

[26] Liu Y (2020). TLR9 and beclin 1 crosstalk regulates muscle AMPK activation in exercise. Nature.

[27] Levine B (2015). Beclin orthologs: integrative hubs of cell signaling, membrane trafficking, and physiology. Trends Cell Biol.

[28] Wu S (2018). Targeting the potent Beclin 1-UVRAG coiled-coil interaction with designed peptides enhances autophagy and endolysosomal trafficking. Proc Natl Acad Sci U S A.

[29] Tran S (2024). BECLIN1 is essential for intestinal homeostasis involving autophagy-independent mechanisms through its function in endocytic trafficking. Commun Biol.

[30] Kuramoto K (2021). The autophagy protein Becn1 improves insulin sensitivity by promoting adiponectin secretion via exocyst binding. Cell Rep.

[31] Rohatgi RA (2015). Beclin 1 regulates growth factor receptor signaling in breast cancer. Oncogene.

[32] Seo J (2020). Beclin 1 functions as a negative modulator of MLKL oligomerisation by integrating into the necrosome complex. Cell Death Differ.

[33] Tan P (2019). Myeloid loss of Beclin 1 promotes PD-L1hi precursor B cell lymphoma development. J Clin Invest.

[34] Wang YT (2020). Select autophagy genes maintain quiescence of tissue-resident macrophages and increase susceptibility to Listeria monocytogenes. Nat Microbiol.

[35] Thomas MR, Storey RF (2015). The role of platelets in inflammation. Thromb Haemost.

[36] Chen Z (2022). Intestinal IL-33 promotes platelet activity for neutrophil recruitment during acute inflammation. Blood.

[37] Crainiciuc G (2022). Behavioural immune landscapes of inflammation. Nature.

[38] Thery F (2021). Proteomics mapping of the ISGylation landscape in innate immunity. Front Immunol.

[39] Levy DE (2002). STATs: transcriptional control and biological impact. Nat Rev Mol Cell Biol.

[40] Wen Z (1995). Maximal activation of transcription by Stat1 and Stat3 requires both tyrosine and serine phosphorylation. Cell.

[41] Zhang X (1995). Requirement of serine phosphorylation for formation of STAT-promoter complexes. Science.

[42] Bromberg J, Wang TC (2009). Inflammation and cancer: IL-6 and STAT3 complete the link. Cancer Cell.

[43] Gajjela BK, Zhou MM (2022). Calming the cytokine storm of COVID-19 through inhibition of JAK2/STAT3 signaling. Drug Discov Today.

[44] Eble JA (2018). Titration ELISA as a method to determine the dissociation constant of receptor ligand interaction. J Vis Exp.

[45] Jarmoskaite I (2020). How to measure and evaluate binding affinities. Elife.

[46] Feng Y (2022). Docking and scoring for nucleic acid-ligand interactions: principles and current status. Drug Discov Today.

[47] Feng Y (2021). NLDock: a fast nucleic acid-ligand docking algorithm for modeling RNA/DNA-ligand complexes. J Chem Inf Model.

[48] Jumper J (2021). Highly accurate protein structure prediction with AlphaFold. Nature.

[49] Kuma A (2004). The role of autophagy during the early neonatal starvation period. Nature.

[50] Komatsu M (2005). Impairment of starvation-induced and constitutive autophagy in Atg7-deficient mice. J Cell Biol.

[51] Kalish BT (2021). Maternal immune activation in mice disrupts proteostasis in the fetal brain. Nat Neurosci.

[52] Han VX (2021). Maternal immune activation and neuroinflammation in human neurodevelopmental disorders. Nat Rev Neurol.

[53] Hofsink N (2024). The fetal programming effect of maternal immune activation (MIA) on the offspring’s immune system. Semin Immunopathol.

[54] Krug RM (2005). Properties of the ISG15 E1 enzyme UbE1L. Methods Enzymol.

[55] Zhao C (2004). The UbcH8 ubiquitin E2 enzyme is also the E2 enzyme for ISG15, an IFN-α/β-induced ubiquitin-like protein. Proc Natl Acad Sci U S A.

[56] Wong JJ (2006). HERC5 is an IFN-induced HECT-type E3 protein ligase that mediates type I IFN-induced ISGylation of protein targets. Proc Natl Acad Sci U S A.

[57] Zhao C (2005). Human ISG15 conjugation targets both IFN-induced and constitutively expressed proteins functioning in diverse cellular pathways. Proc Natl Acad Sci U S A.

[58] Knight E (1991). IFN-induced 15-kDa protein is released from human lymphocytes and monocytes. J Immunol.

[59] Dos Santos PF, Mansur DS (2017). Beyond ISGlylation: functions of free intracellular and extracellular ISG15. J Interferon Cytokine Res.

[60] Liu G (2021). ISG15-dependent activation of the sensor MDA5 is antagonized by the SARS-CoV-2 papain-like protease to evade host innate immunity. Nat Microbiol.

[61] Shin D (2020). Papain-like protease regulates SARS-CoV-2 viral spread and innate immunity. Nature.

[62] Blanco-Melo D (2020). Imbalanced host response to SARS-CoV-2 drives development of COVID-19. Cell.

[63] Joyce MM (2005). Interferon stimulated gene 15 conjugates to endometrial cytosolic proteins and is expressed at the uterine-placental interface throughout pregnancy in sheep. Endocrinology.

[64] Schanz A (2014). Interferon stimulated gene 15 expression at the human embryo-maternal interface. Arch Gynecol Obstet.

[65] Desai SD (2006). Elevated expression of ISG15 in tumor cells interferes with the ubiquitin/26S proteasome pathway. Cancer Res.

[66] Baldridge MT (2010). Quiescent haematopoietic stem cells are activated by IFN-gamma in response to chronic infection. Nature.

[67] Sato T (2009). Interferon regulatory factor-2 protects quiescent hematopoietic stem cells from type I interferon-dependent exhaustion. Nat Med.

[68] Takizawa H (2017). Pathogen-induced TLR4-TRIF innate immune signaling in hematopoietic stem cells promotes proliferation but reduces competitive fitness. Cell Stem Cell.

[69] Zhao JL (2014). Conversion of danger signals into cytokine signals by hematopoietic stem and progenitor cells for regulation of stress-induced hematopoiesis. Cell Stem Cell.

[70] Sawamiphak S (2014). Interferon gamma signaling positively regulates hematopoietic stem cell emergence. Dev Cell.

[71] Li Y (2014). Inflammatory signaling regulates embryonic hematopoietic stem and progenitor cell production. Genes Dev.

[72] He Q (2015). Inflammatory signaling regulates hematopoietic stem and progenitor cell emergence in vertebrates. Blood.

[73] Espin-Palazon R (2014). Proinflammatory signaling regulates hematopoietic stem cell emergence. Cell.

[74] Lefkopoulos S (2020). Repetitive elements trigger RIG-I-like receptor signaling that regulates the emergence of hematopoietic stem and progenitor cells. Immunity.

[75] Gao Y (2020). m^6^A modification prevents formation of endogenous double-stranded RNAs and deleterious innate immune responses during hematopoietic development. Immunity.

[76] Cadwell K (2008). A key role for autophagy and the autophagy gene Atg16l1 in mouse and human intestinal Paneth cells. Nature.

[77] Saitoh T (2008). Loss of the autophagy protein Atg16L1 enhances endotoxin-induced IL-1beta production. Nature.

[78] Kimmey JM (2015). Unique role for ATG5 in neutrophil-mediated immunopathology during M. tuberculosis infection. Nature.

[79] Cunha LD (2018). LC3-associated phagocytosis in myeloid cells promotes tumor immune tolerance. Cell.

[80] Houtman J (2019). Beclin1-driven autophagy modulates the inflammatory response of microglia via NLRP3. EMBO J.

[81] Wang YT (2023). Myeloid autophagy genes protect mice against fatal TNF- and LPS-induced cytokine storm syndromes. Autophagy.

[82] Yang X (2020). BECN1 modulates hematopoietic stem cells by targeting Caspase-3-GSDME-mediated pyroptosis. Blood Sci.

[83] Gantier MP, Williams BR (2007). The response of mammalian cells to double-stranded RNA. Cytokine Growth Factor Rev.

[84] Chen MJ (2009). Runx1 is required for the endothelial to haematopoietic cell transition but not thereafter. Nature.

[85] Wu T (2021). clusterProfiler 4.0: a universal enrichment tool for interpreting omics data. Innovation (Camb).

[86] Bedre R (2016). Transcriptome analysis of smooth cordgrass (Spartina alterniflora Loisel), a monocot halophyte, reveals candidate genes involved in its adaptation to salinity. BMC Genomics.

[87] Martin M (2011). Cutadapt removes adapter sequences from high-throughput sequencing reads. EMBnet J.

[88] Bolger AM (2014). Trimmomatic: a flexible trimmer for Illumina sequence data. Bioinformatics.

[89] Langmead B, Salzberg SL (2012). Fast gapped-read alignment with Bowtie 2. Nat Methods.

[90] Zhang Y (2008). Model-based Analysis of ChIP-Seq (MACS). Genome Biol.

[91] Yu G (2015). ChIPseeker: an R/Bioconductor package for ChIP peak annotation, comparison and visualization. Bioinformatics.

[92] Huang SY, Zou X (2008). An iterative knowledge-based scoring function for protein-protein recognition. Proteins.

