# Supplementary material for: Beclin 1 prevents ISG15-mediated cytokine storms to secure fetal hematopoiesis and survival
Source: J Clin Invest. 2025 Feb 3;135(3):e177375. doi: 10.1172/JCI177375 (PMC11785930; doi:10.1172/JCI177375)
Supplement: Supplemental data [file jci-135-177375-s216.pdf]

Supplemental Figures and Tables

Beclin 1 prevents ISG15-mediated cytokine storms to secure fetal hematopoiesis and survival

Wen Wei,[1,2,3,4] Xueqin Gao,[1,2,3] Jiawei Qian,[1] Lei Li,[1,4] Chen Zhao,[1,4] Li Xu,[1] Yanfei Zhu,[1] Zhenzhen Liu,[1] Nengrong Liu,[1] Xueqing Wang,[1] Zhicong Jin,[1] Bowen Liu,[1] Lan Xu,[1] Jin Dong,[1] Suping Zhang,[1,2,3] Jiarong Wang,[1] Yumu Zhang,[1] Yao Yu,[1] Zhanjun Yan,[4] Yanjun Yang,[4] Jie Lu,[4] Yixuan Fang,[1,2,3,4] Na Yuan,[1,2,3,4] Jianrong Wang[1,2,3,4]

[1] Research Center for Blood Engineering and Manufacturing, Cyrus Tang Medical Institute, Soochow University, Suzhou 215123, China.

[2] National Clinical Research Center for Hematologic Diseases, Key Laboratory of Thrombosis and Hemostasis Ministry of Health, Collaborative Innovation Center of Hematology, Jiangsu Institute of Hematology, Institute of Blood and Marrow Transplantation, The First Affiliated Hospital of Soochow University, Suzhou 213006, China.

[3] State Key Laboratory of Radiation Medicine and Protection, Soochow University, Suzhou 215123, China.

[4] The Ninth Affiliated Suzhou Hospital of Soochow University, Suzhou, China

Content

Supplemental Figures 1-14
Supplemental Tables 1-3

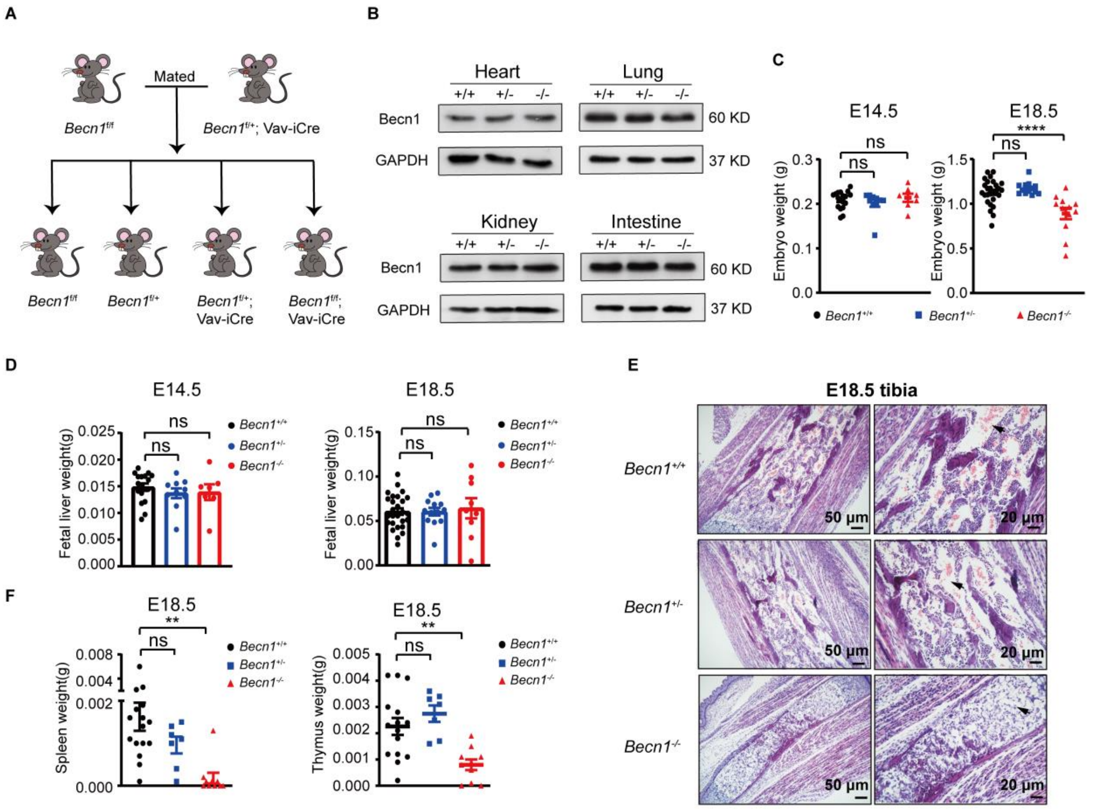

Supplemental Figure 1. Characterization of *Becn1*$^{f/f}$;Vav-iCre mice. (**A**) Breeding scheme for obtaining *Becn1*$^{+/+}$, *Becn1*$^{+/-}$ and *Becn1*$^{-/-}$ embryos. (**B**) Western blotting for Becn1 and GAPDH in E18.5 non-hematopoietic tissues (heart, lung, kidney, intestine). (**C**) Weight analysis of E14.5 and E18.5 embryos. *Becn1*$^{-/-}$ mice is lighter than *Becn1*$^{+/+}$ and *Becn1*$^{+/-}$ mice at E18.5 (n=7-27). (**D**) Fetal liver weight analysis of E14.5 (left) and E18.5 (right) (n=7-27). (**E**) Representative H&E staining of tibia sections from E18.5 embryos. Black arrows indicate erythroid cells. (**F**) Spleen and thymus coefficient in E18.5 (n=7-16). ***P*<0.01; *****P*<0.0001. One-way ANOVA (Dunnett's multiple comparisons test). Data represent mean±SEM.

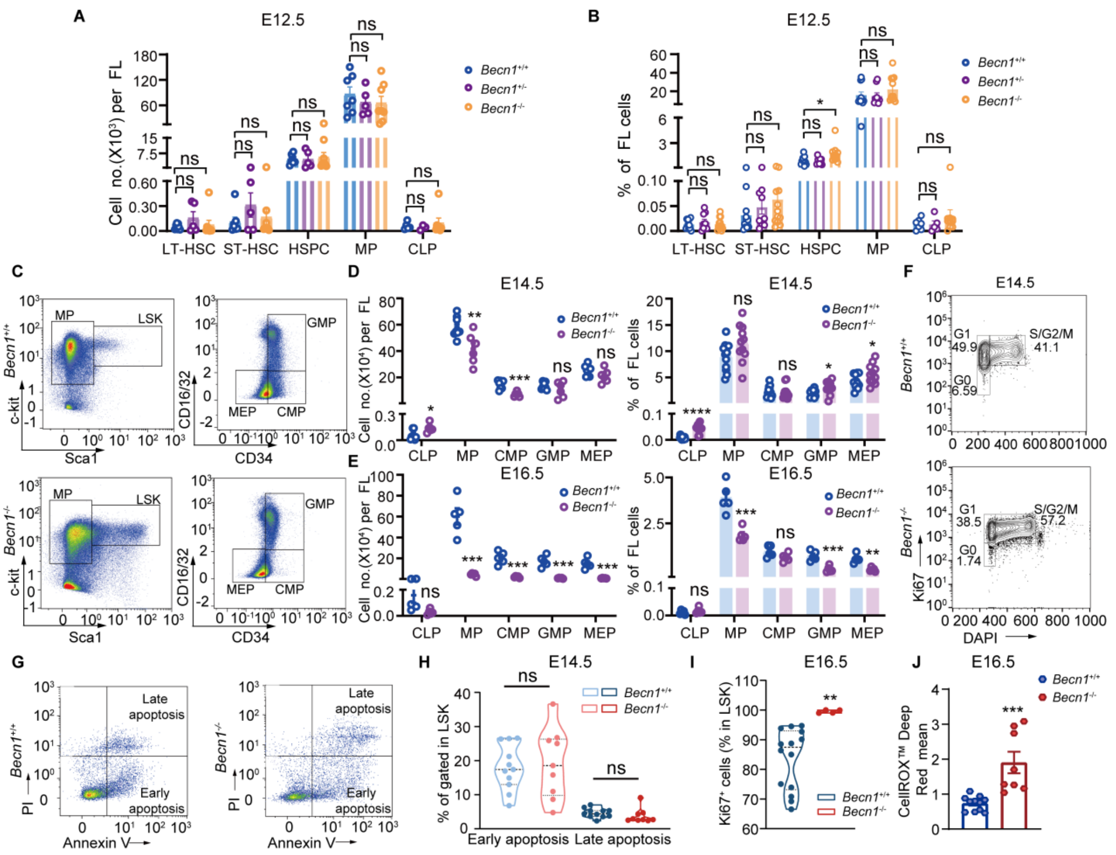

Supplemental Figure 2. Deletion of *Becn1* led to abnormal hematopoietic cells. (**A-B**) Absolute number and percentage of HSCs and HSPCs ($Lin^-Sca\text{-}1^+c\text{-}Kit^+$, LSK) were detected by flow cytometry in fetal livers of $Becn1^{+/+}$, $Becn1^{+/-}$ and $Becn1^{-/-}$ E12.5 embryos (n=3-12). (**C**) Representative flow cytometer plots of myeloid cells analysis. GMP ($MPCD16/32^+CD34^+$), CMP ($MPCD16/32^-CD34^+$) and MEP ($MPCD16/32^-CD34^-$). (**D-E**) Absolute number (left) and percentage (right) of myeloid and lymphoid progenitor cells were detected by flow cytometry in fetal livers of $Becn1^{+/+}$ and $Becn1^{-/-}$ embryos (n=5-12). D, E14.5 fetal liver cells; E, E16.5 fetal liver cells. (**F**) Representative FCM profiles of cell cycle analysis. (**G**) Representative FCM profiles of apoptosis analysis. (**H**) Apoptosis analysis of LSK cells by flow cytometry in $Becn1^{+/+}$ and $Becn1^{-/-}$ E14.5 fetal liver cells (n=9-11). (**I**) Percentage of $Ki67^+$ in LSK cells were detected by flow cytometry in $Becn1^{+/+}$ or $Becn1^{-/-}$ E16.5 fetal liver cells (n=4-14). (**J**) ROS analysis of LSK cells in E16.5 fetal liver cells (n=8-11). **P*<0.05; ***P*<0.01; ****P*<0.001. Unpaired two-tailed Student's *t*-test. One-way ANOVA (Dunnett's multiple comparisons test) (A-B). Data represent mean±SEM.

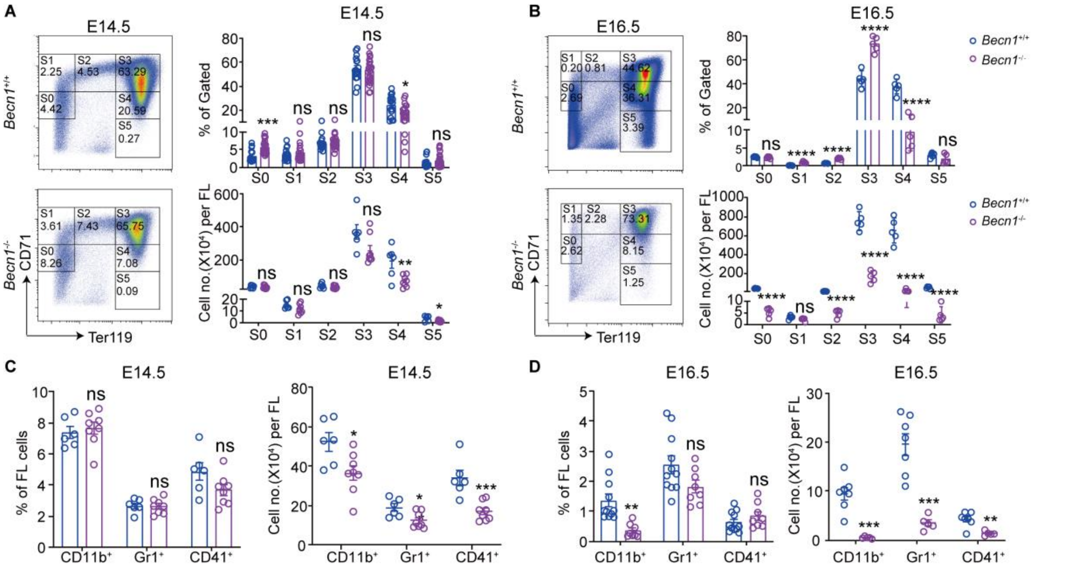

Supplemental Figure 3. Deletion of *Becn1* led to abnormal erythropoiesis and myelopoiesis. (**A-B**) Representative flow cytometric analysis plot of erythropoiesis in the fetal livers of E14.5 (A) or E16.5 (B) *Becn1*$^{+/+}$ and *Becn1*$^{-/-}$ mice. Left panel, representative flow cytometric analysis plot. S0-S5: Gates of different erythroblast populations according to their expression levels of CD71 and TER119. S0: Primitive progenitor cells (BFU-E and CFU-E); S1: CFU-E; S2: Proerythroblasts; S3: Early and late basophilic erythroblasts; S4: Chromatophilic and orthochromatophilic erythroblasts; S5: Late orthochromatophilic erythroblasts and reticulocytes. Right panel, flow cytometric statistical analysis of the percentage and number of erythroid cells at different stages of development. The percentage and absolute number of erythroid cells were analyzed by flow cytometry and are shown in the left panel. E14.5: n=6-22; E16.5: n=5. (**C-D**) The percentage and absolute number of myeloid cells in E14.5 (C) or E16.5 (D) fetal livers were analyzed via flow cytometry. Left panel, flow cytometric statistical analysis of the percentage of myeloid cells in fetal livers. Right panel, flow cytometric statistical analysis of the absolute number of myeloid cells in the fetal liver. E14.5: n=6-8; E16.5: n=5-11. **P*<0.05; ***P*<0.01; ****P*<0.001; *****P*<0.0001. Unpaired two-tailed Student's *t*-test was used. The data represent the mean±SEM.

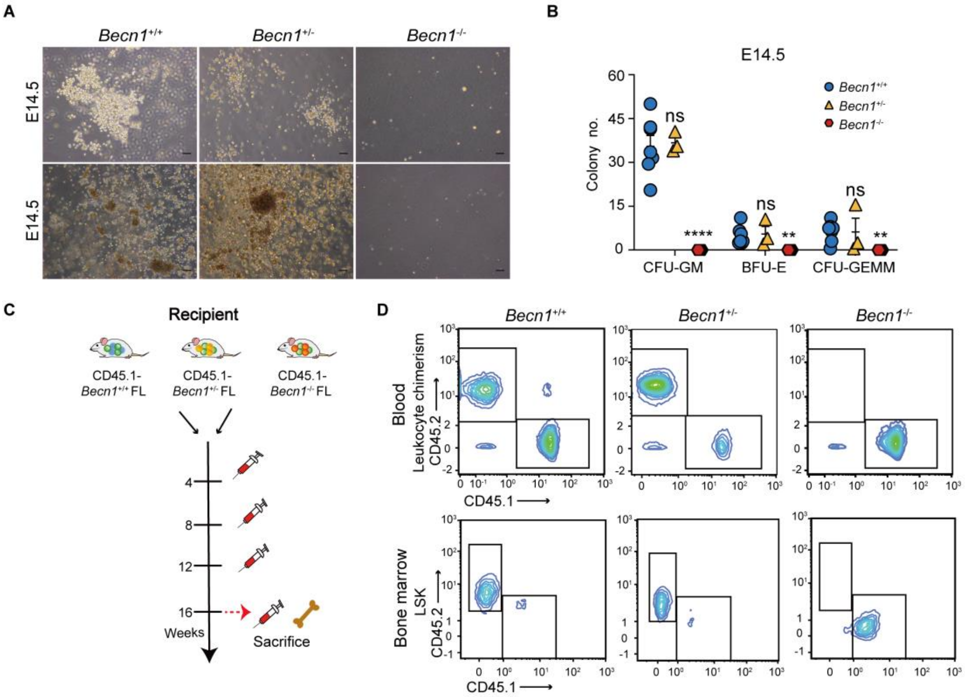

Supplemental Figure 4. Deletion of Becn1 impairs colony formation and reconstitution capacity of the fetal liver cells. (**A**) Representative pictures of colonies formed from *Becn1*$^{+/+}$, *Becn1*$^{+/-}$ and *Becn1*$^{-/-}$ fetal liver cells in E14.5. Scar bar: 100 µm. (**B**) Myeloid progenitor cell colony formation of fetal liver cells (2 X 10^4) from *Becn1*$^{+/+}$, *Becn1*$^{+/-}$ and *Becn1*$^{-/-}$ embryos at E14.5. Colonies were examined at day 7 of culturing. Average numbers of colony-forming unit-granulocyte/monocyte (CFU-GM), burst-forming unit-erythroid (BFU-E) and colony-forming unit-granulocyte/erythrocyte /monocyte/megakaryocyte (CFU-GEMM) colonies are presented (n=3-8). (**C**) Schematic protocol for detecting recipient mice after competitive transplantation. (**D**) Scheme of flow cytometric analysis of peripheral blood and bone marrow in recipient mice. ***P*<0.001; *****P*<0.0001. One-way ANOVA (Dunnett's multiple comparisons test). Data represent mean±SEM.

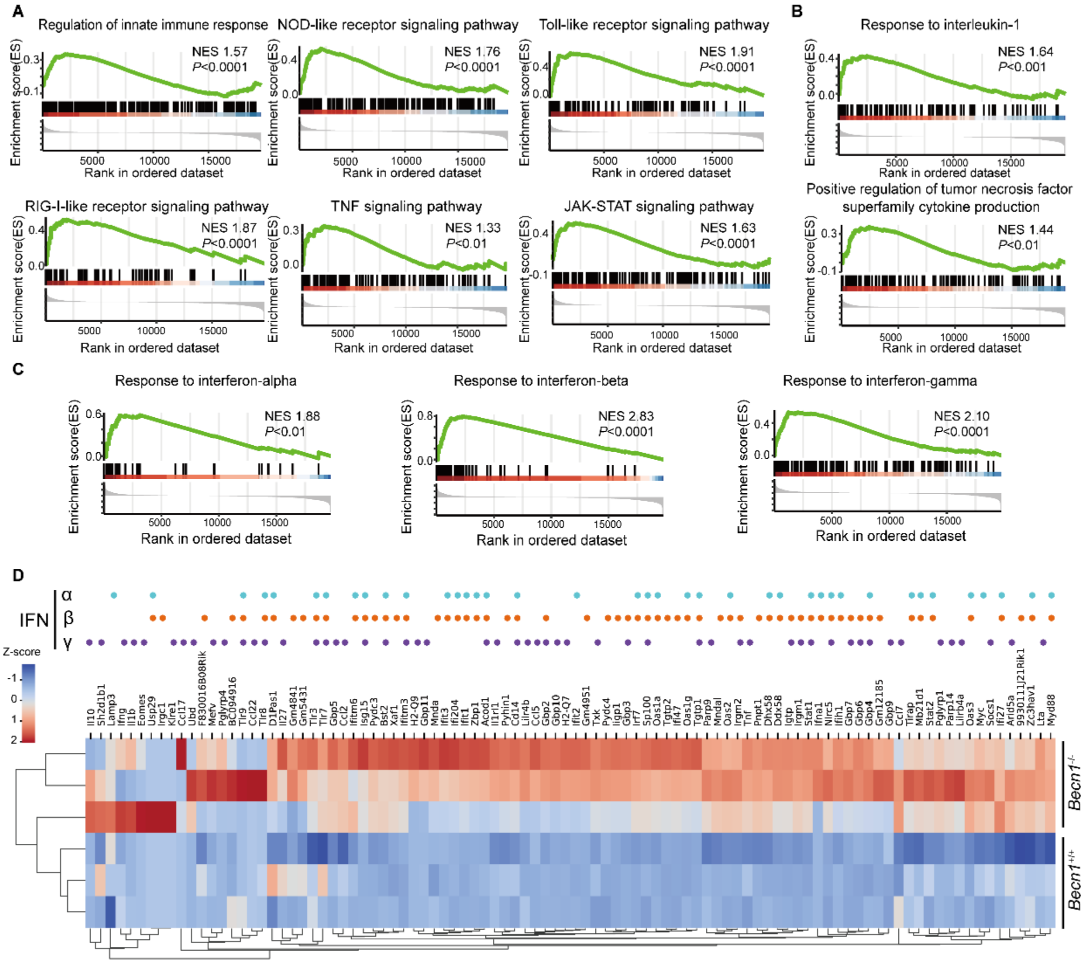

Supplemental Figure 5. Transcriptomics profiling reveals decontrolled innate immune responses and inflammation in the *Becn1*-deleted fetus. (**A**) Gene set enrichment analysis of *Becn1*$^{+/+}$ and *Becn1*$^{-/-}$ LSK cells in innate immune pathway. Normalized enrichment score |NES|>1, *P*<0.05. (**B-C**) Gene set enrichment analysis of *Becn1*$^{+/+}$ and *Becn1*$^{-/-}$ LSK cells in inflammatory signaling pathway. Normalized enrichment score |NES|>1, *P*<0.05. (**D**) Heatmap of genes in IFN signaling pathway that differentially expressed upon *Becn1* deletion using RNA-Seq results.

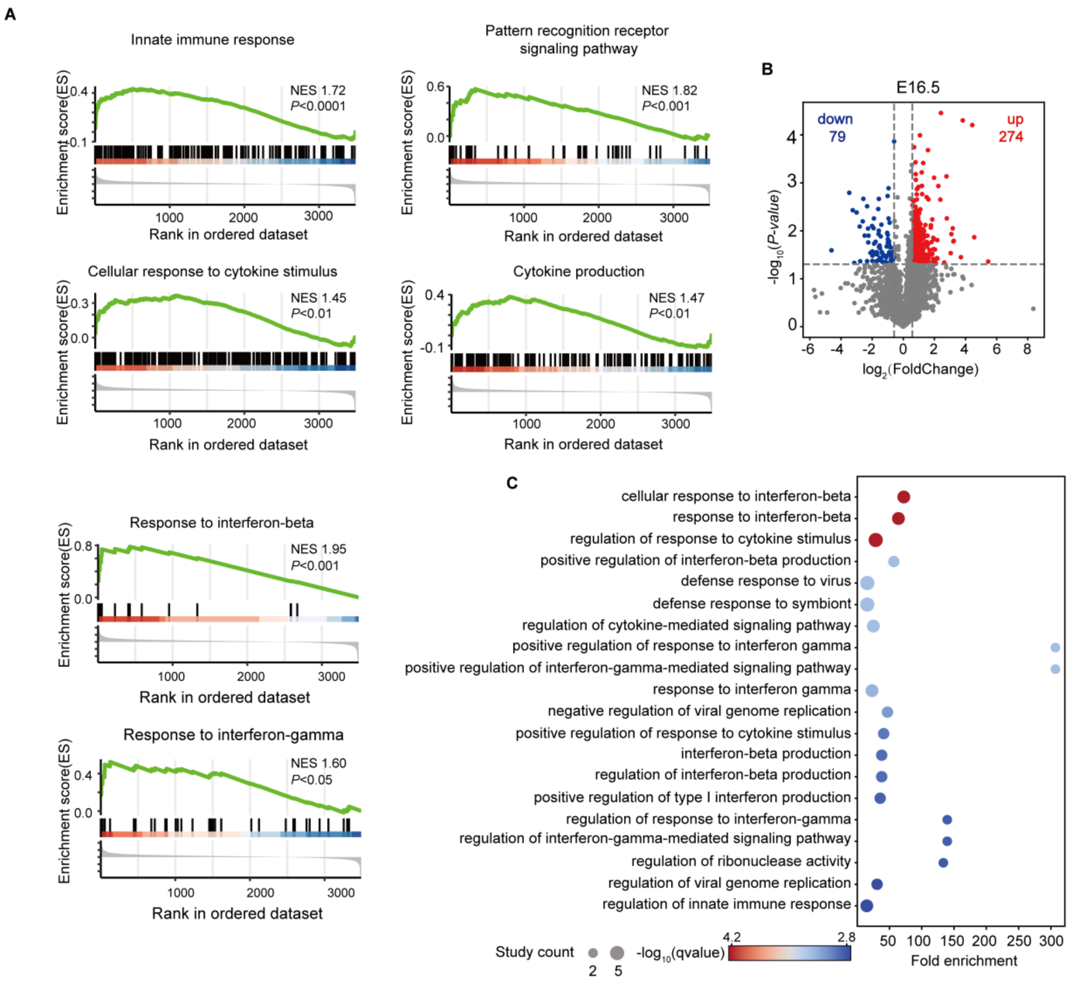

Supplemental Figure 6. Deletion of *Becn1* displays hyperactivated innate immune responses in the *Becn1*-deleted HSPCs by proteomics analysis. (**A**) Gene set enrichment analysis of *Becn1*$^{+/+}$ and *Becn1*$^{-/-}$ LSK cells in innate immune and inflammation pathway from E14.5 proteomics. Normalized enrichment score |NES|>1, *P*<0.05. (**B**) Volcano plot showed differentially expressed proteins in *Becn1*$^{+/+}$ and *Becn1*$^{-/-}$ LSK cells from E16.5 FLs (n=3). (**C**) GO enrichment analysis of correlated differentially expressed proteins/genes from E14.5 transcriptomics and proteomics.

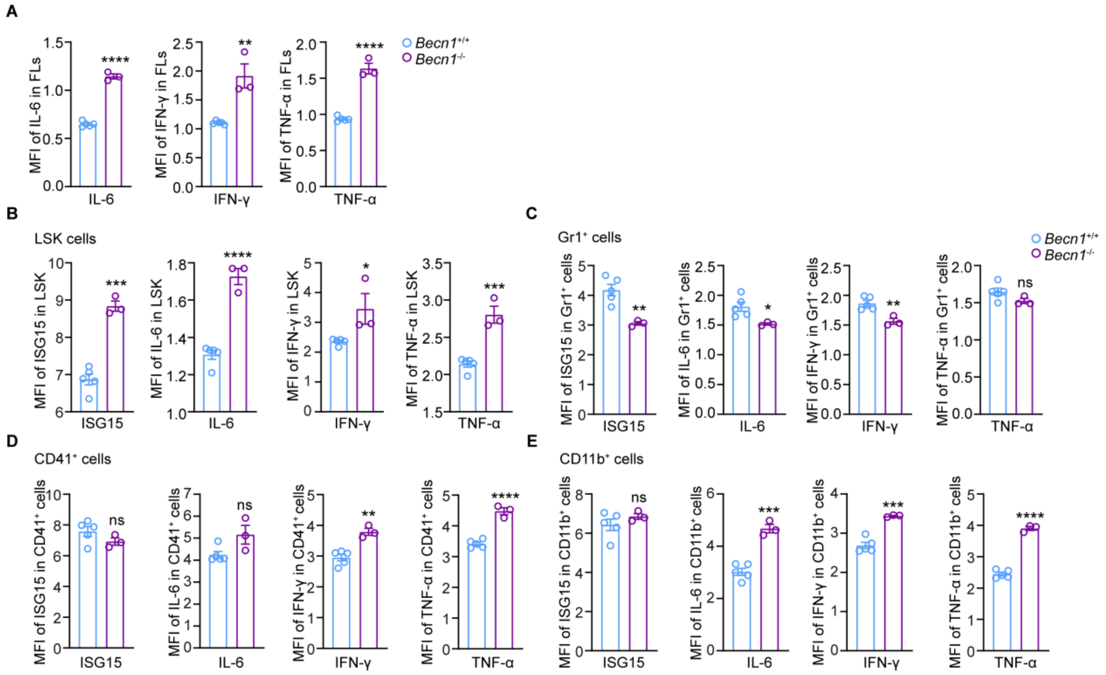

Supplemental Figure 7. Multicolor flow cytometric measurement of cytokine levels in total and four populations of fetal liver cells at E14.5 after secretion blockade. (**A**) Cytokines were increased in total fetal liver cells. (**B**) Cytokines were increased in LSK HSPCs of fetal livers. (**C-E**) Cytokine levels were altered in three populations of mature blood cells with less significance. To obtain objective results, we treated single cell suspension of the fetal liver cells with BD GolgiStop^{TM} protein transport inhibitor containing monensin to block the leakage of cytokines out of the expressing cells before measuring the expression levels of cytokines, using the protocol provided by the manufacturer. n=3-5. **P*<0.05; ***P*<0.01; ****P*<0.001; *****P*<0.0001. Unpaired two-tailed Student's *t*-test. Data represent the mean±SEM.

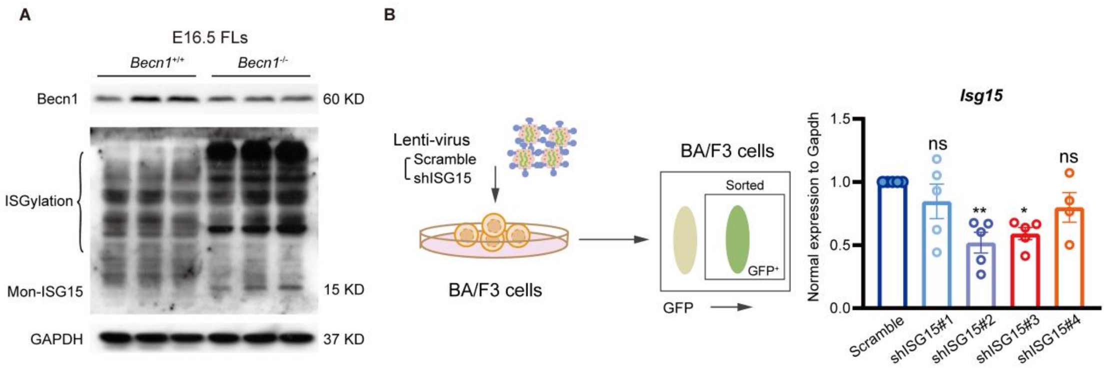

Supplemental Figure 8. Detection of ISG15 expression. (**A**) Detection of ISG15 levels by Western blotting in E16.5 fetal liver cells from *Becn1*$^{f/f}$;Vav-iCre mice. (**B**) Validation of *ISG15* shRNA knockout efficiency in lentivirus infected Baf3 cells. Schematic procedure of lentivirus infected Baf3 cells (left panel). Real-time qPCR analysis of *ISG15* expression in GFP^{+} cells after sorting of lentivirus infected Baf3 cells (right panel) (n=4-5). *P<0.05; **P<0.01. One-way ANOVA (Dunnett's multiple comparisons test). Data represent mean±SEM.

Note: Unlike E12.5 and E14.5 when fetal liver cells are primarily hematopoietic cells and *Becn1* deletion by Vav-Cre is efficient, at E16.5, fetal liver cells consist of both hematopoietic cells and a significant portion of non-hematopoietic cells. Vav-Cre is unable to delete *Becn1* in non-hematopoietic lineage cells, leading to a higher level of Becn1 protein in the total liver cells of E16.5 fetus than that of E12.5 or E14.5 fetus.

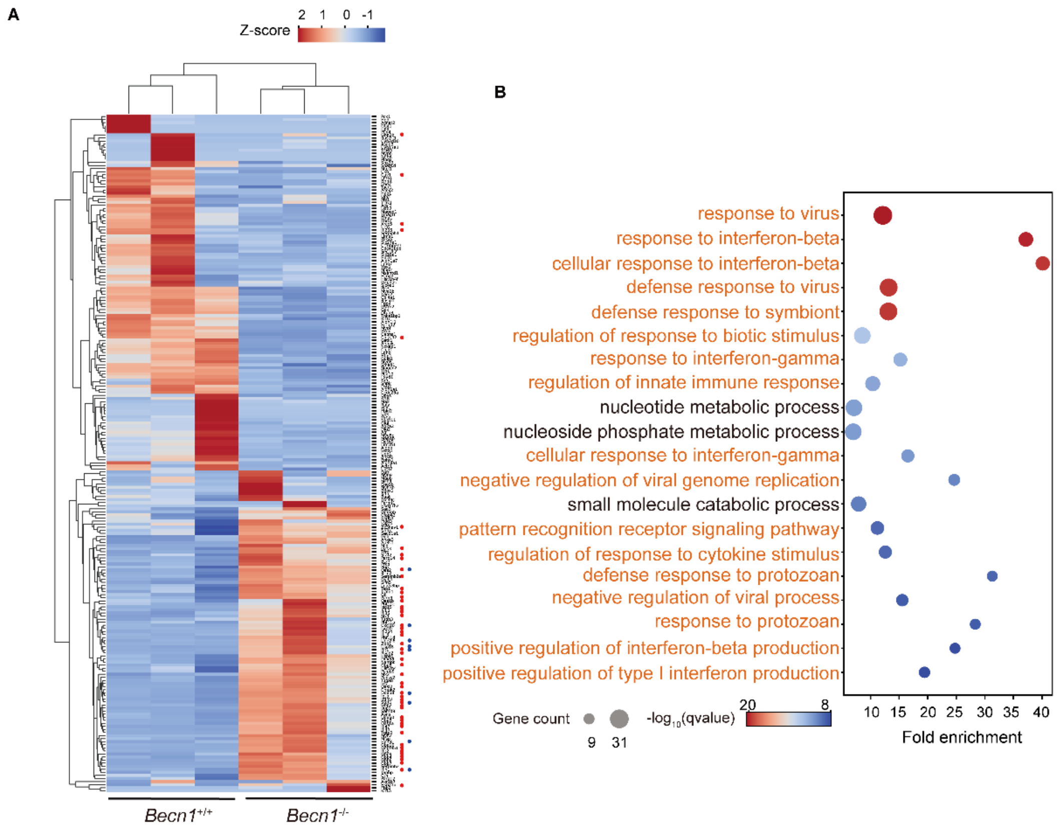

Supplemental Figure 9. The related analysis of ISG15 pull down proteins with transcriptome differentially expressed genes. (**A**) Heatmap of correlated genes from ISG15 target proteins compared with differentially expressed genes in E14.5 LSK transcriptome. (**B**) GO enrichment analysis of correlated genes from (A).

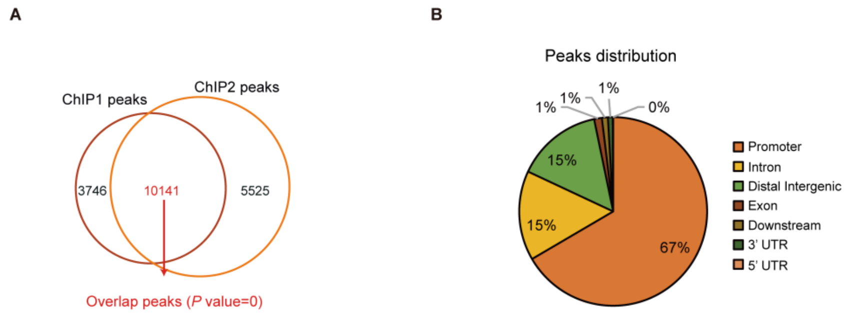

Supplemental Figure 10. The binding peaks from Chip-Seq analysis. (**A**) Venn plot displayed the overlapped peaks from twice repeated Chip-seq (*P*-value=0.001; Fold Change>4). (**B**) The distribution of overlapped peaks on the genetic elements.

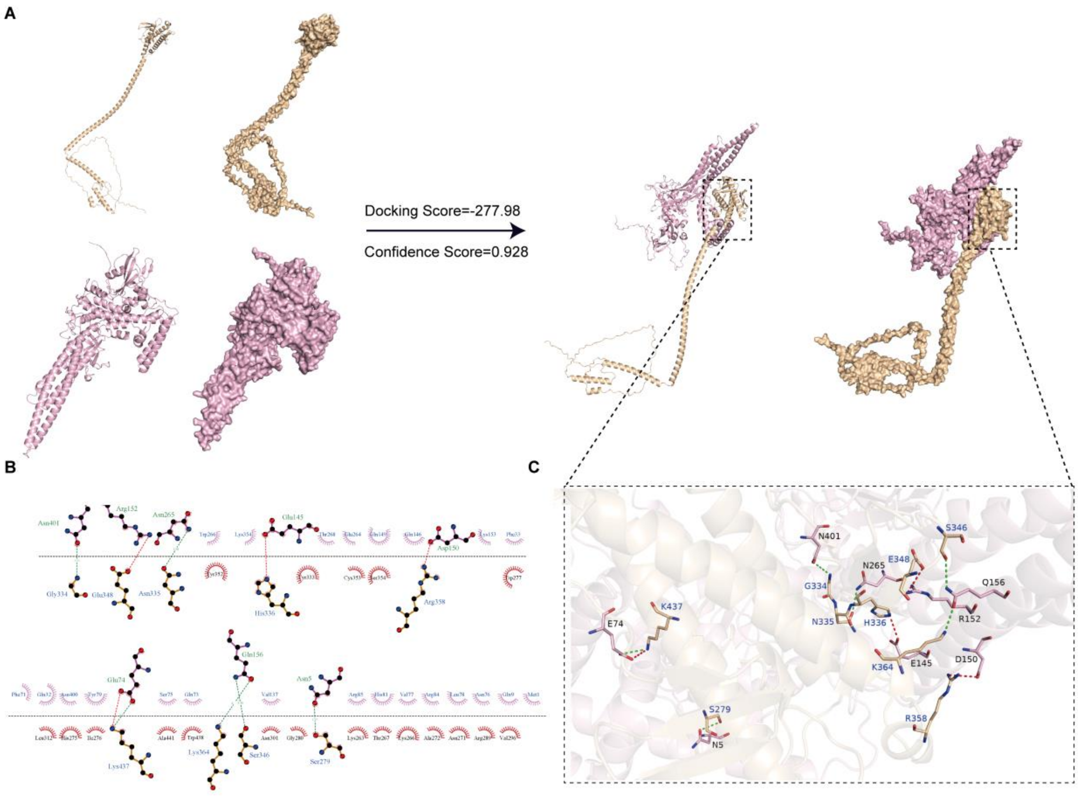

Supplemental Figure 11. The protein-protein docking analysis of human Becn1 and human STAT3. (**A**) The structure and protein-protein docking model of human Becn1 (upper panel) and human STAT3 (lower panel). (**B**) 2D interaction patterns between human Becn1 and human STAT3: The tooth-like amino acids represent hydrophobic interactions, the green dashed lines represent hydrogen bonding, and the red dashed lines represent salt bridge interactions. (**C**) 3D interaction patterns between human Becn1 and human STAT3.

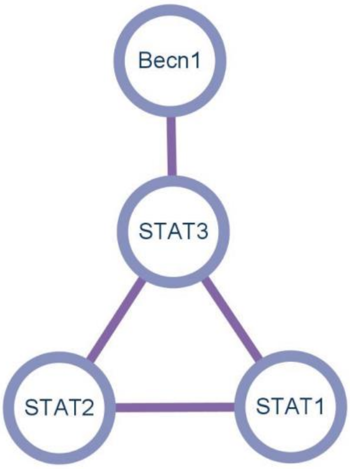

Supplemental Figure 12. Becn1 interact with STAT3 rather than STAT1 or STAT2. The interaction analysis of Becn1, STAT3, STAT1 and STAT2 by STRING database. PPI enrichment *P*-value: 0.0311.

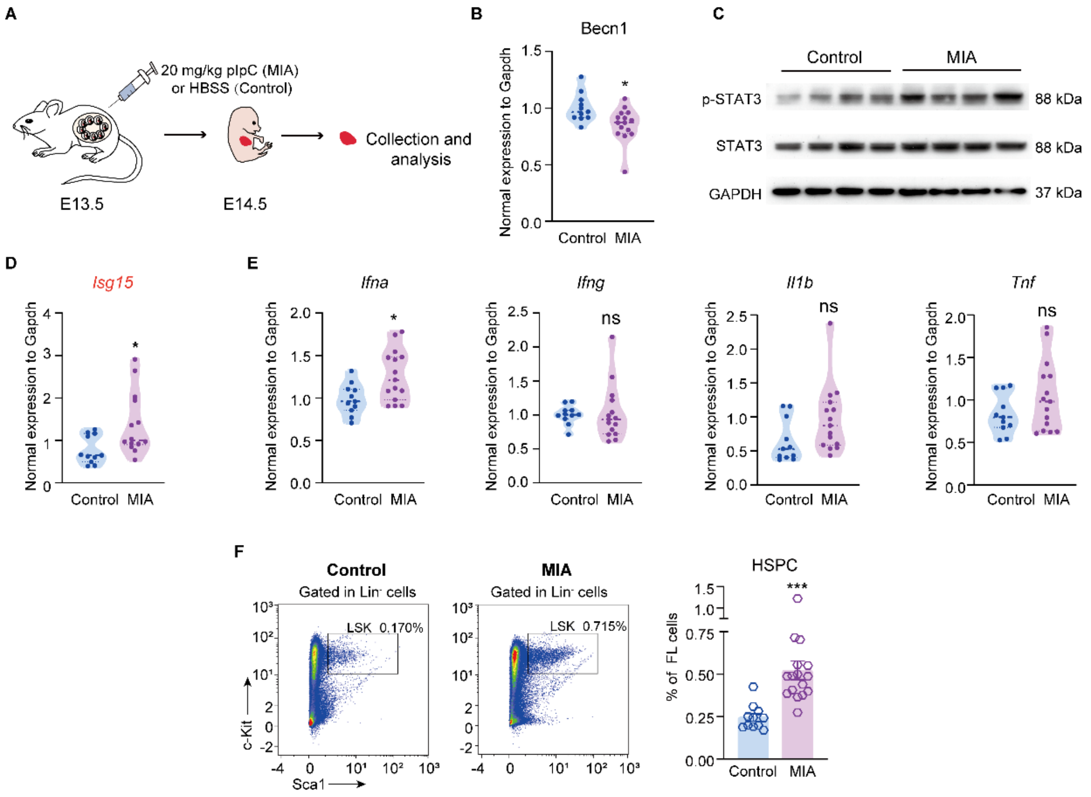

Supplemental Figure 13. The fetal Becn1-ISG15 axis is implicated in protection from maternal immune activation (MIA). (**A**) Schematic protocol for the construction of the MIA model. (**B**) Real-time qPCR analysis of the Becn1 gene (n= 11-15). (**C**) Western blot analysis of p-STAT3, STAT3 and GAPDH in the MIA model. (**D-E**) RT–qPCR analysis of *Isg15* and inflammatory cytokine genes (n= 11-15). (**F**) Percentages of HSPCs in E14.5 fetal liver cells from the MIA model were determined via flow cytometry analysis (n= 11-16). **P*<0.05; ****P*<0.001. Unpaired two-tailed Student's *t*-test was used. The data represent the mean±SEM.

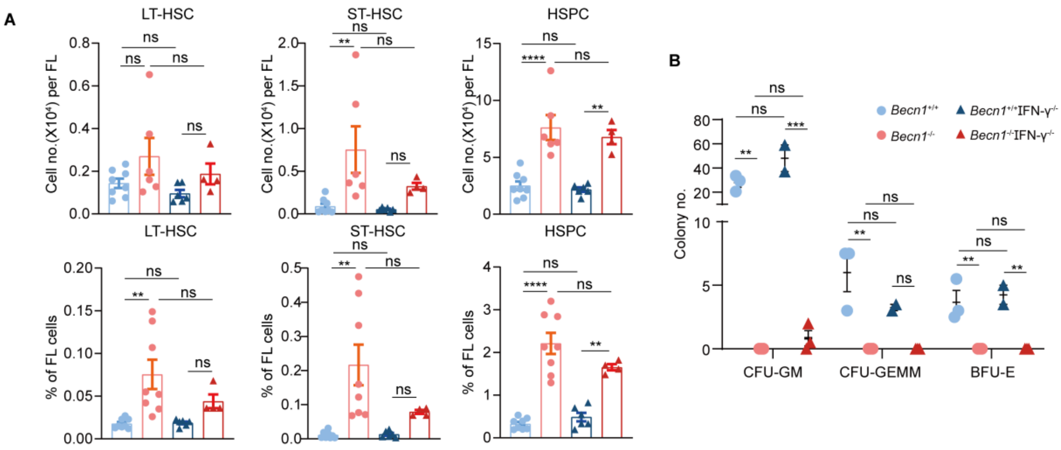

Supplemental Figure 14. Becn1 regulation of fetal hematopoiesis does not depend on IFN-γ signaling. (**A**) Absolute number (upper) and percentage (lower) of HSCs and HSPCs were detected by flow cytometry in the fetal livers of *Becn1*$^{+/+}$, *Becn1*$^{-/-}$, *Becn1*$^{+/+}$IFN-γ$^{-/-}$ and *Becn1*$^{-/-}$IFN-γ$^{-/-}$ E14.5 mice (n=4-8). (**B**) Myeloid progenitor cell colony formation of fetal liver cells (2X10^{4}) from *Becn1*$^{+/+}$, *Becn1*$^{-/-}$, *Becn1*$^{+/+}$IFN-γ$^{-/-}$ and *Becn1*$^{-/-}$IFN-γ$^{-/-}$ embryos at E14.5. Colonies were examined at day 7 of culturing (n=2-3). ***P*<0.01; ****P*<0.001; *****P*<0.0001. One-way ANOVA (Tukey's multiple comparisons test). Data represent mean±SEM.

Supplementary Table 1. Key reagents information

REAGENT	SOURCE	IDENTIFIER
Mouse CD2-FITC antibody	Biolegend	100105
Mouse CD3-FITC antibody	Biolegend	100204
Mouse CD5-FITC antibody	Biolegend	100606
Mouse CD8a-FITC antibody	Biolegend	100706
Mouse Ter119-FITC antibody	Biolegend	116206
Mouse Gr1-FITC antibody	Biolegend	108406
Mouse B220-FITC antibody	Biolegend	103206
Mouse Sca-1-PerCP/Cyanine5.5 antibody	Biolegend	122524
Mouse CD117-APC eFluor 780 antibody	eBioscience	47-1172-82
Mouse CD48-APC antibody	Biolegend	103412
Mouse CD150-BV421 antibody	BD Biosciences	562811
Mouse CD34-BV421 antibody	BD Horizon	562608
Mouse CD16/32-PE antibody	Biolegend	101308
Mouse CD127-PE antibody	Invitrogen	2055688
Mouse Ki67-AF700 antibody	Biolegend	652420
DAPI	Beyotime	C1002
Mouse Annexin V-PE antibody	BD Pharmingen	556421
PI	BD Pharmingen	51-66211E
Mouse CD45.1-PerCP/Cyanine5.5 antibody	eBioscience	45-0453-82
Mouse CD45.2-PE/Cyanine7 antibody	Biolegend	109830
Mouse Sca-1-PE antibody	BD Biosciences	553108
Mouse B220-APC antibody	Biolegend	103212
Mouse Ly-6G/Ly-6C (Gr-1)-APC/Cyanine7 antibody	Biolegend	108424
Mouse CD11b-APC antibody	Biolegend	101212
Mouse Ter119-Brilliant Violet 510™ antibody	Biolegend	116237
CellROX™ Deep Red Flow Cytometry Assay Kit	Thermo	C10491
BD Cytofix/Cytoperm™ Fixation/Permeabilization Kit	BD Biosciences	554714
BD GolgiStop™ Protein Transport Inhibitor	BD Biosciences	554724
Mouse IL-6 PE antibody	Biolegend	504503
Mouse CCL5 PE/Cyanine7 antibody	Biolegend	149105
Mouse IFN-γ BV421 antibody	BD Horizon™	563376
Mouse TNF-α Brilliant Violet 510™ antibody	Biolegend	506339
Becn1 antibody	CST	3738S
Becn1 antibody (DyLight 488)	Novus	NB500-249G
Becn1 antibody	Santa Cruz Biotechnology	Sc-48341
GAPDH antibody	Proteintech	60004-1
ISG15 antibody	CST	2743

p-tyr705-STAT3 antibody	Abcam	ab76315
p-tyr705-STAT3 antibody	CST	9145T
p-Ser727-STAT3 antibody	Abcam	ab86430
STAT3 antibody	CST	9132S
Histone H3 antibody	CST	4499S
Myc antibody	CST	2276S
Hoechst 33342	Thermo Fisher Scientific	H3570
Atg7 antibody	Abcam	ab133528
Atg5 antibody	HUABIO	ET1611-38
FLAG antibody	OriGene	TA50011-100
Mouse LC3 antibody	Novus	NB100-2220
Mouse Lamp1 antibody	Abcam	ab208943
LC3A/B (D3U4C) XP® Rabbit mAb (Alexa Fluor® 594 Conjugate)	CST	14079s
Goat anti-rabbit IgG (H+L), HRP conjugate	CST	7074S
Goat anti-mouse IgG (H+L), HRP conjugate	Proteintech	SA00001-1
DyLight649 goat anti-rabbit IgG [H+L]	Multi Sciences	GAR6492
Pierce Anti-c-Myc Magnetic Beads	Thermo Fisher Scientific	88842
Anti-DYKDDDDK G1 Affinity Resin	Genscript	L00432
pCMV6-Entry	Origene	PS100001
Stat3 (Myc-DDK-tagged)-pCMV6	Origene	MR227265
Becn1 (Myc-DDK-tagged)-pCMV6	Origene	MR207162
Stattic	Selleckchem	S7024
Poly (I:C)	Sigma	P0913
MethoCult^{TM} GF M3434	STEMCELLTechnologies	03434
Paraformaldehyde	Sinopharm Chemical Reagent	8009628
Saponin	Sigma	S7900
Fibronectin	STEMCELLTechnologies	07159
NE-PER Nuclear and Cytoplasmic Extraction Reagents	Thermo Fisher Scientific	78835
Protein A/G agarose	Beyotime Biotechnology	P2012

Supplementary Table 2. Primers for quantitative PCR detection of gene expression

**Primer name (Mouse)**	**Sequence (5'-3')**
Becn1-F	CGTGGAGAAAGGCAAGATTGAAGA
Becn1-R	GTGAGGACACCCAAGCAAGACC
Bst2-F	TGTTCGGGGTTACCTTAGTCA
Bst2-R	GCAGGAGTTTGCCTGTGTCT
Ccnd1-F	TGACTGCCGAGAAGTTGTGC
Ccnd1-R	CTCATCCGCCTCTGGCATT
Ccnd2-F	TGAATTACCTGGACCGTTTCTTG
Ccnd2-R	AGAGTTGTCGGTGTAAATGCAC
Ccnd3-F	TGGATCGCTACCTGTCCTG
Ccnd3-R	CCTGGTCCGTATAGATGCAAAG
Ccna2-F	TGGATGGCAGTTTTGAATCACC
Ccna2-R	CCCTAAGGTACGTGTGAATGTC
Ccne1-F	GAAAAGCGAGGATAGCAGTCAG
Ccne1-R	CCCAATTCAAGACGGGAAGTG
Ccne2-F	ATGTCAAGACGCAGCCGTTTA
Ccne2-R	GCTGATTCCTCCAGACAGTACA
Cxcl10-F	CCAAGTGCTGCCGTCATTTTC
Cxcl10-R	TCCCTATGGCCCTCATTCTCA
Ccl5-F	GCTGCTTTGCCTACCTCTCC
Ccl5-R	TCGAGTGACAAACACGACTGC
Ddx58-F	AAGAGCCAGAGTGTCAGAATCT
Ddx58-R	AGCTCCAGTTGGTAATTTCTTGG
Gbp2-F	TGGGGTAGACGATTCCGCTAA
Gbp2-R	AGAAGTGACGGGTTTTCCGTT
Gbp7-F	AACTGAGGGTGAACTCCAAAGC
Gbp7-R	GTTTCAGACCTAACTGTGGTGC
Gapdh-F	AGCTTGTCATCAACGGGAAG
Gapdh-R	TTTGATGTTAGTGGGGTCTCG
Herc6-F	AATTGGTGGCCGTGTTTCAC
Herc6-R	CCTGATGAGTTGGGTTGCTTG
Irf1-F	ATGCCAATCACTCGAATGCG
Irf1-R	CCTGCTTTGTATCGGCCTGT

Ifitm1-F	GACAGCCACCACAATCAACAT
Ifitm1-R	CCCAGGCAGCAGAAGTTCAT
Ifitm3-F	CCCCCAAACTACGAAAGAATCA
Ifitm3-R	ACCATCTTCCGATCCCTAGAC
Irgm1-F	GGTCAGTAGGAGCACCGAAAG
Irgm1-R	TGACTCGAAGTGCATTGATGAAA
Ifn-γ-F	GGCCATCAGCAACAACATAAGCGT
Ifn-γ-R	TGGGTTGTTGACCTCAAACTTGGC
Ifn-α-F	GGACTTTGGATTCCCGCAGGAGAAG
Ifn-α-R	GCTGCATCAGACAGCCTTGCAGGTC
Ifn-β-F	TGGGTGGAATGAGACTATTGTTG
Ifn-β-R	CTCCCACGTCAATCTTTCCTC
IL6-F	TAGTCCTTCCTACCCCAATTTCC
IL6-R	TTGGTCCTTAGCCACTCCTTC
Isg15-F	AGTGATGCTAGTGGTACAGAACT
Isg15-R	CAGTCTGCGTCAGAAAGACCT
Ifit1-F	GTCCGGTTAAATCCAGAAGATCC
Ifit1-R	GCTTTGTCTACGCGATGTTTCC
Ly6a-F	AGGAGGCAGCAGTTATTGTGG
Ly6a-R	CGTTGACCTTAGTACCCAGGA
Mda5-F	AGATCAACACCTGTGGTAACACC
Mda5-R	CTCTAGGGCCTCCACGAACA
Oas2-F	AAACCTCACACCCAACGAAAA
Oas2-R	CCACCCTTAGCCACTTCCT
P18-F	CCTTGGGGGAACGAGTTGG
P18-R	AAATTGGGATTAGCACCTCTGAG
P19-F	CTGAACCGCTTTGGCAAGAC
P19-R	GCCCTCTCTTATCGCCAGAT
P21-F	CGAGAACGGTGGAACTTTGAC
P21-R	CCAGGGCTCAGGTAGACCTT
P27-F	TCTCTTCGGCCCGGTCAAT
P27-R	AAATTCCACTTGCGCTGACTC
P57-F	TGAACGCCGAGGACCAGAAC
P57-R	TGCACCGTCTCGCGGTAGA

Parp9-F	CGAAAGACCAGCCAACAATTCT
Parp9-R	AGGTCATCCTTCCAGACAGATAA
Stat1-F	GCTGCCTATGATGTCTCGTTT
Stat1-R	TGCTTTTCCGTATGTTGTGCT
Stat3-F	CAATACCATTGACCTGCCGAT
Stat3-R	GAGCGACTCAAACTGCCCT
Tgtp1-F	TGCACAGATGGGGATGAATTTC
Tgtp1-R	TCACTGTCGAGAGACTCCTGA
Tnf-F	CCTGTAGCCCACGTCGTAG
Tnf-R	GGGAGTAGACAAGGTACAACCC
Ubch8-F	GACGATGCCAATGTGCTTGTG
Ubch8-R	CTGGGGAAATCAATCCGCACT
Usp18-F	AGAGTTAGCAAGCTCCGACAT
Usp18-R	TGAGGTGAATGGTCAAGGTTTG

Supplementary Table 3. The intersected differential gene list by cluster analysis in Figure S9A

Gene ID	Gene	Log_2(Foldchange)	q-value
18788	Serpinb2	7.077334878	6.66E-18
17339	Mip	6.15533739	8.29E-11
58203	Zbp1	5.321168649	0
240327	Gm4951	4.928050679	0
626578	Gbp10	4.881097842	0
15945	Cxcl10	4.871389665	0
60440	Iigp1	4.549022718	0
99899	Ifi44	4.515137901	0
21822	Tgtp1	4.500140977	0
219132	Phf11d	4.485621177	1.64E-166
15957	Ifit1	4.25426799	0
24110	Usp18	4.146852974	0
58185	Rsad2	3.840604083	0
14120	Fbp2	3.714764799	0.00234483
15486	Hsd17b2	3.714764799	0.00234483
100702	Gbp6	3.610120069	0
15959	Ifit3	3.471379604	0
100038882	Isg15	3.439551265	0
14544	Gda	3.418608323	2.13E-222
54123	Irf7	3.412333912	0
16560	Kif1a	3.229337972	0.007999249
17882	Myh2	3.229337972	0.007999249
57444	Isg20	3.185614167	0
170442	Bbox1	3.077334878	4.69E-05
13649	Egfr	3.077334878	1.41E-06
23972	Papss2	3.064951154	2.46E-97
15951	Ifi204	3.05018856	0
22169	Cmpk2	3.038988352	0
246728	Oas2	2.972462359	0
53857	Tuba8	2.947787582	0
23962	Oasl2	2.934305499	0

78906	Misp	2.907409877	1.15E-09
628705	Phf11c	2.903785776	1.49E-131
17857	Mx1	2.7540905	0
433182	Eno1b	2.744614727	5.38E-06
14469	Gbp2	2.738100263	0
246727	Oas3	2.665833662	0
246730	Oas1a	2.617107726	0
12035	Bcat1	2.608810111	0
22152	Tubb3	2.600564377	2.63E-13
17858	Mx2	2.580953597	2.22E-158
20846	Stat1	2.488792825	0
16145	Igtp	2.483178501	0
327978	Slfn5	2.474964014	0
234311	Ddx60	2.438699195	0
71586	Ifih1	2.401771436	0
668940	Myh7b	2.366841496	0.002212482
209086	Samd9l	2.359183511	0
11777	Ap3s1	2.356965437	0
236573	Gbp9	2.323166869	0
17472	Gbp4	2.277832789	0
74558	Gvin1	2.263184148	0
15944	Irgm1	2.250399781	0
229898	Gbp5	2.153807761	9.80E-81
55932	Gbp3	2.140260944	0
193740	Hspa1a	2.136674137	2.57E-64
219131	Phf11a	2.098062962	3.78E-190
229900	Gbp7	2.070056239	0
11745	Anxa3	2.062816824	0
327959	Xaf1	2.036847404	0
80861	Dhx58	1.969329253	0
56791	Ube2l6	1.927582605	0
22436	Xdh	1.911855782	3.56E-33
15958	Ifit2	1.909321998	0
80285	Parp9	1.821856587	0

23961	Oas1b	1.820176359	2.50E-79
67138	Herc6	1.758600244	0
107869	Cth	1.745658807	4.49E-22
19039	Lgals3bp	1.736614534	0
21354	Tap1	1.725813412	0
97122	Hist2h4	1.695905772	2.68E-08
15953	Ifi47	1.663619225	0
231712	Trafd1	1.661764496	0
20210	Saa3	1.644375471	6.56E-10
27053	Asns	1.508874397	0
230073	Ddx58	1.508570381	0
72747	Ttc39c	1.499866914	2.93E-15
100048534	Cfap43	1.492372378	0.015910044
22042	Tfrc	1.490420761	0
14828	Hspa5	1.479339291	0
59027	Nampt	1.468490151	0
243771	Parp12	1.375445371	4.92E-279
26433	Plod3	1.365537626	4.93E-190
56421	Pfkp	1.328945176	0
547253	Parp14	1.29552106	0
20135	Rrm2	1.275939386	0
66222	Serpinb1a	1.274435991	0
241639	Fermt1	1.229337972	0.021274101
109731	Maob	1.229337972	7.95E-05
331026	Gmppb	1.220402075	4.45E-109
14933	Gk	1.194760151	8.65E-190
16912	Psmb9	1.17876736	0
67078	Pgp	1.119565263	1.16E-287
17886	Myh9	1.111786565	4.40E-295
55993	Msh4	1.091834448	0.005186274
11997	Akr1b7	1.077334878	0.031437363
78781	Zc3hav1	1.066104051	2.75E-300
100121	Tdrd7	1.057281958	8.02E-73
53421	Sec61a1	1.040118797	0

70110	Ifi35	1.021181629	0
21356	Tapbp	1.008810279	0
192185	Nadk	1.008537314	0
12012	Baat	1.00694555	0.018861558
15505	Hsph1	1.004018962	0
20847	Stat2	1.002584925	3.21E-134
105445	Dock9	-1.007531407	1.41E-91
17187	Max	-1.016314356	4.18E-126
11772	Ap2a2	-1.016518952	0
13043	Cttn	-1.037961466	8.29E-122
56794	Hacl1	-1.044734123	1.22E-95
22596	Xrcc5	-1.044888124	0
73389	Hbp1	-1.058353356	0
14645	Glul	-1.068187874	0
77559	Agl	-1.068189855	4.86E-75
14447	Gapdhs	-1.088268378	1.18E-06
14468	Gbp2b	-1.092590123	0.019580517
319157	Hist1h4f	-1.130725252	0.022327705
70536	Qpct	-1.148858343	6.24E-11
215814	Ccdc28a	-1.152214106	1.28E-49
12336	Capns1	-1.153815369	3.79E-260
17125	Smad1	-1.155213729	1.43E-98
102866	Pls3	-1.157530906	2.50E-127
104776	Aldh6a1	-1.158221805	3.05E-264
16691	Krt8	-1.168357631	9.47E-13
67198	Spats2l	-1.176248052	3.24E-09
68617	Mtcl1	-1.192125797	0.010442094
234683	Elmo3	-1.200966284	8.40E-18
22004	Tpm2	-1.209443564	5.11E-138
264895	Acsf2	-1.21123462	4.43E-10
26457	Slc27a1	-1.229313548	2.38E-63
21872	Tjp1	-1.239347952	2.13E-114
11747	Anxa5	-1.252404624	0
70676	Gulp1	-1.256652886	5.01E-36

22608	Ybx1	-1.267563042	0
12709	Ckb	-1.293485844	7.25E-171
94275	Maged1	-1.312047067	0
65970	Lima1	-1.318724946	5.16E-48
19383	Raly	-1.373616196	0
107747	Aldh1l1	-1.445379182	6.77E-41
74637	Shpk	-1.464558901	8.85E-30
80884	Maged2	-1.466223235	0
75578	Fggy	-1.466907862	7.69E-146
70789	Kynu	-1.489107534	1.26E-20
21336	Tacr1	-1.609865816	0.006950853
17920	Myo6	-1.618367817	6.31E-236
14085	Fah	-1.618473391	2.78E-27
20533	Slc4a1	-1.682605852	1.40E-26
50997	Mpp2	-1.716026772	3.53E-12
217866	Cdc42bpb	-1.74466682	2.90E-65
235406	Snx33	-1.751033221	8.60E-55
13511	Dsg2	-1.776116458	2.47E-70
17436	Me1	-1.810819155	8.08E-12
110877	Slc18a1	-1.833950939	0
66395	Ahnak	-1.843602426	3.99E-258
64705	Dpys	-1.847477625	2.21E-07
16832	Ldhb	-1.848172398	0
67815	Sec14l2	-1.899945045	0.000361213
73162	Otud3	-1.925836578	3.60E-28
80281	Cttnbp2nl	-1.938556494	1.33E-31
16562	Kif1c	-1.957660543	6.87E-13
17912	Myo1b	-2.046047537	1.54E-137
12406	Serpinh1	-2.049840476	7.97E-239
12826	Col4a1	-2.110364825	0
103988	Gck	-2.1405246	3.90E-74
228140	Tnks1bp1	-2.14277588	2.68E-57
76263	Gstk1	-2.144333698	1.80E-219
13636	Efna1	-2.176233783	3.23E-183

78070	Cpt1c	-2.219702041	2.07E-12
13132	Dab2	-2.230004786	0
226695	Ifi205	-2.2523035	3.64E-61
19428	Rasl2-9	-2.262515125	1.67E-05
17755	Map1b	-2.316670895	0
13101	Cyp2d10	-2.412593341	0.000436281
13074	Cyp17a1	-2.414518218	0.012928297
193034	Trpv1	-2.414518218	0.012928297
140792	Colec12	-2.470199378	1.01E-163
12116	Bhmt	-2.52554953	6.47E-10
109624	Cald1	-2.533542321	3.20E-80
94242	Tinagl1	-2.589734745	0
170676	Peg10	-2.601603771	3.70E-13
69903	Rasip1	-2.646159202	5.71E-242
14261	Fmo1	-2.670666238	4.65E-34
56188	Fxyd1	-2.745724126	8.16E-33
57342	Parva	-2.767189836	1.76E-28
14863	Gstm2	-2.779432238	3.74E-44
57440	Ehd3	-2.810074015	0
226101	Myof	-2.823142433	5.55E-135
259300	Ehd2	-2.861977195	1.74E-14
50884	Nckap1	-2.876120182	6.55E-180
403174	Msantd1	-2.988754312	1.19E-13
13849	Ephx1	-3.031189578	5.61E-54
11464	Actc1	-3.311758644	3.57E-30
218952	Fermt2	-3.325028431	4.07E-246
23859	Dlg2	-3.390769615	1.75E-186
69675	Pxdn	-3.483701783	6.84E-236
12389	Cav1	-3.507627622	1.11E-33
15233	Hgd	-3.552021742	1.02E-08
58242	Nudt11	-3.553401651	9.35E-44
18534	Pck1	-3.677552624	0.000162804
26358	Aldh1a7	-3.728178697	4.00E-20
230837	Asap3	-3.793029841	1.46E-07

77569	Limch1	-3.935864619	2.90E-54
74174	Gtsf1	-4.092590123	0.000874279
68802	Mypn	-4.092590123	0.000874279
18113	Nnmt	-4.092590123	0.000874279
22061	Trp63	-4.092590123	1.33E-05
103968	Plin1	-4.262515125	0.000461483
230163	Aldob	-4.579089985	1.85E-126
208943	Myo5c	-4.639078476	0
380928	Lmo7	-4.697452181	2.90E-19
15445	Hpd	-4.899945045	2.04E-05
57435	Plin4	-5.092590123	6.04E-06
209186	Acnat2	-5.180052964	3.32E-06
19193	Pipox	-5.552021742	1.75E-07
15112	Hao1	-5.736446313	3.12E-08
93732	Acox2	-5.999480719	1.89E-09
237320	Aldh8a1	-6.616152079	3.87E-24
14859	Gsta3	-6.950571118	2.22E-29
103149	Upb1	-7.158679314	3.49E-33
22262	Uox	-8.414518218	2.84E-35
14115	Fbln2	-8.560195673	3.40E-38
