# Supplementary material for: Beclin 1 prevents ISG15-mediated cytokine storms to secure fetal hematopoiesis and survival
Source: J Clin Invest. 2025 Feb 3;135(3):e177375. doi: 10.1172/JCI177375 (PMC11785930; doi:10.1172/JCI177375)
Supplement: Unedited blot and gel images [file jci-135-177375-s217.pdf]

Unedited gel for Figure 1B, left panel

Note book3, P35

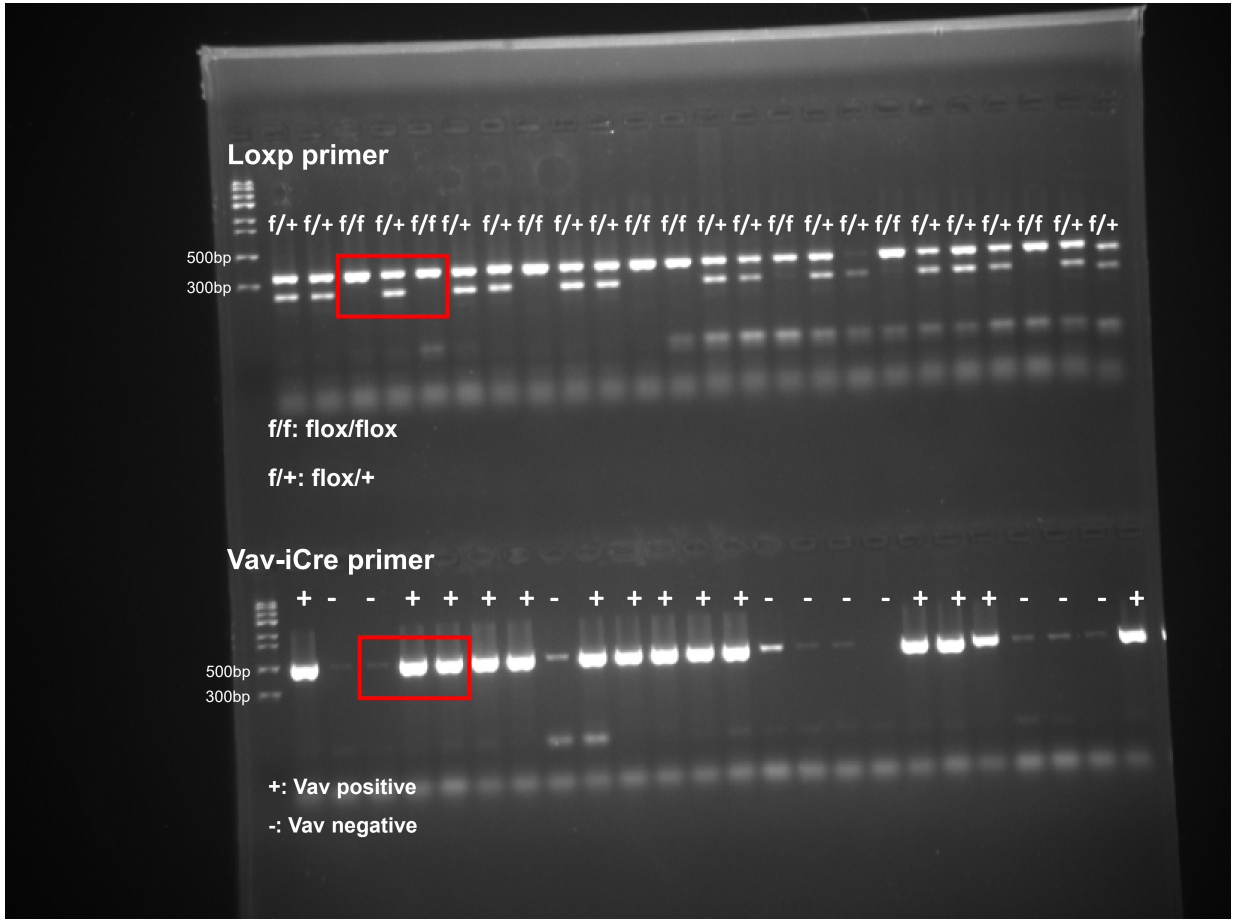

Jul 8,2020

Unedited blots for Figure 1B, right panel

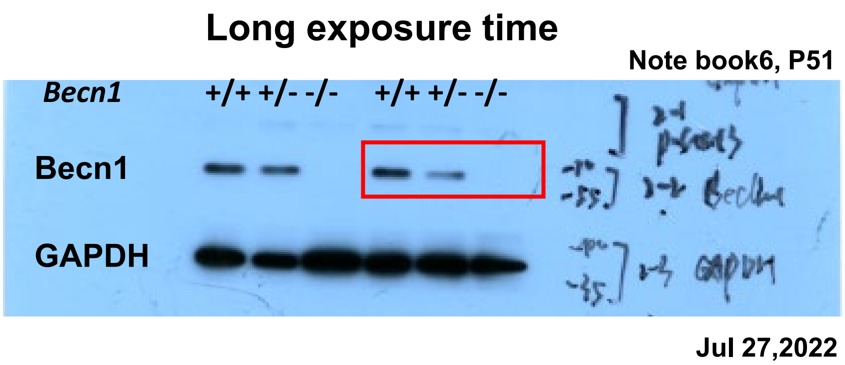

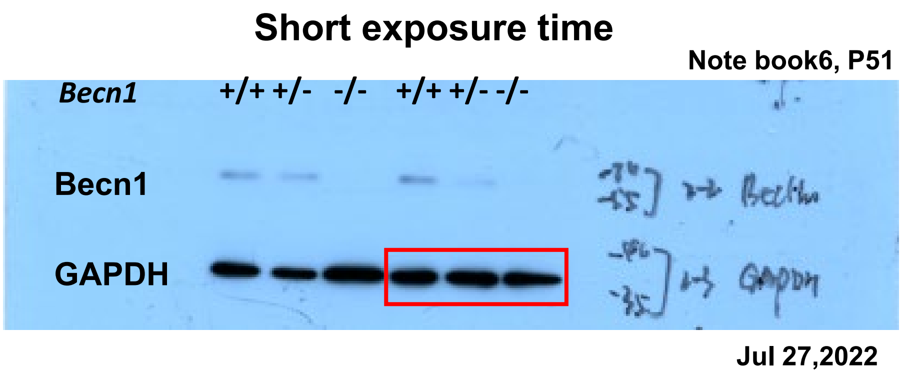

Unedited blots for Figure 5B

Note book5, P9

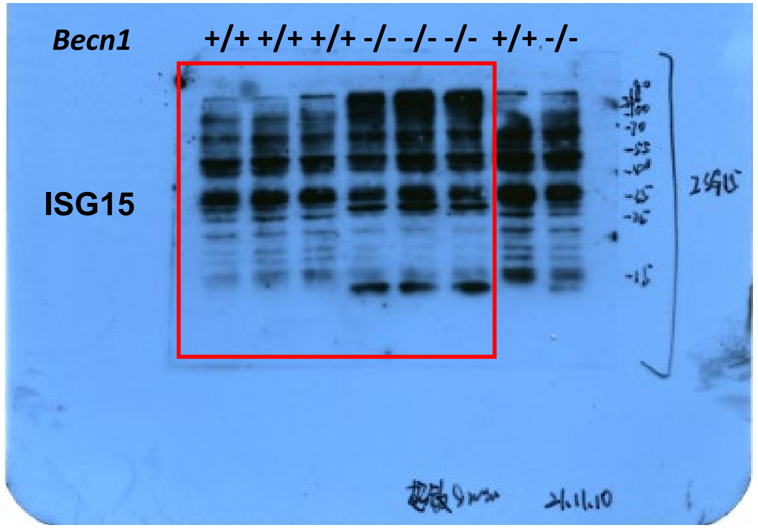

Nov 10,2021

Long exposure time

Note book5, P10

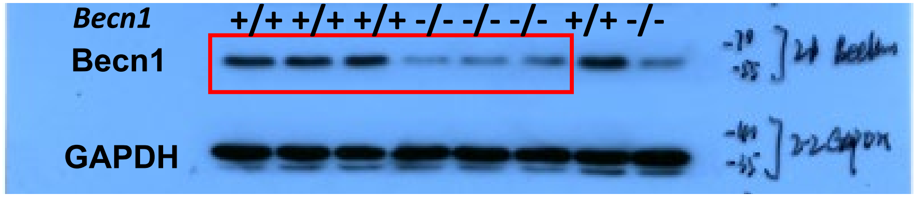

Nov 11,2021

Short exposure time

Note book5, P10

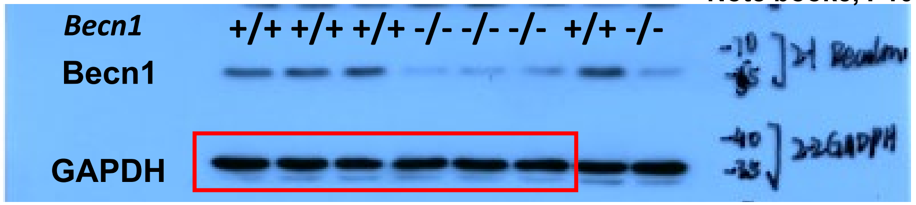

Nov 11,2021

The right blots were reprobed from the left blot

Unedited blots for Figure 6B

Note book6, P136

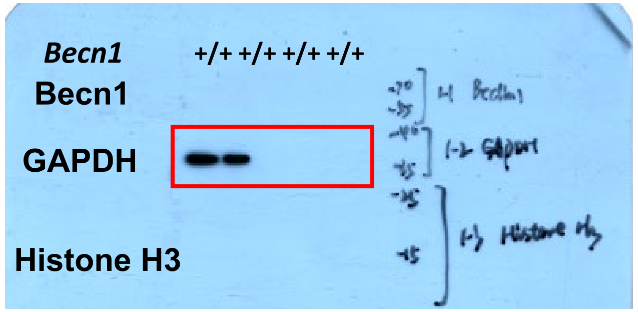

Mar 22,2023

Short exposure time

Note book6, P136

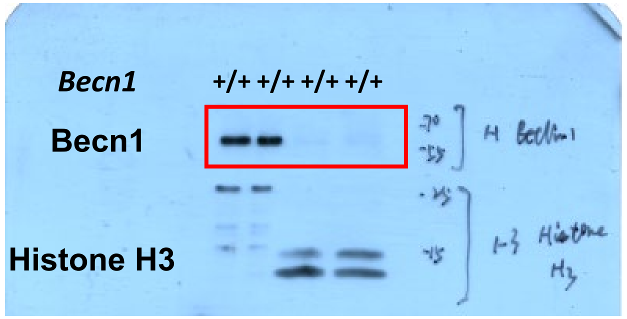

Mar 22,2023

Long exposure time

Note book6, P136

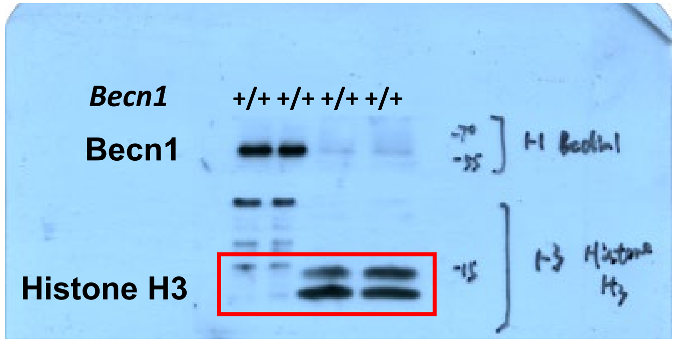

Mar 22,2023

The middle and right films were from the same blot with different exposure time

Unedited blots for Figure 6D (Left panel)

Shorter exposure time

Note book4, P143

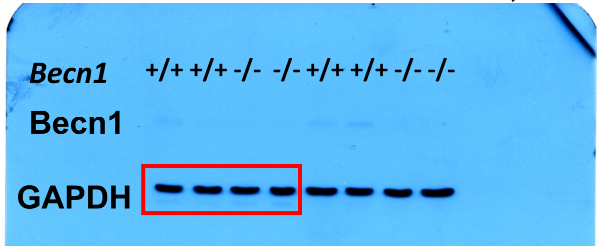

Oct 30,2021

Short exposure time

Note book4, P143

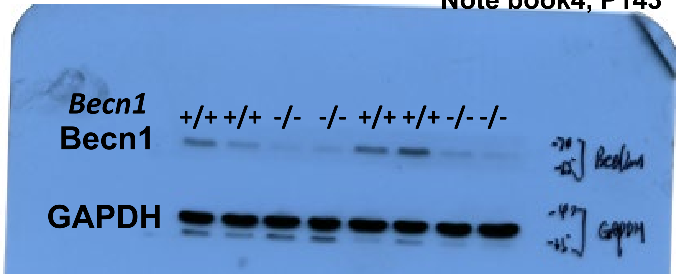

Oct 30,2021

Long exposure time

Note book4, P143

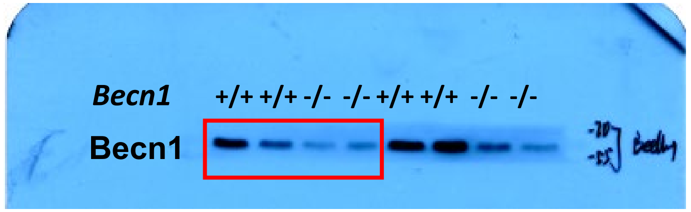

Oct 30,2021

Shorter exposure time

Note book5, P45

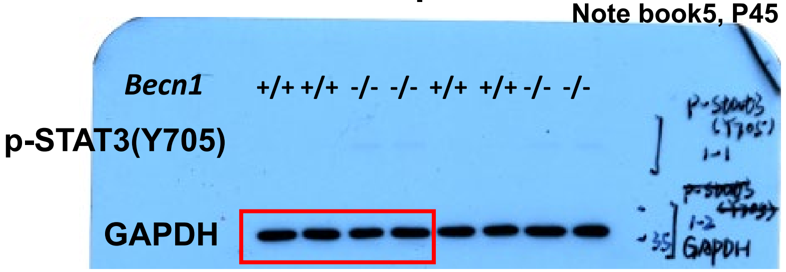

Dec 26,2021

Short exposure time

Note book5, P45

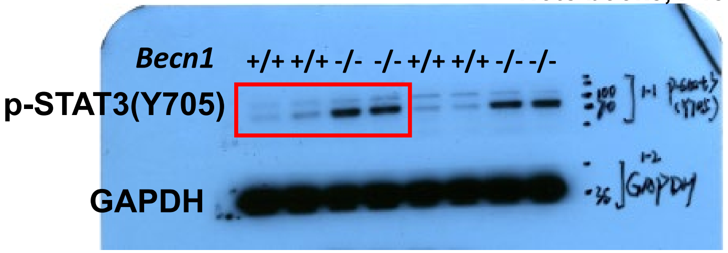

Dec 26,2021

Note book5, P46

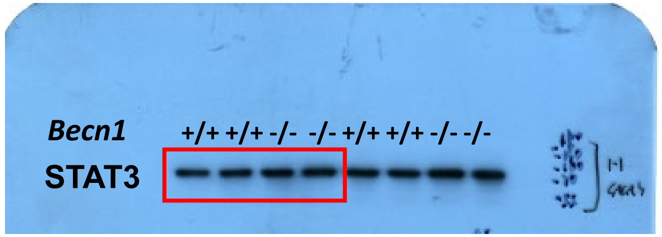

Dec 27,2021

The blot of STAT3 was reprobed from the blot of p-STAT3(Y705)

Unedited blots for Figure 6D (Right panel)

Shorter exposure time

Note book5, P45

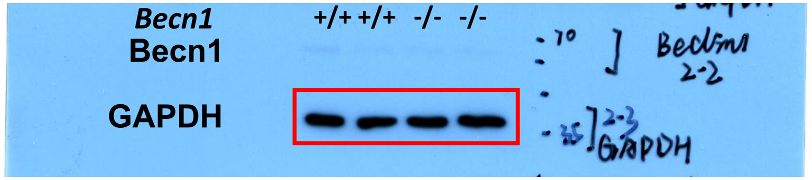

Dec 26,2021

Short exposure time

Note book5, P45

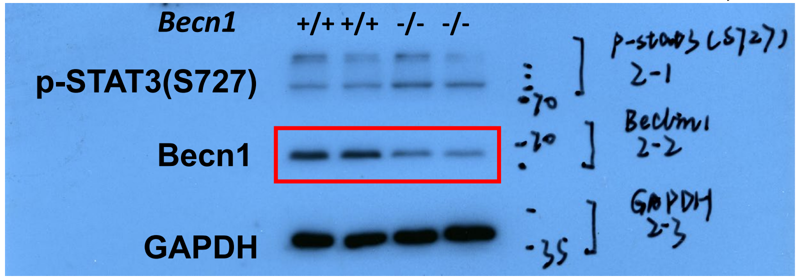

Dec 26,2021

Long exposure time

Note book5, P45

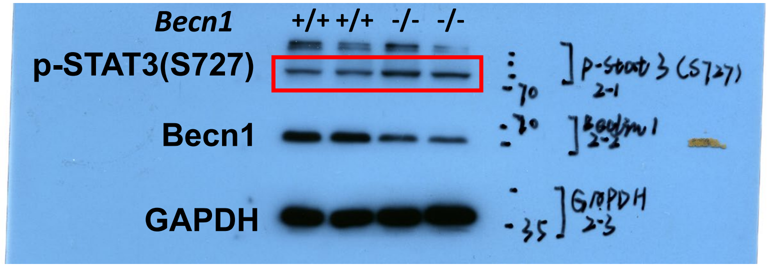

Dec 26,2021

Note book5, P46

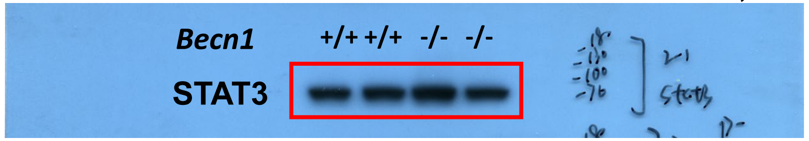

Dec 27,2021

The blot of STAT3 was reprobed from the blot of p-STAT3(S727)

Unedited blots for Figure 6F

Short exposure time

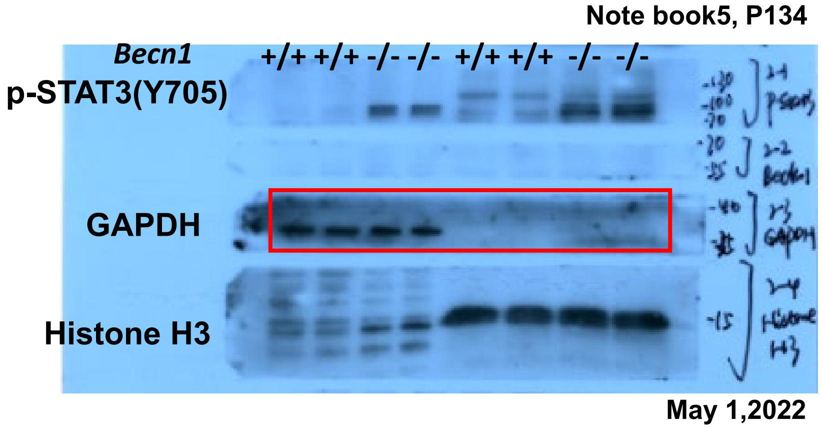

Long exposure time

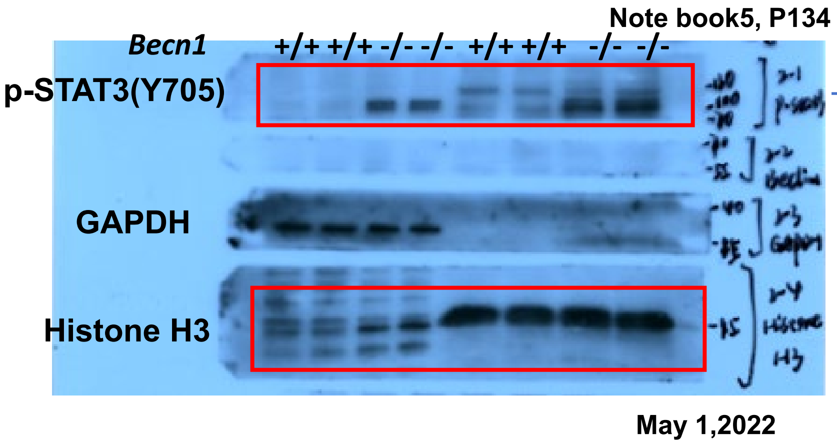

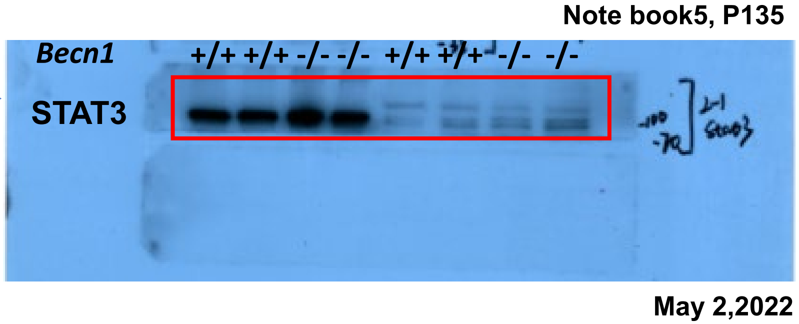

The blot of STAT3 was reprobed from the blot of p-STAT3(Y705)

Unedited blots for Figure 8B

Long exposure time

Note book6, P6

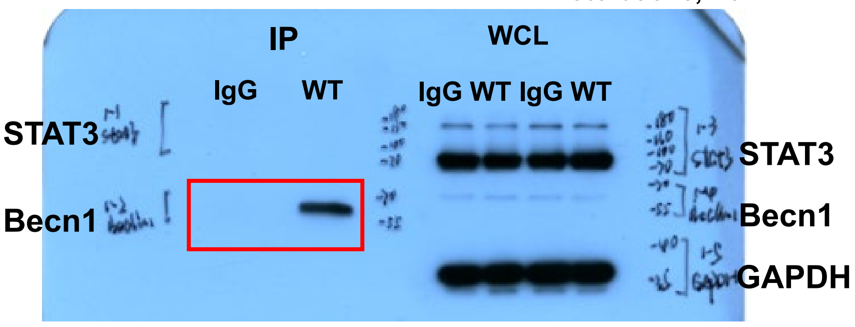

May 26,2022

Short exposure time

Note book6, P6

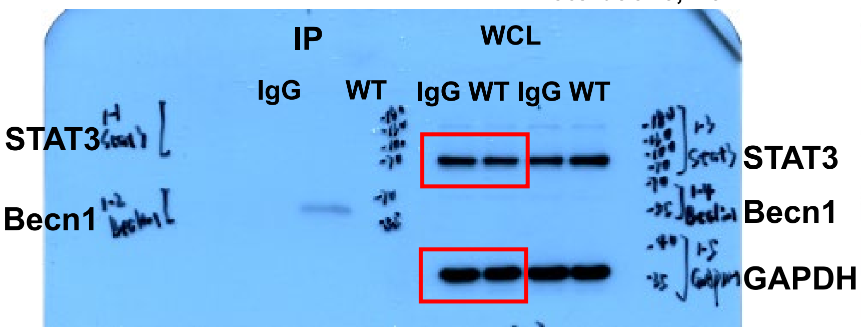

May 26,2022

Shorter exposure time

Note book6, P6

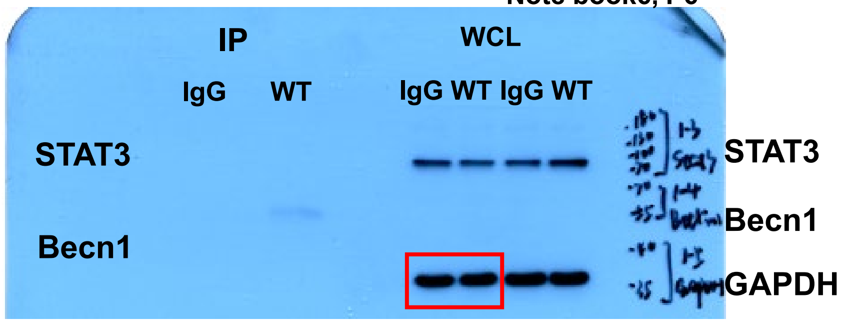

May 26,2022

Longer exposure time

Note book6, P6

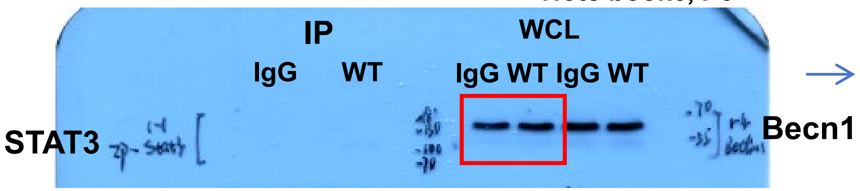

May 26,2022

Note book6, P7

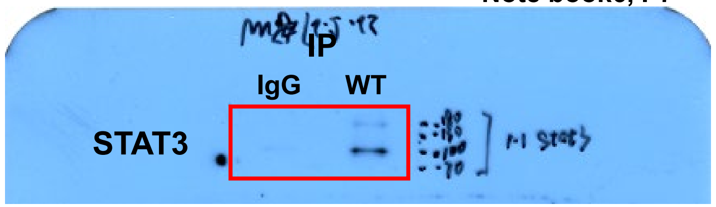

May 27,2022

The right IP-STAT3 blot was reprobed from the left IP-STAT3 blot

Unedited blots for Figure 8D

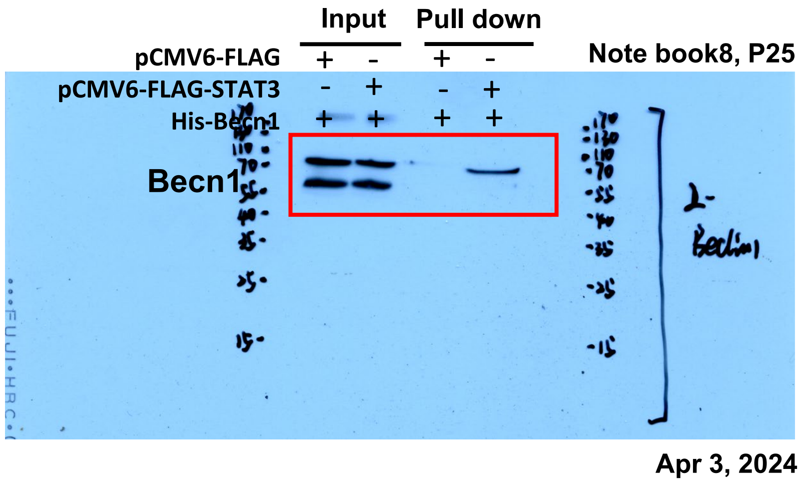

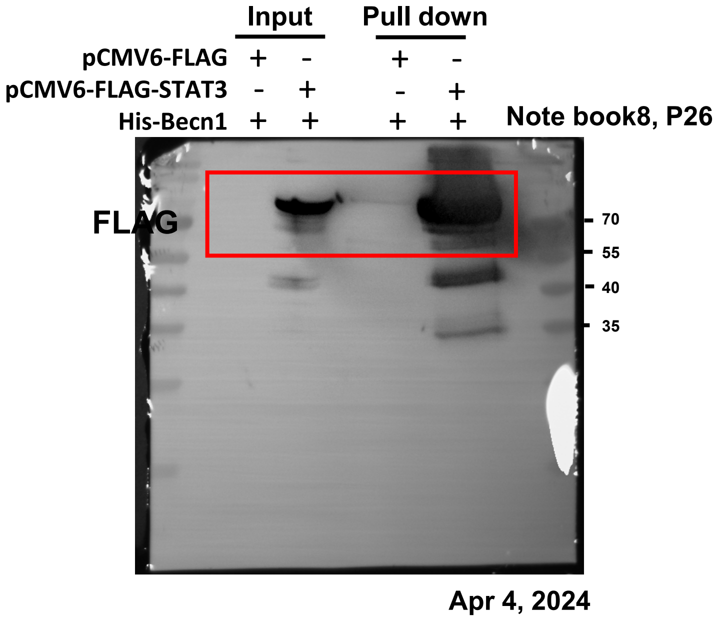

The right FLAG blot was reprobed from the left Becn1 blot

Unedited blots for Figure 8I

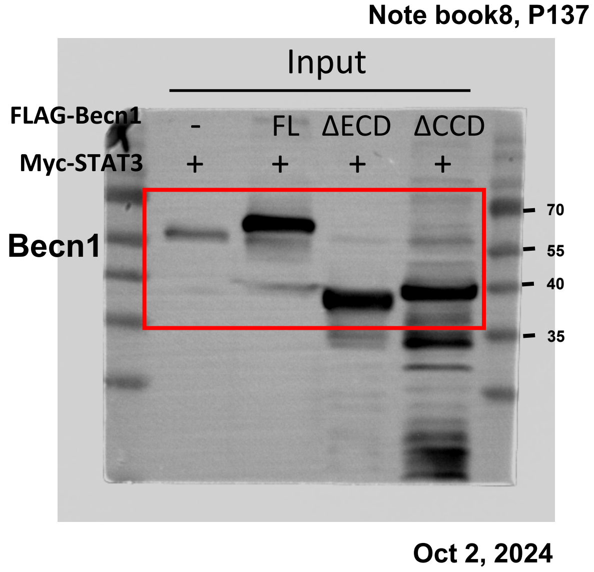

Short exposure time

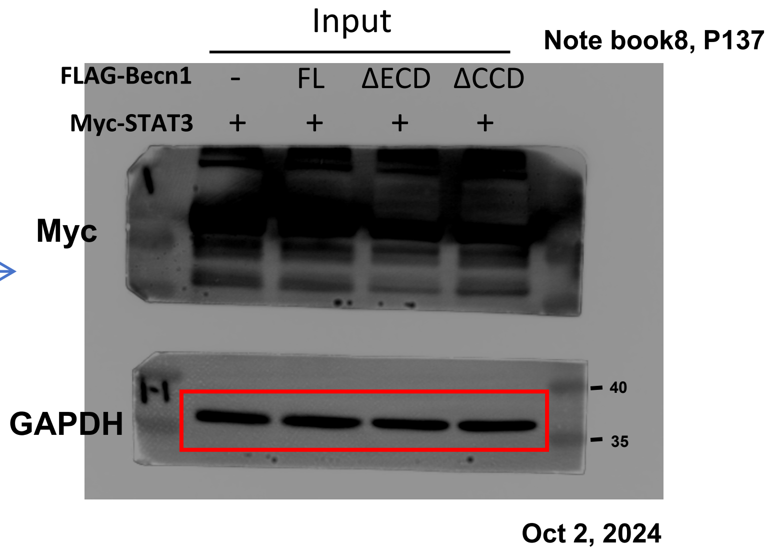

Shorter exposure time

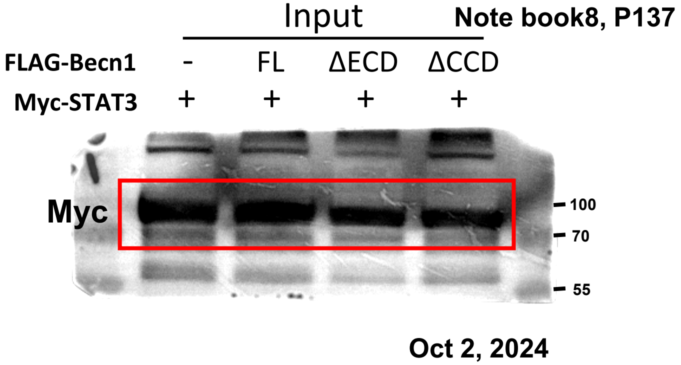

The right GAPDH and Myc blots were reprobed from the left Becn1 blot

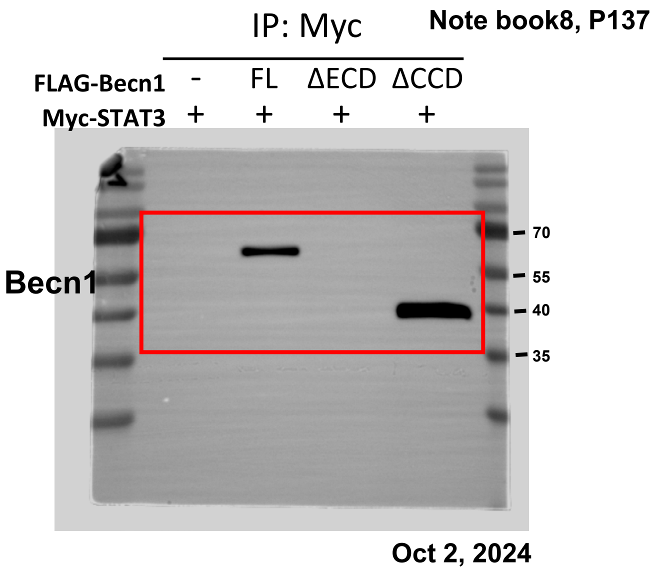

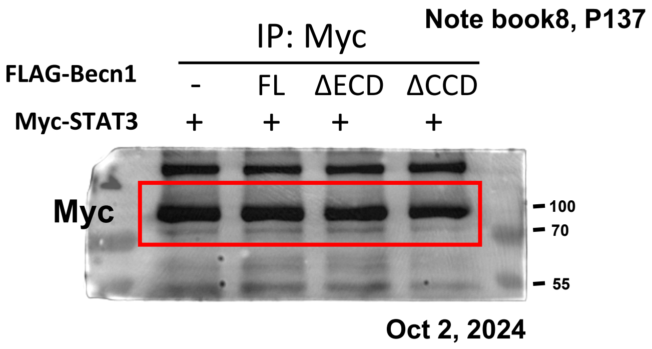

The right Myc blot was reprobed from the left Becn1 blot

Unedited blots for Figure 8J

Short exposure time

Note book8, P145

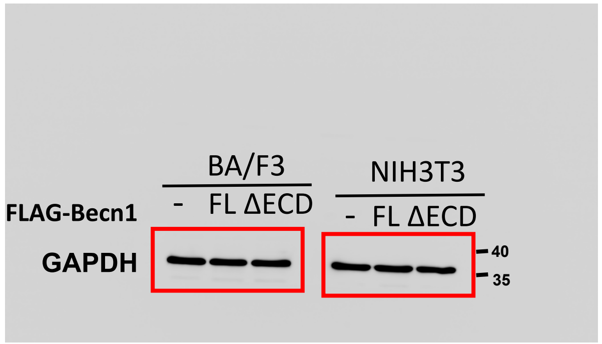

Nov 5, 2024

Long exposure time

Note book8, P145

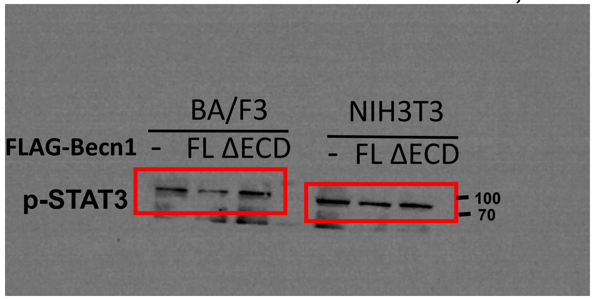

Nov 5, 2024

Note book8, P145

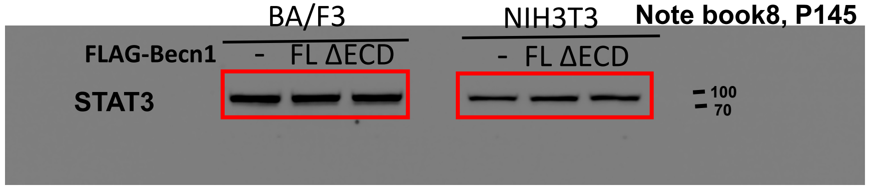

Nov 5, 2024

The STAT3 blots were reprobed from the p-STAT3 blot

Note book8, P145

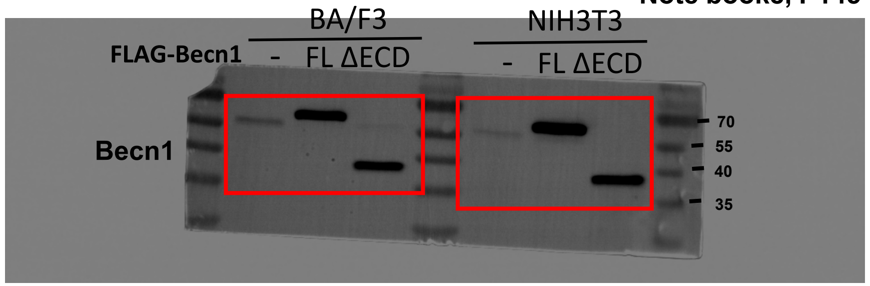

Nov 5, 2024

Note book8, P146

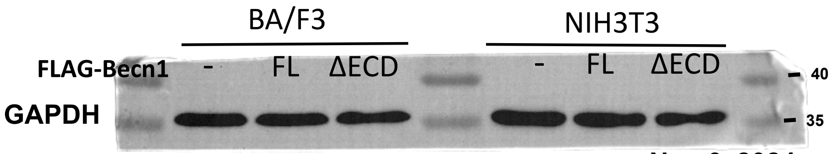

Nov 6, 2024

The GAPDH blots were reprobed from the Becn1 blot

Unedited blots for Figure 9A

Long exposure time

Note book5, P127

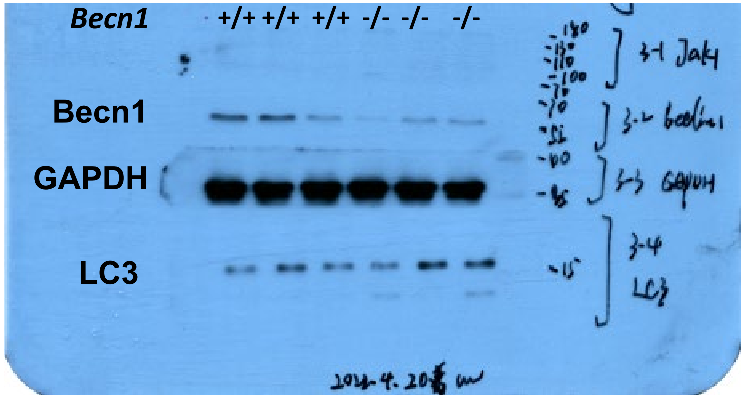

Apr 20,2022

Short exposure time

Note book5, P127

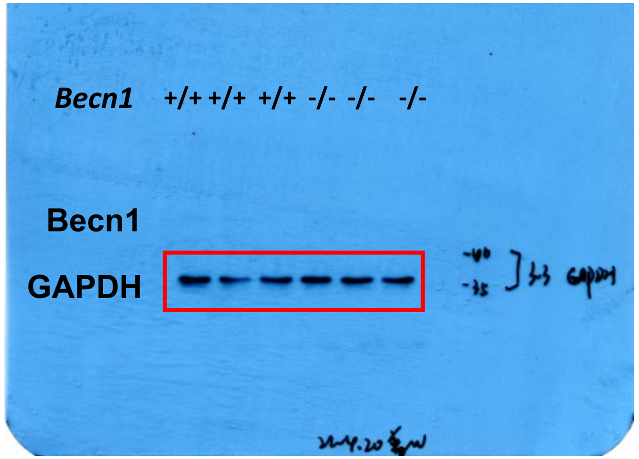

Apr 20,2022

Longer exposure time

Note book5, P127

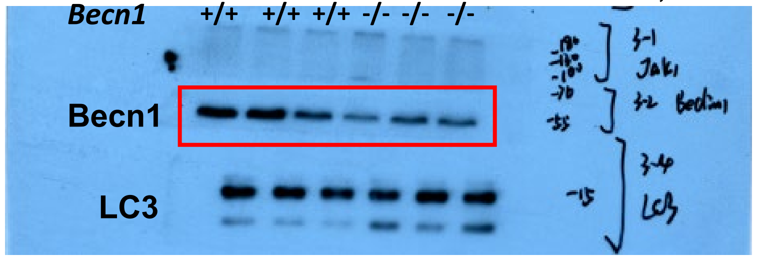

Apr 20,2022

Longer exposure time

Note book5, P127

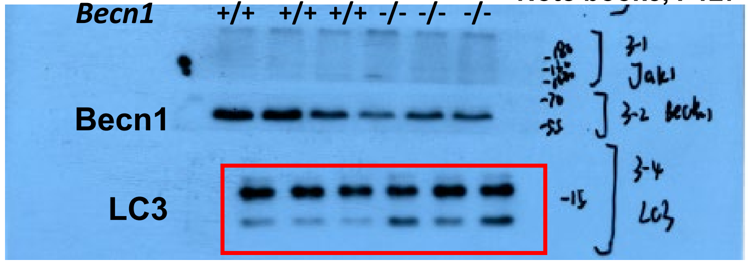

Apr 20,2022

Long exposure time

Note book6, P62

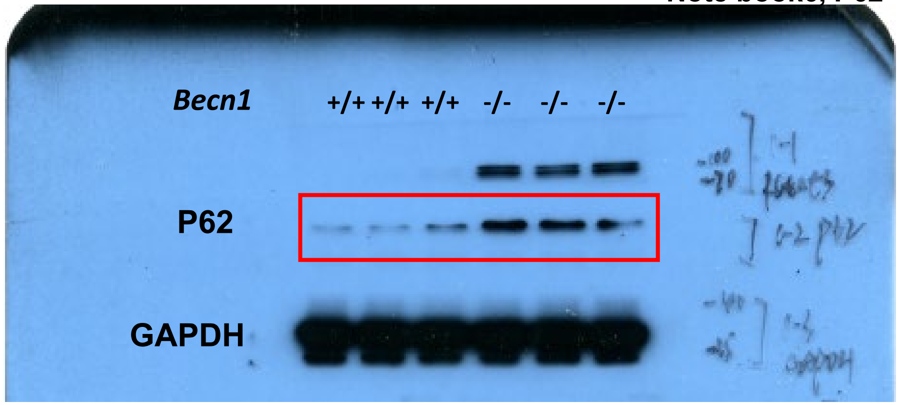

Aug 11,2022

Short exposure time

Note book6, P62

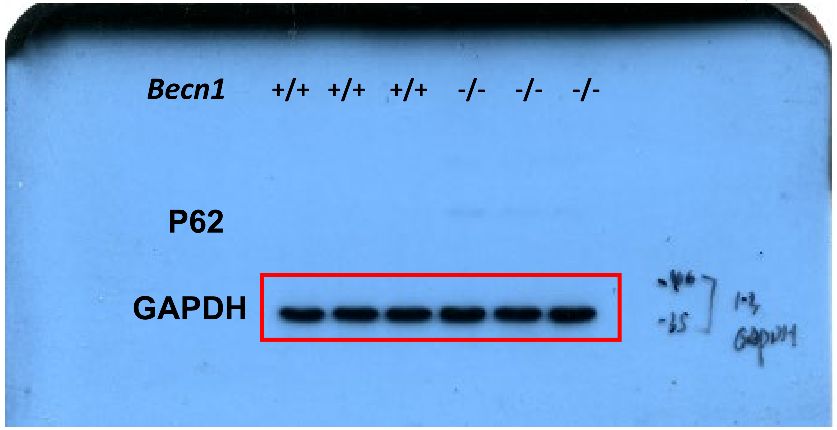

Aug 11,2022

Unedited blots for Figure 9B

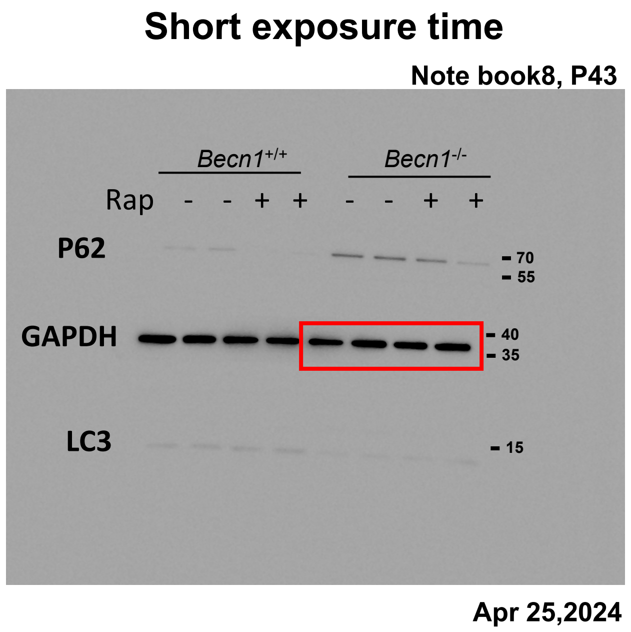

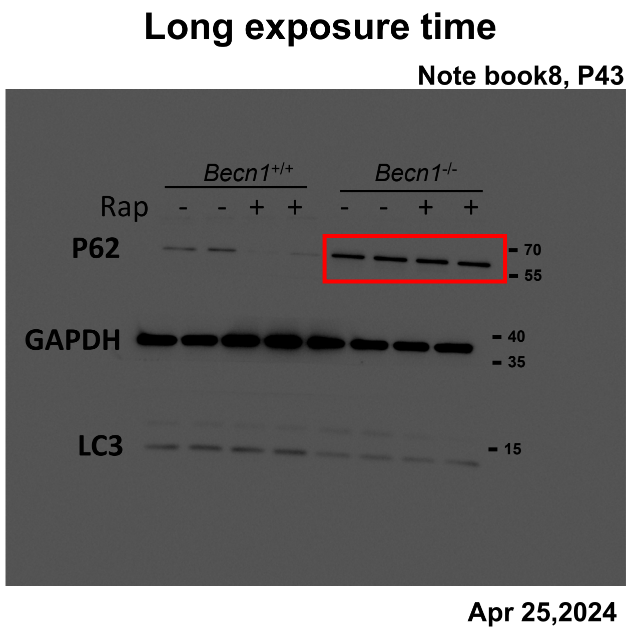

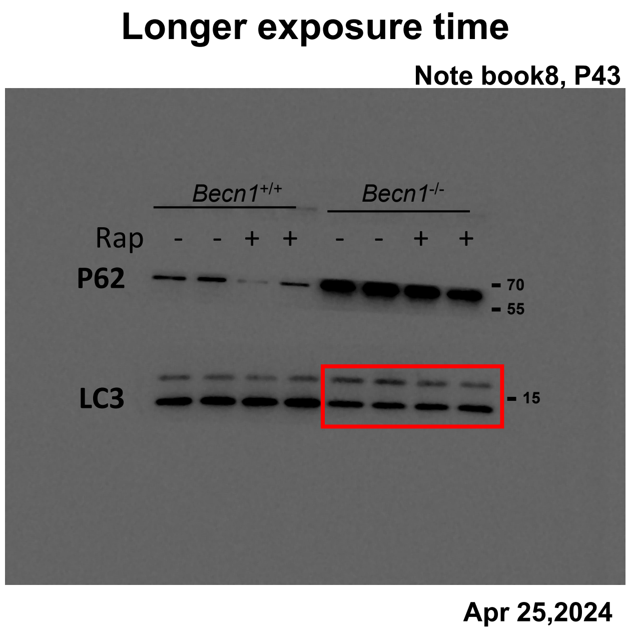

Unedited blots for Figure 9E

Long exposure time

Note book6, P65

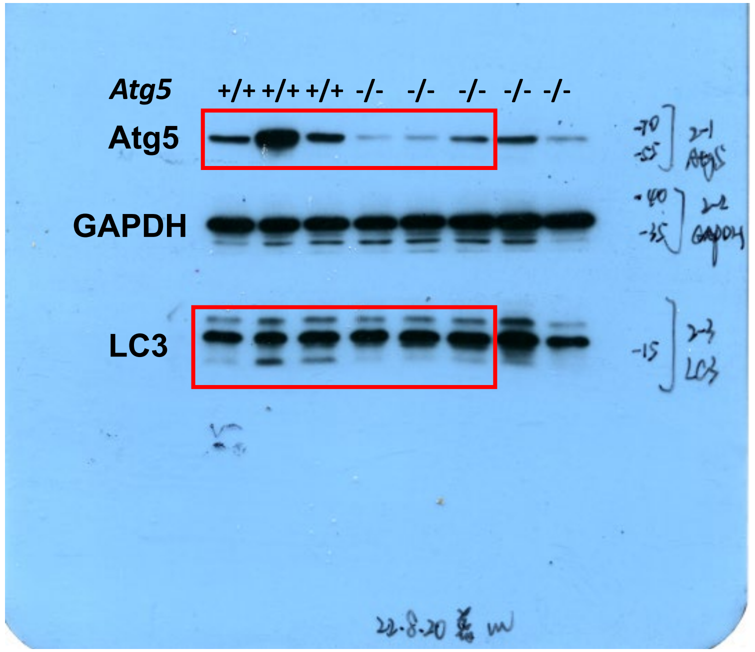

Aug 20,2022

Short exposure time

Note book6, P65

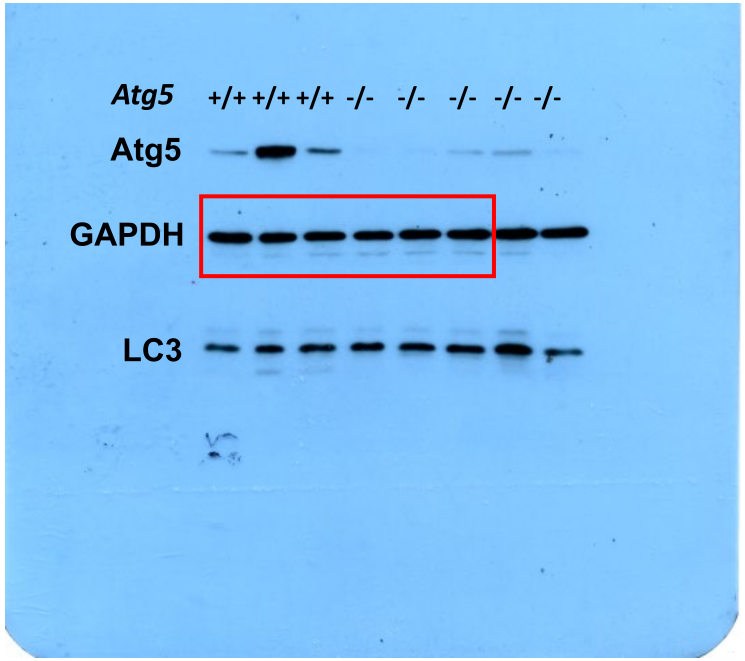

Aug 20,2022

Unedited blots for Figure 9F

Note book6, P6

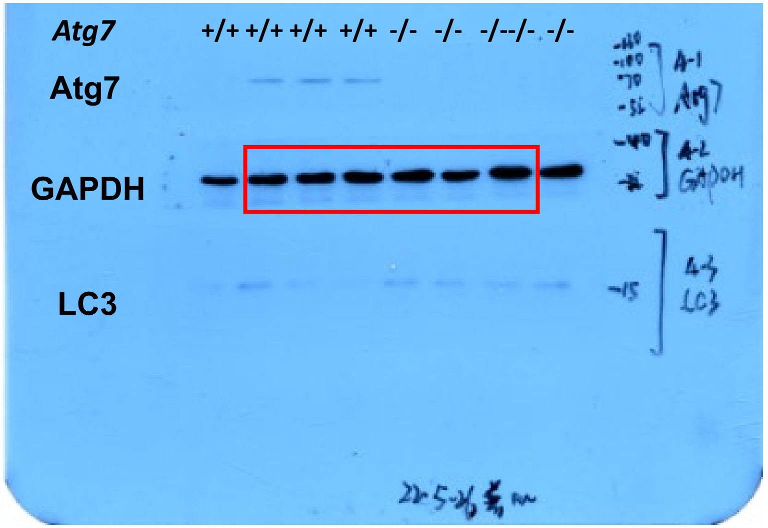

May 26,2022

Short exposure time

Note book6, P6

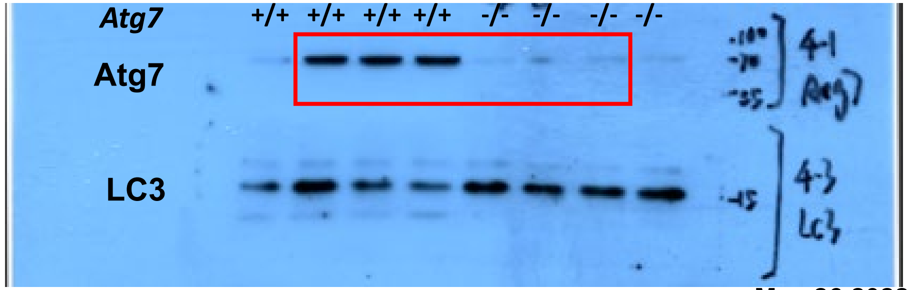

May 26,2022

Long exposure time

Note book6, P6

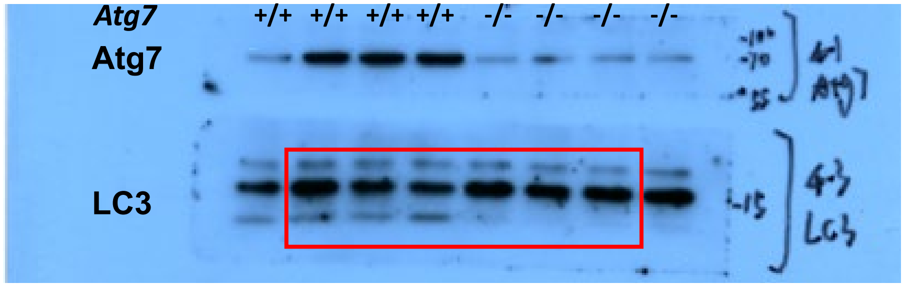

May 26,2022

Unedited blots for Figure 9I

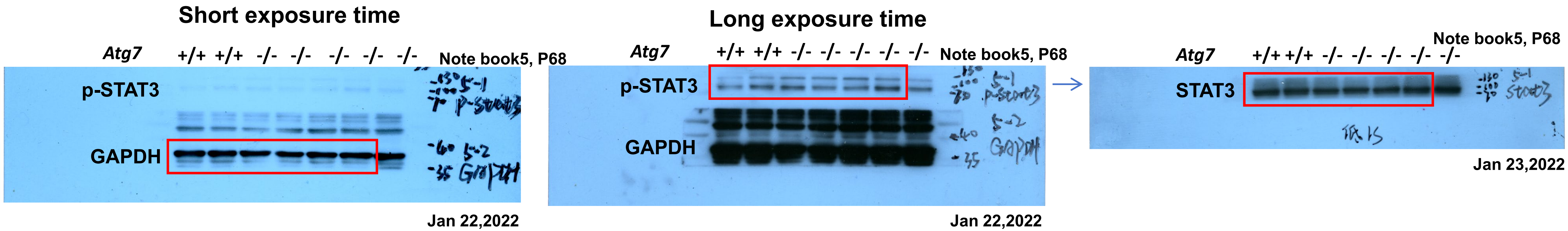

The blot of STAT3 was reprobed from the blot of p-STAT3(Y705)

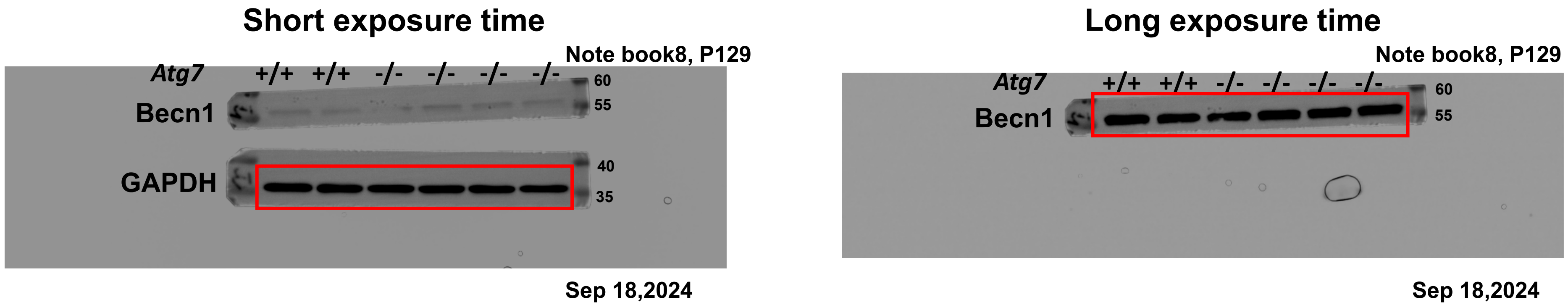

Unedited blots for Figure 9K

Note book5, P72

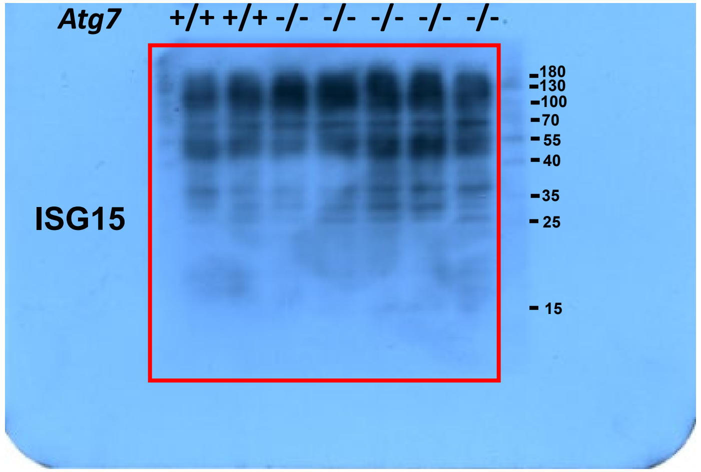

Jan 26,2022

Note book5, P72

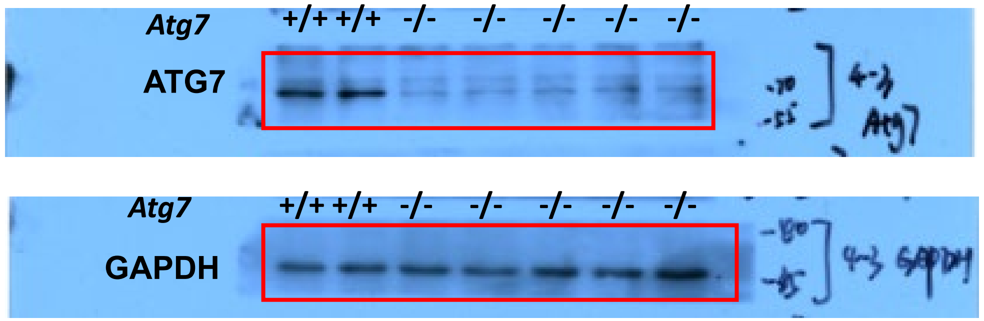

Jan 27,2022

The right panel was reprobed from the left panel

Unedited blots for Figure 9L

Note book6, P65

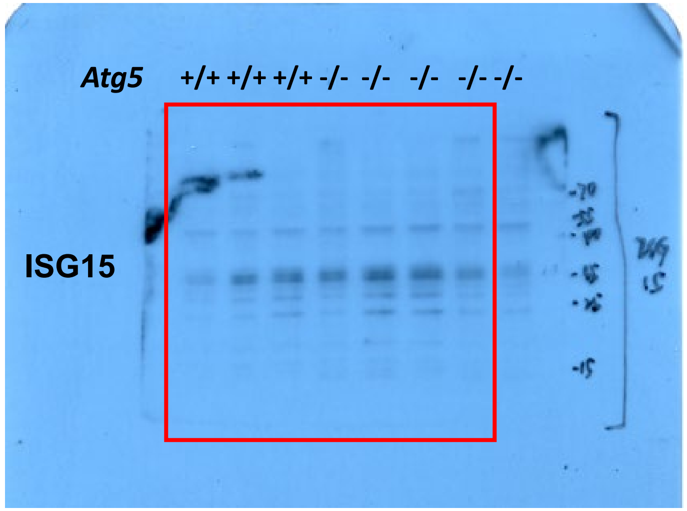

Aug 20,2022

Long exposure time

Note book6, P66

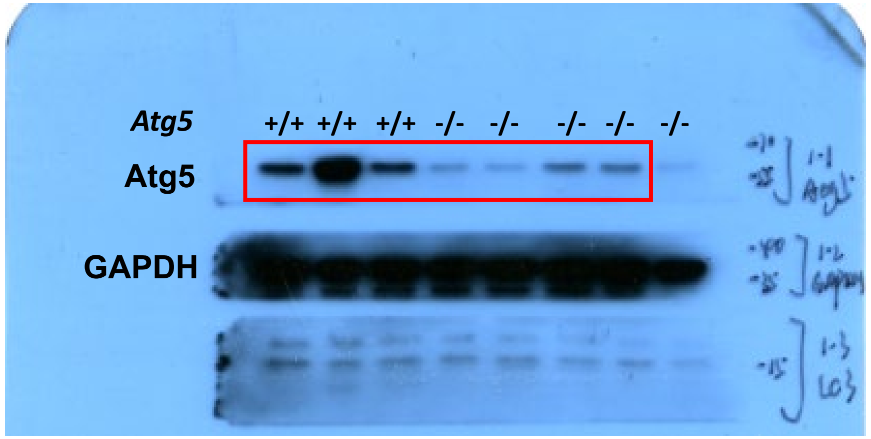

Aug 21,2022

Short exposure time

Note book6, P66

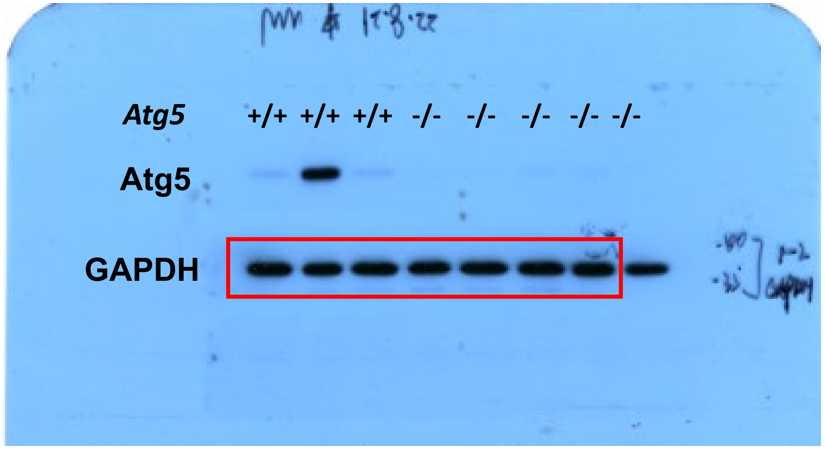

Aug 21,2022

The two right blots were reprobed from the left blot

Unedited blots for Figure S1B

Note book1, P0129581

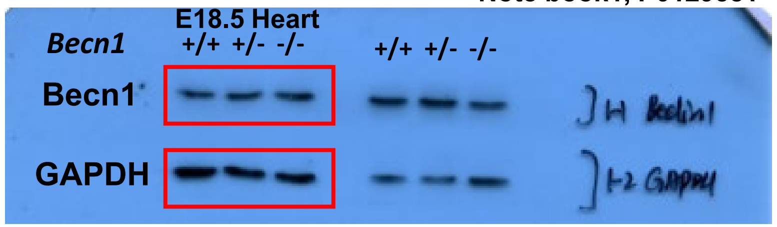

Apr 24,2019

Long exposure time

Note book2, P12

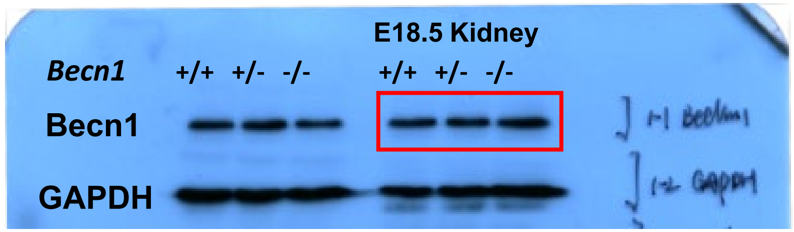

Jun 23,2019

Short exposure time

Note book2, P12

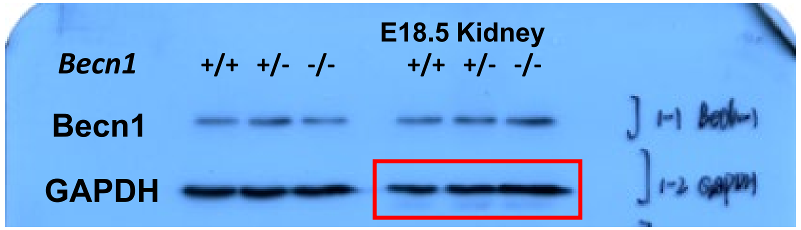

Jun 23,2019

Long exposure time

E18.5 Lung E18.5 Intestine Note book2, P12

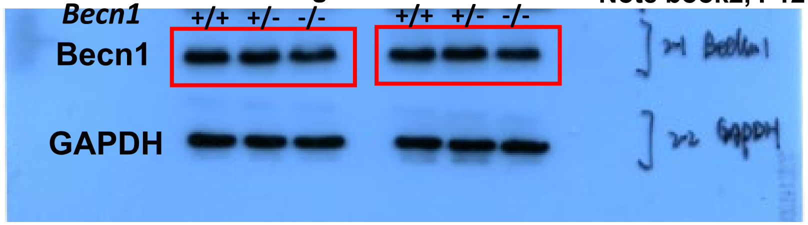

Jun 23,2019

Short exposure time

E18.5 Lung E18.5 Intestine Note book2, P12

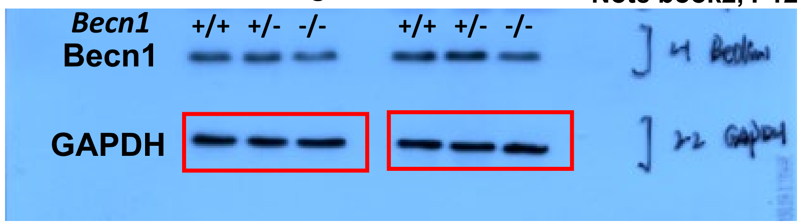

Jun 23,2019

Unedited blots for Figure S8A

Note book5, P35

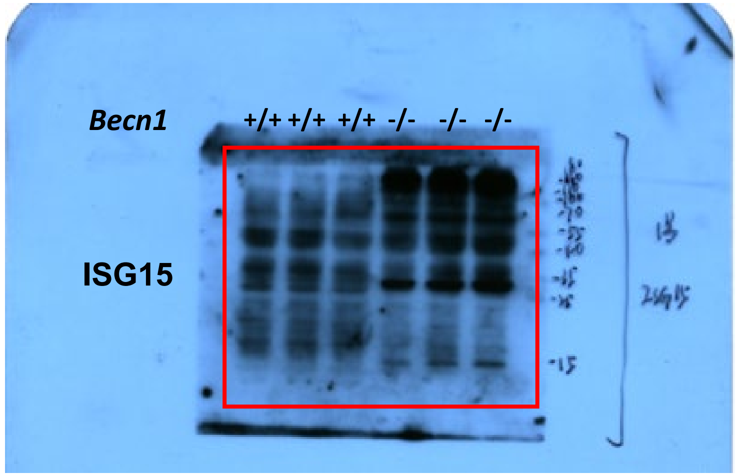

Dec 14,2021

Long exposure time

Note book5, P36

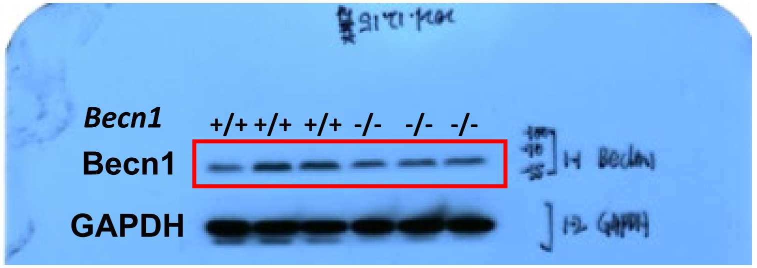

Dec 15,2021

Short exposure time

Note book5, P36

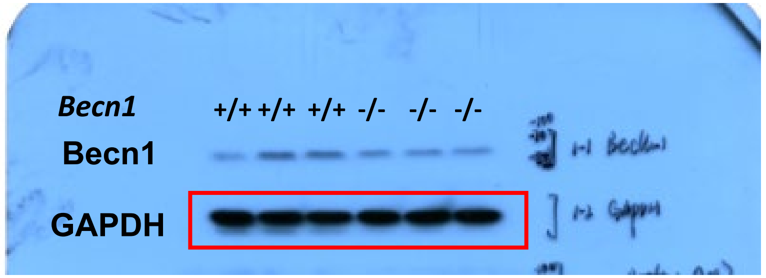

Dec 15,2021

The two right panels were reprobed from the left panel

Unedited blots for Figure S13C

Long exposure time

Note book8, P28

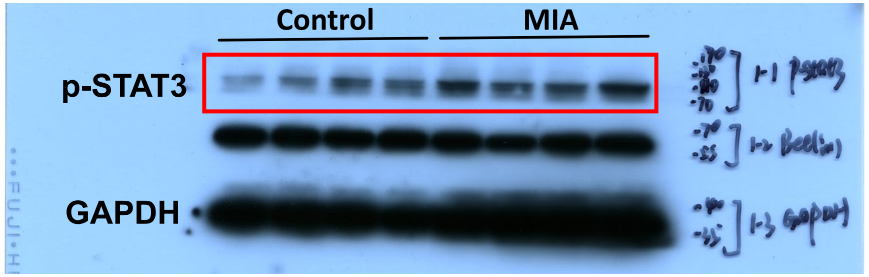

Apr 10,2024

Note book8, P31

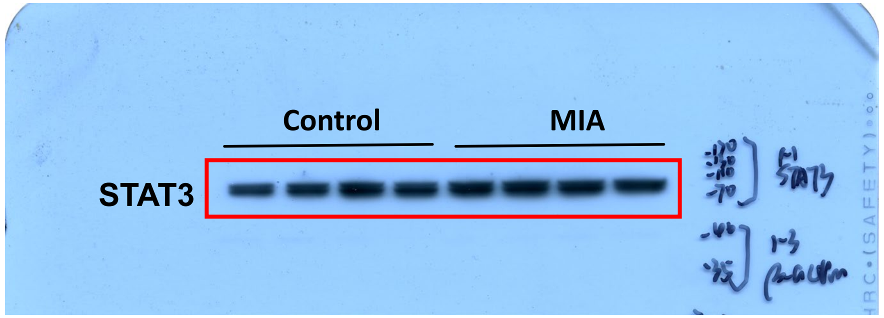

Apr 11,2024

Short exposure time

Note book8, P28

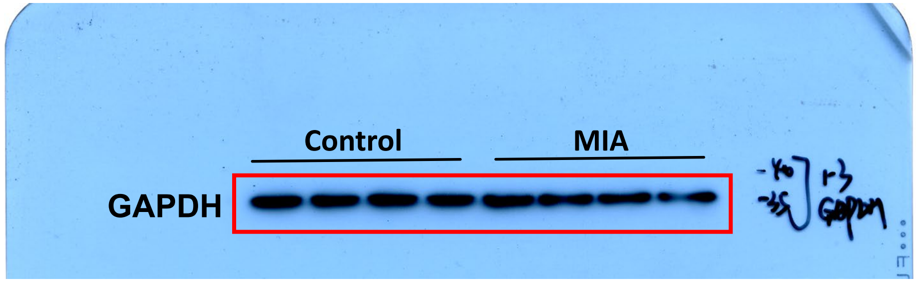

Apr 10,2024

The blot of STAT3 was reprobed from the blot of p-STAT3(Y705)
